# A de novo Gene Promotes Seed Germination Under Drought Stress in Arabidopsis

**DOI:** 10.1093/molbev/msae262

**Published:** 2024-12-24

**Authors:** Guang-Teng Jin, Yong-Chao Xu, Xing-Hui Hou, Juan Jiang, Xin-Xin Li, Jia-Hui Xiao, Yu-Tao Bian, Yan-Bo Gong, Ming-Yu Wang, Zhi-Qin Zhang, Yong E Zhang, Wang-Sheng Zhu, Yong-Xiu Liu, Ya-Long Guo

**Affiliations:** State Key Laboratory of Systematic and Evolutionary Botany, Institute of Botany, Chinese Academy of Sciences, Beijing 100093, China; China National Botanical Garden, Beijing 100093, China; College of Life Sciences, University of Chinese Academy of Sciences, Beijing 100049, China; State Key Laboratory of Systematic and Evolutionary Botany, Institute of Botany, Chinese Academy of Sciences, Beijing 100093, China; China National Botanical Garden, Beijing 100093, China; State Key Laboratory of Systematic and Evolutionary Botany, Institute of Botany, Chinese Academy of Sciences, Beijing 100093, China; China National Botanical Garden, Beijing 100093, China; State Key Laboratory of Systematic and Evolutionary Botany, Institute of Botany, Chinese Academy of Sciences, Beijing 100093, China; China National Botanical Garden, Beijing 100093, China; College of Life Sciences, University of Chinese Academy of Sciences, Beijing 100049, China; State Key Laboratory of Systematic and Evolutionary Botany, Institute of Botany, Chinese Academy of Sciences, Beijing 100093, China; China National Botanical Garden, Beijing 100093, China; College of Life Sciences, University of Chinese Academy of Sciences, Beijing 100049, China; State Key Laboratory of Systematic and Evolutionary Botany, Institute of Botany, Chinese Academy of Sciences, Beijing 100093, China; China National Botanical Garden, Beijing 100093, China; College of Life Sciences, University of Chinese Academy of Sciences, Beijing 100049, China; State Key Laboratory of Systematic and Evolutionary Botany, Institute of Botany, Chinese Academy of Sciences, Beijing 100093, China; China National Botanical Garden, Beijing 100093, China; College of Life Sciences, University of Chinese Academy of Sciences, Beijing 100049, China; State Key Laboratory of Systematic and Evolutionary Botany, Institute of Botany, Chinese Academy of Sciences, Beijing 100093, China; China National Botanical Garden, Beijing 100093, China; College of Life Sciences, University of Chinese Academy of Sciences, Beijing 100049, China; State Key Laboratory of Maize Bio-breeding/College of Plant Protection, China Agricultural University, Beijing 100193, China; State Key Laboratory of Systematic and Evolutionary Botany, Institute of Botany, Chinese Academy of Sciences, Beijing 100093, China; China National Botanical Garden, Beijing 100093, China; College of Life Sciences, University of Chinese Academy of Sciences, Beijing 100049, China; College of Life Sciences, University of Chinese Academy of Sciences, Beijing 100049, China; State Key Laboratory of Integrated Management of Pest Insects and Rodents and Key Laboratory of the Zoological Systematics and Evolution, Institute of Zoology, Chinese Academy of Sciences, Beijing 100101, China; State Key Laboratory of Maize Bio-breeding/College of Plant Protection, China Agricultural University, Beijing 100193, China; China National Botanical Garden, Beijing 100093, China; College of Life Sciences, University of Chinese Academy of Sciences, Beijing 100049, China; Key Laboratory of Plant Molecular Physiology, Institute of Botany, Chinese Academy of Sciences, Beijing 100093, China; State Key Laboratory of Systematic and Evolutionary Botany, Institute of Botany, Chinese Academy of Sciences, Beijing 100093, China; China National Botanical Garden, Beijing 100093, China; College of Life Sciences, University of Chinese Academy of Sciences, Beijing 100049, China

**Keywords:** adaptive evolution, *Arabidopsis thaliana*, de novo gene, regulatory networks

## Abstract

The origin of genes from noncoding sequences is a long-term and fundamental biological question. However, how de novo genes originate and integrate into the existing pathways to regulate phenotypic variations is largely unknown. Here, we selected 7 genes from 782 de novo genes for functional exploration based on transcriptional and translational evidence. Subsequently, we revealed that *Sun Wu-Kong* (*SWK*), a de novo gene that originated from a noncoding sequence in *Arabidopsis thaliana*, plays a role in seed germination under osmotic stress. *SWK* is primarily expressed in dry seed, imbibing seed and silique. *SWK* can be fully translated into an 8 kDa protein, which is mainly located in the nucleus. Intriguingly, *SWK* was integrated into an extant pathway of hydrogen peroxide content (folate synthesis pathway) via the upstream gene *cytHPPK/DHPS*, an Arabidopsis-specific gene that originated from the duplication of *mitHPPK*/*DHPS*, and downstream gene *GSTF9*, to improve seed germination in osmotic stress. In addition, we demonstrated that the presence of *SWK* may be associated with drought tolerance in natural populations of Arabidopsis. Overall, our study highlights how a de novo gene originated and integrated into the existing pathways to regulate stress adaptation.

## Introduction

De novo genes are new genes that originated from the noncoding sequence (Levine et al. 2006; Cardoso-Moreira and Long 2012; Carvunis et al. 2012), which is one of the most important processes to produce new genes. After being discovered in Drosophila, de novo genes have been found in multiple species (Levine et al. 2006; Guo et al. 2007; Guo 2013; Zhao et al. 2014; Ruiz-Orera et al. 2015; Li et al. 2016; Blevins et al. 2021). However, understanding the function of de novo genes and how they integrate into pre-existing molecular pathways is a major challenge (McLysaght and Hurst 2016).

The function of de novo genes is the first crucial question to be addressed. Previously reported de novo genes were mainly involved in reproduction, resistance, toxin response, and metabolic regulation (Singh and Syrkin Wurtele 2020). For instance, the first de novo gene with functional study, *Poldi*, is a positive regulator of sperm motility and testicular weight in the house mouse (Heinen et al. 2009). In plants, *QQS* was the first studied de novo gene, which plays a role in regulating both carbon and nitrogen allocation and pest resistance in Arabidopsis (Li et al. 2009, 2015; Qi et al. 2019). Until now, more than twenty de novo genes have been functionally studied in plants (Jiang et al. 2022). For example, *TaFROG* gene in wheat regulates resistance to Fusarium head blight disease (Perochon et al. 2015), and *Xio1* gene in rice contributes to immune responses to bacterial pathogens (Moon et al. 2022).

How de novo genes are integrated into pre-existing molecular regulatory networks represents another major challenge (McLysaght and Hurst 2016). This involves 2 questions: (i) What are the interacting proteins of the de novo proteins? (ii) What are their upstream and downstream regulatory genes? The first question has attracted considerable attention in previous studies, and interacting partners for several de novo proteins have been identified (Li et al. 2015; Ni et al. 2017; Perochon et al. 2019; Qi et al. 2023). In addition, it has been suggested that proteins of new duplicated genes and de novo genes tend to interact with each other (Capra et al. 2010). However, there are few relevant studies on the upstream and downstream regulatory genes of de novo genes, except that the upstream regulatory gene *Atss3* of *QQS* has been identified (Li et al. 2009). Therefore, it is crucial to understand how de novo genes integrate into existing regulatory pathways.

How de novo genes reach a high-frequency or even become fixed in natural populations, whether adaptive or neutrally, are a fundamental question. The “adaptation following neutrality” theory proposes that positive selection can drive new genes to rapidly evolve important functions (Wu and Zhang 2013). In Drosophila, it has been proposed that natural selection plays an important role in the fixation of de novo genes in natural populations (Zhao et al. 2014). In contrast, many de novo genes may be retained through neutral processes. De novo genes can evolve from “frozen accidents” that survived initial purging (Schmitz et al. 2018). In yeast, there is evidence of hundreds of translated open reading frames that do not show evolutionary conservation or selective constraint (Ruiz-Orera et al. 2018). In the house mouse, similarly, there is no signal of positive selection in the de novo gene *Gm13030*, indicating that a de novo gene can directly exert biological functions without undergoing adaptive mutation (Xie et al. 2019). More broadly, the retention of new mutations is driven by a combination of different factors, such as selection and genetic drift (Barrett and Schluter 2008). The fixation of a new mutation in a population depends on its fitness effects and the strength of selection (Orr 2005; Eyre-Walker and Keightley 2007). Thus, the question of whether functional de novo genes are subject to natural selection requires in-depth evidence.

Although it is widely acknowledged that de novo genes represent an important mechanism for the formation of new genes (Begun et al. 2006; Levine et al. 2006; Chen et al. 2007), the potential role of de novo genes in adaptation remains largely unknown. In Drosophila, a study shows that de novo genes are crucial for survival (Chen et al. 2010). Given the birth of de novo genes occurs at the population level, it is thus important to understand their importance at the population level (Schlötterer 2015). However, whether de novo genes can help natural populations adapt to changing environments is still largely unknown.

Here, we revealed the function of a de novo gene in Arabidopsis, showing how it integrated into pre-existing networks and demonstrating its significance in adaptive evolution. In particular, we demonstrated that this de novo gene, AT1G03106 (*SWK*), enhances *Arabidopsis thaliana* resistance to osmotic stress by regulating hydrogen peroxide signaling. Overall, this study reveals that how a de novo gene originated and integrated into the existing pathways to regulate phenotypic variation and affect adaptation.

## Results

### Sequence Characteristics of Representative de novo Genes

**Fig. 1.**
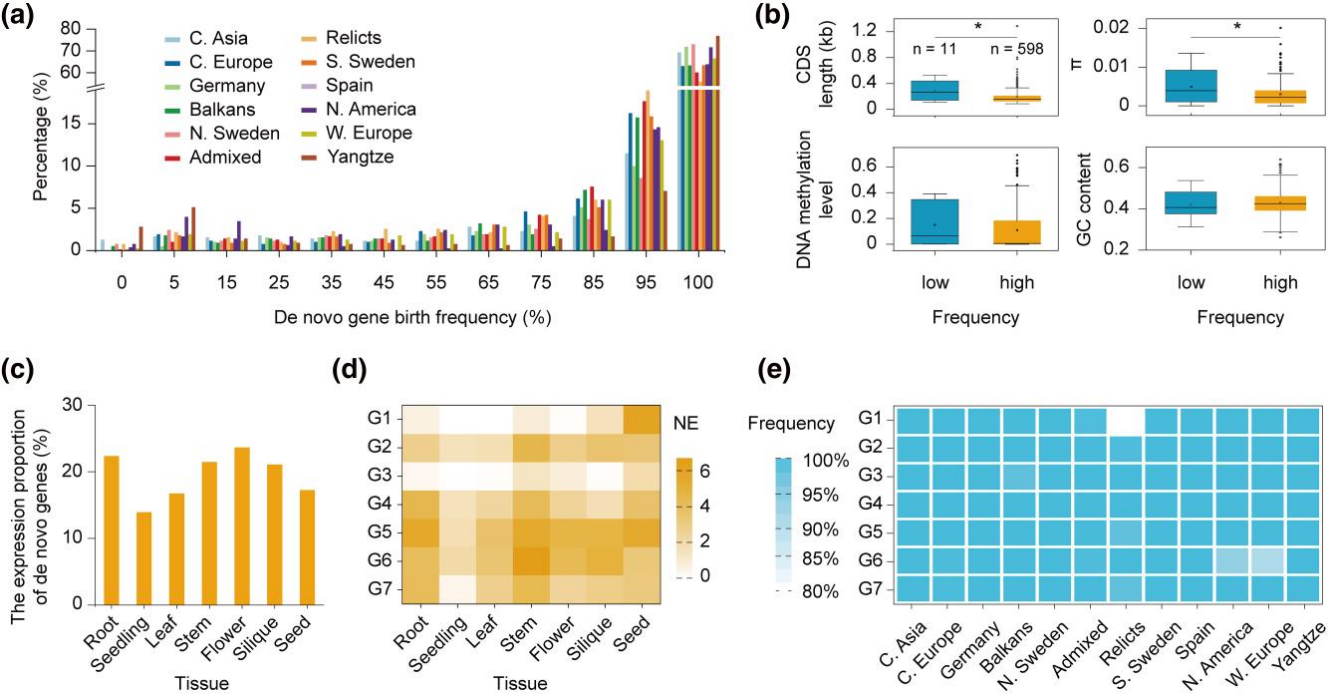
Characteristics of de novo genes and their representatives. a) Frequency distribution of 782 de novo genes in 12 populations. 0: frequency = 0; 100: frequency = 100%; 5: 0 < frequency <10%; 15: 10% ≤ frequency < 20%, with other numbers following the same pattern. C. Asia, Central Asia; C. Europe, Central Europe; N. Sweden, North Sweden; S. Sweden, South Sweden; N. America, North America; W. Europe, Western Europe; Yangtze, Yangtze River. b) Comparison of gene features between low-frequency (frequency < 10%) and high-frequency (frequency ≥ 90%) de novo genes in 1,115 accessions. Nucleotide polymorphism (π), CDS length, GC (Guanine-Cytosine) content, and DNA methylation level were calculated based on the Col-0 reference. Wilcoxon rank-sum test was used for testing significance, *, *P* < 0.05. c) The percentage of 782 de novo genes expressed in 7 different tissues. Expression is defined as TPM > 1. d) The expression patterns in different tissues of 7 representative genes for functional exploration. G1, AT1G03106; G2, AT1G58150; G3, AT2G07000; G4, AT2G13550; G5, AT2G14460; G6, AT4G36515; G7, AT5G67245. NE, Normalized expression, log_2_(TPM + 1). e) Frequency of the 7 representative de novo genes in 12 populations.

In a previous study, we identified 782 de novo genes in Arabidopsis (Col-0) (Li et al. 2016). Based on the published resequencing data of 1,115 natural accessions from 12 populations (Cao et al. 2011; The 1001 Genomes Consortium 2016; Durvasula et al. 2017; Zou et al. 2017; Jiang et al. 2024), between 74.8% and 86.2% of the 782 de novo genes were nearly fixed (frequency >90%) in at least one of the 12 populations (Fig. 1a). Among these populations, the North America population has the largest proportion (86.2%) of nearly fixed de novo genes (frequency >90%) (Fig. 1a). In contrast, Yangtze River basin population has the highest proportion of low-frequency (frequency <10%) de novo genes (5.1%) (Fig. 1a).

The comparison of gene features between low-frequency (frequency <10%) and high-frequency (frequency ≥90%) de novo genes showed that the coding sequence (CDS) length and nucleotide polymorphism (π) of low-frequency de novo genes were significantly higher than those of high-frequency de novo genes (Wilcoxon rank-sum test, *P* < 0.05, Fig. 1b). This indicates that low-frequency de novo genes are longer and more polymorphic. However, other gene features, such as gene length, GC (Guanine-Cytosine) content, DNA methylation level, the ratio of nonsynonymous to synonymous nucleotide diversity (π_n_/π_s_), codon usage bias, and the density of accessible chromatin regions, were not significantly different (Fig. 1b and supplementary fig. S1a, Supplementary Material online). Our results are consistent with reports in Drosophila indicating that the exon lengths of segregating de novo genes are significantly longer than those of fixed de novo genes (Zhao et al. 2014), supporting the preadaptation hypothesis of de novo gene origin (Wilson et al. 2017). According to RNA sequencing data from different tissues, de novo genes are mainly expressed in flower, root, stem, and silique, followed by the seed (Fig. 1c). This pattern differs slightly from previous studies in rice (callus, shoot, and flower) and mangrove (root, fruit, and flower) (Guo et al. 2007; Ma et al. 2021). Notably, the percentage of de novo genes expressed in the flowers, is exceptionally high, which is similar to the higher percentage of de novo genes expressed in animal testis (Levine et al. 2006; An et al. 2023).

According to the transcriptional and translational evidence in Col-0, these 782 de novo genes were divided into 3 groups (Li et al. 2016): 398 de novo genes without any evidence of transcription or translation (TN); 239 de novo genes with only transcriptional evidence (TC); 145 de novo genes with translation evidence (TL). Given that polyadenylation plays an important role in translation initiation (Wells et al. 1998), TL-type de novo genes could be classified into 2 types according to the presence or absence of polyadenylation: 80 TL type genes without poly(A) tails (TL^−^); 65 TL type with poly(A) tails (TL^+^) (Li et al. 2016). In total, we selected 6 TL^+^ type and 1 TC type de novo gene that can be expressed (Transcripts Per Million (TPM) >1) in multiple tissues to perform in-depth study (supplementary fig. S1b, Supplementary Material online). In detail, the 7 genes are mainly expressed in root, stem, flower, silique, or seed (Fig. 1d). Among the 7 de novo genes, except for AT1G03106 (G1) in the Relicts population (frequency = 79.0%) and AT4G36515 (G6) in the Western Europe population (frequency = 89.6%), the other 5 genes were nearly fixed in each population (frequency >90%, Fig. 1e).

### Functional Characterization of Representative de novo Genes

**Fig. 2.**
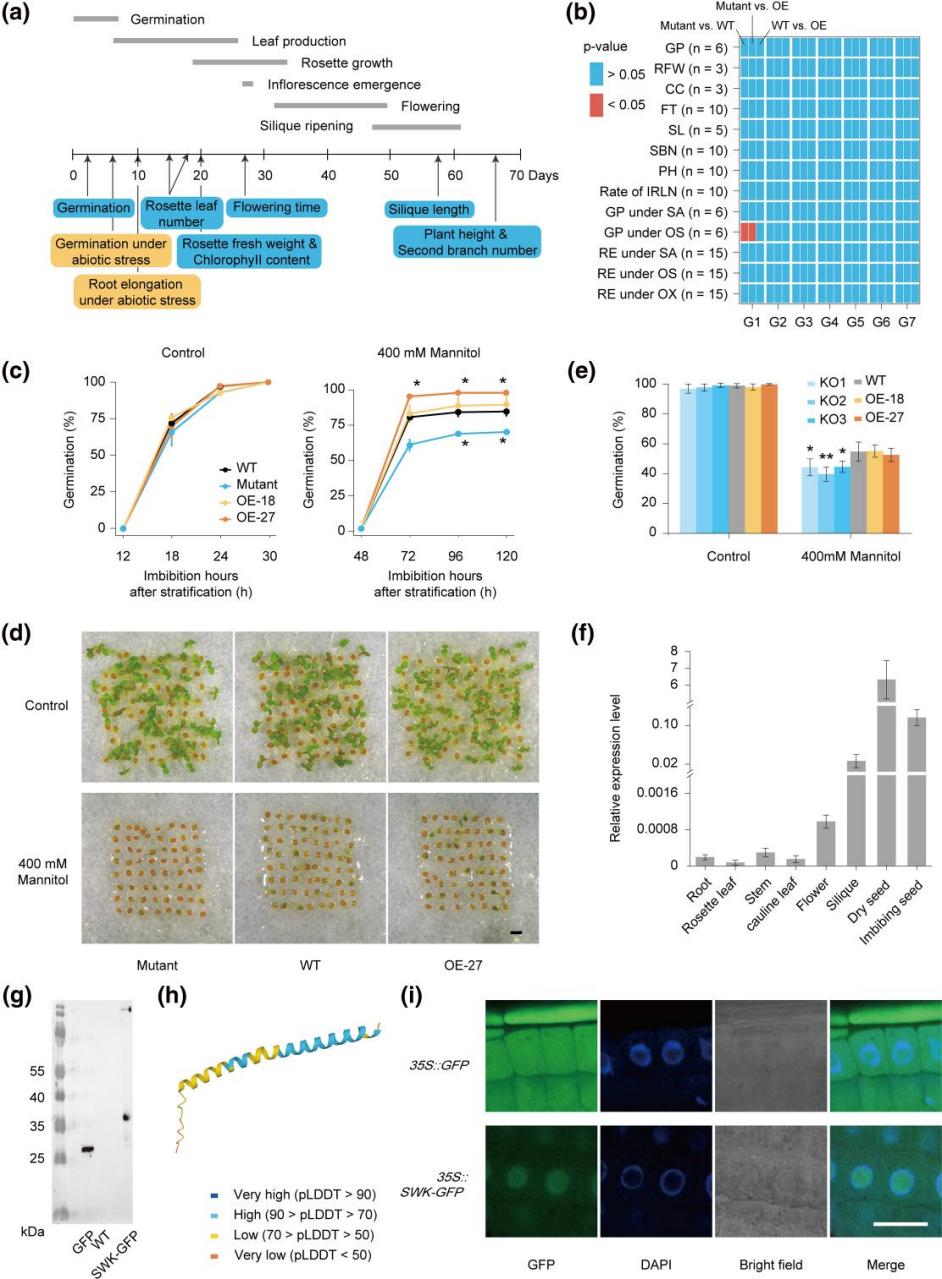
Phenotyping of representative de novo genes. a) Traits measured in this study and timings. The numbers under the line represent days after sowing. The boxes represent phenotypes throughout the life cycle (blue) and under abiotic stress (yellow). The gray horizontal line represents the developmental period marked by the same horizontal line. b) Pairwise phenotypic comparison of mutant and Col-0 (WT) or OE. GP, seed germination percentage; RFW, Rosette fresh weight; CC, chlorophyII content; FT, flowering time; SL, silique length; SBN, number of secondary branches; PH, plant height; Rate of IRLN, rate of increase in rosette leaf number; GP under SA, seed germination percentage under salt stress; GP under OS, seed germination percentage under osmotic stress; RE under SA, root elongation under salt stress; RE under OS, root elongation under osmotic stress; RE under OX, root elongation under oxidative stress. *n*, the number of plants used for phenotyping. c) Seed germination percentage under mannitol stress. Percentages of seed germination are means (±SD) based on the seeds from 6 individual plants. Either mutant or OE was compared to WT at the same time point. d) Images showing seed germination of *SWK* mutants, WT, and OE-27 under control and 400 mM mannitol stress after 4 d of growth. Scale bar, 1 mm. e) Seed germination percentage of knockout mutants (KOs). Percentages of seed germination are means (±SD) based on the seeds from 6 individual plants. The statistical time of the control group was after 1 d of growth, while the statistical time of the mannitol stress treatment group was after 5 d of growth. All KOs were compared to WT. f) RT-qPCR analysis of *SWK* expression in 8 tissues of WT. Expression values are means (±SD) based on 3 replicates per line. g) Western blotting showing the expression of SWK constructs in seed. Lanes from left to right are as follows: markers, seeds transfected with GFP, WT, SWK-GFP fusion protein of OE (SWK-GFP). h) SWK protein structure predicted by AlphaFold2. pLDDT, predicted local distance difference test. i) Subcellular localization of SWK in the root tip. The images were obtained from the GFP channel, DAPI channel, Bright channel, and a merged image of the 3 channels. Scale bar, 20 μm. Two-sided Student's *t*-test was used for significance test. *, *P* < 0.05; **, *P* < 0.01.

In order to explore the function of these 7 de novo genes, we obtained the T-DNA insertion mutants and constructed overexpression (OE) lines in the Col-0 background (supplementary fig. S1c and S1d, Supplementary Material online). We measured 8 phenotypes throughout the life cycle, including seed germination, rate of increase in rosette leaf number, rosette fresh weight, chlorophyll content, flowering time, silique length, plant height and number of secondary branches, using the mutant, Col-0 (WT), and OE lines (Fig. 2a). Furthermore, 5 phenotypes under stress conditions were assessed after treatment in Petri dishes: seed germination and root elongation under salt stress, seed germination and root elongation under osmotic stress, and root elongation under oxidative stress (Fig. 2a).

Among all the phenotypes examined for these 7 de novo genes, only seed germination under osmotic stress for the comparison of AT1G03106 mutant (G1) and its WT or OE lines showed a significant difference (two-sided Student's *t*-test, Benjamini–Hochberg correction, *P* < 0.05, Fig. 2b). Under osmotic stress (400 mM mannitol), the seed germination of the OE and WT plants was significantly higher than that of the mutants (two-sided Student's *t*-test, Benjamini–Hochberg correction, *P* < 0.05, Fig. 2c and 2d, and supplementary fig. S2a, Supplementary Material online). Hereafter, we referred to AT1G03106 as *Sun Wu-Kong* (*SWK*, the Monkey King born from a stone in “*Journey to the West*”). Given that ABA is a key hormone affecting germination, we estimated seed germination under exogenous ABA stress, and found that the seed germination of mutants was significantly higher than that of an OE line (two-sided Student's *t*-test, Benjamini–Hochberg correction, *P* < 0.05, supplementary fig. S2b, Supplementary Material online). The effect of the 2 different stresses on the phenotype suggested that *SWK* probably regulates seed germination under either osmotic stress or ABA stress.

To validate the function of *SWK*, besides the available T-DNA insertion mutant, we generated 3 *SWK* mutants using the CRISPR/Cas9 system in Col-0 background (supplementary fig. S2c and S2d, Supplementary Material online). The knockout mutants do not affect the protein structure of adjacent genes (supplementary fig. S2c, Supplementary Material online), and adjacent genes are not involved in regulating germination under osmotic stress according to previous reports. Based on the 3 different homozygous edited lines with predicted truncated proteins, we found that seed germination of the knockout mutants under osmotic stress was significantly lower than in the wild type, further confirming the function of *SWK* (Fig. 2e).

### Expression Pattern and Subcellular Localization of *SWK*

To reveal the expression pattern of *SWK*, we estimated its expression level in 8 different tissues, including root, rosette leaf, stem, cauline leaf, flower, silique, dry seed, and imbibing seed. The results indicated that *SWK* is expressed in all these 8 tissues, including leaf tissue, but highly expressed in dry seed, imbibing seed, and silique (Fig. 2f), indicating that *SWK* likely plays a role in seed development processes.

To verify whether the de novo CDS of *SWK* can produce a complete protein, we constructed a SWK-GFP (GFP, Green fluorescent protein) vector by fusing the CDS of GFP to the C-terminus of SWK, which is predicted to produce a GFP-tagged SWK fusion protein when transformed into Col-0. Western blots indicated that the mass of the fusion protein was increased by 8 kDa (total 35 kDa) as compared to that of GFP (27 kDa), consistent with the predicted size of the SWK protein (Fig. 2g).

SWK is composed of an alpha helix and a disordered sequence based on protein structure modeling using AlphaFold2 (Fig. 2h), as expected for a younger protein. In addition, the GFP-tagged SWK overexpressing plants showed that SWK mainly localizes at the nucleus, but is also present in the cytoplasm (exclude chloroplasts) and the cell membrane (Fig. 2i and supplementary fig. S2e, Supplementary Material online). Localization of SWK in the nucleus is consistent with the characteristics of transcription factors (Puertollano et al. 2018), while the localization of SWK on the cell membrane supports the tendency of new genes to encode putative membrane domains (Cui et al. 2015; Vakirlis et al. 2020).

### Identification of Upstream Genes of *SWK*

**Fig. 3.**
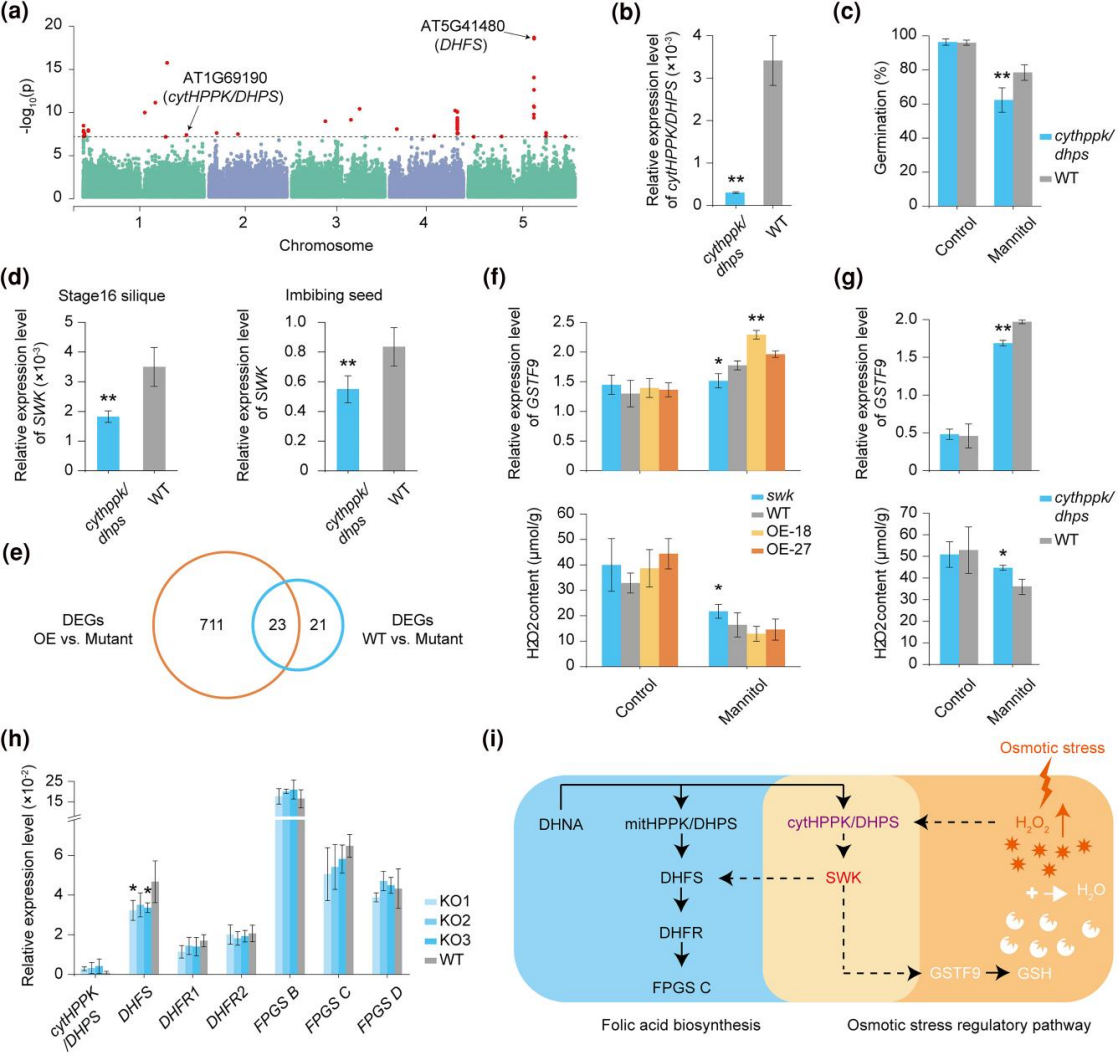
Upstream and downstream regulatory genes of *SWK*. a) eGWAS analysis based on *SWK* expression levels and whole-genome variation in 414 *A. thaliana* accessions. The dashed line represents the threshold line [−log_10_ (0.05/1,133,651)]. The 2 genes marked are the genes in the folate biosynthesis pathway. b) Expression difference between the *cytHPPK/DHPS* mutant and WT. Values are means (±SD) based on 3 replicates per line. c) Differences in seed germination percentages between the *cytHPPK/DHPS* mutants and WT under mannitol stress. Percentages of seed germination are means (±SD) based on the seeds from 6 individual plants. d) *SWK* expression level in the *cytHPPK/DHPS* mutant and WT. The imbibing seeds are under the stress of 400 mM mannitol. Values are means (±SD) based on 3 replicates per line. e) Number of DEGs in the comparison of the *SWK* mutant with WT or OE by RNA-seq analysis. f) Expression levels of *GSTF9* and H_2_O_2_ content differences in *SWK* mutant, WT and OE under 400 mM mannitol stress. Values are means (±SD) based on 3 replicates per line. Either mutant or OE was compared to WT. g) Expression levels of *GSTF9* and H_2_O_2_ content differences in the *cytHPPK/DHPS* mutant and WT under mannitol stress. Values are means (±SD) based on 2 replicates per line. h) Comparison of expression levels of folate biosynthesis genes in the *SWK*-knockout mutants (KOs), WT, and OE lines. Values are means (±SD) based on three replicates per line. All KOs were compared to WT. i) Schematic diagram of the 2 pathways involving *cytHPPK/DHPS* and *SWK*. The black solid arrows indicate direct regulation; the black dotted arrows suggest regulation, but it is not confirmed whether there are intermediate genes; the white arrow and plus sign denote chemical reaction; the red lightning symbolizes osmotic stress stimulation, and the arrow indicates an increase in the signaling factor. Two-sided Student's *t*-test was used for significance test. *, *P* < 0.05; **, *P* < 0.01.

In order to find the upstream regulatory genes of *SWK*, we used RNA-seq data of leaf tissues from 414 (including Col-0) natural accessions (Kawakatsu et al. 2016) to perform a genome-wide association analysis for the trait of *SWK* expression level (eGWAS, Fig. 3a). To enhance the reliability of the results based on SNPs, we used 2 different software programs based on both SNPs and INDELs at 2 different minor allele frequency (MAF) thresholds (1% and 5%) (supplementary fig. S3, Supplementary Material online), and found that the results are consistent using different genotype data sets, programs or MAF thresholds. There are 66 significant SNPs in the eGWAS for *SWK*, which involve 61 genes (supplementary tables S1 and S2, Supplementary Material online). In contrast, for the other 6 de novo genes studied, the significant signals in eGWAS were mainly located near the genes themselves, except for the gene AT2G07000 (supplementary table S1, Supplementary Material online, supplementary fig. S3e, Supplementary Material online). Taken together, these results suggested that *SWK* may have built up interactions with other genes, while the other 6 de novo genes not. This is consistent with the fact that the other 6 genes do not show any phenotypic effect on the traits measured in this study.

Among the 61 candidate genes associated with *SWK* expression variation, it has been reported that AT1G69190 (*cytHPPK/DHPS*) could increase the seed germination of Arabidopsis under mannitol stress (Storozhenko et al. 2007). Here in this study, the mutant analysis of *cytHPPK/DHPS* confirmed that this gene, indeed, could increase seed germination under mannitol stress (Fig. 3b and c and supplementary fig. S4a, Supplementary Material online). In addition, the expression level of *SWK* is significantly reduced in the *cytHPPK/DHPS* mutant background, which implied that *cytHPPK/DHPS* is an upstream regulator of *SWK* (Fig. 3d and supplementary fig. S4b, Supplementary Material online). In contrast, *DHFS*, which displays the strongest eGWAS signal, does not alter *SWK* expression following *DHFS* knockdown (Fig. 3a and supplementary fig. S4c, S4d, and S4e, Supplementary Material online), suggesting that it is not likely an upstream regulatory gene of *SWK*.

In addition to eGWAS, the RNA-seq database provides another way to identify the upstream regulatory genes of a target gene (http://ipf.sustech.edu.cn/pub/athrna/). Based on the RNA-seq data of different mutants in the database, we screened genes that could change the expression of *SWK* by more than 2-fold in knockout mutants, which are potential candidate regulatory genes upstream of *SWK*.

In total, *SWK* expression was significantly changed in mutants of 71 genes (supplementary table S3, Supplementary Material online). Four of them are involved in regulating the seed germination under osmotic stress, and 22 genes are involved in regulating the seed germination under other conditions. The KEGG pathway enrichment results showed that these 71 genes were significantly enriched in 4 KEGG pathways (*P* < 0.05), including the circadian rhythm-plant, plant hormone signal transduction, lysine degradation, phosphonate and phosphinate metabolism (supplementary fig. S4f, Supplementary Material online, supplementary table S4, Supplementary Material online).

### Identification of Downstream Genes of *SWK*

To identify the downstream genes of *SWK*, we extracted RNA from imbibing seeds of the mutant, Col-0 and OE-27 under both control and osmotic stress treatments after 12 h of lighting for RNA-seq. Given the phenotype of the *swk* mutant is significantly different from that of the WT and OE, we took the shared differentially expressed genes (DEGs) between the mutant and the WT, and between the mutant and the OE under stress treatment, as candidate downstream genes (Fig. 3e and supplementary table S5, Supplementary Material online). There are 23 candidate *SWK* downstream genes, of which 2 are involved in the regulation of osmotic stress responses, 2 are involved in the regulation of germination, and 4 genes are involved in the regulation of other abiotic stress responses (supplementary table S5, Supplementary Material online). The KEGG pathway enrichment results showed that these 23 genes were significantly enriched in 6 KEGG pathways (*P* < 0.05), including the glucosinolate biosynthesis, tryptophan metabolism, 2-oxocarboxylic acid metabolism, metabolic pathways, biosynthesis of secondary metabolites, and glutathione metabolism (supplementary fig. S4g, Supplementary Material online, supplementary table S6, Supplementary Material online).

Of these 23 genes, we focused on 2 candidate genes, *GSTF9* (Nguyen et al. 2020) and *GSTF10* (Ryu et al. 2009), which are known to be involved in the glutathione metabolic pathway and the osmotic stress response. To confirm whether these 2 candidate genes were downstream genes of *SWK*, we estimated their expression levels in the *SWK* mutant, WT and OE. The results suggest that OE showed higher *GSTF9* expression than WT, and WT showed higher expression than mutant under stress conditions (two-sided Student's *t*-test, Benjamini–Hochberg correction, *P* < 0.05), but *GSTF10* expression did not differ significantly under stress conditions (Fig. 3f and supplementary fig. S4h, Supplementary Material online). Given that *GSTF9* increases plant resistance to mannitol stress by degrading hydrogen peroxide (Nguyen et al. 2020), we measured the hydrogen peroxide content of *SWK* mutant, WT and OE under mannitol stress and found that the mutant has significantly higher levels than either WT or OE (two-sided Student's *t*-test, Benjamini–Hochberg correction, *P* < 0.05, Fig. 3f).

In addition, given that *cytHPPK/DHPS* regulates *SWK* expression, we detected a significant difference in the expression of *SWK* in the *cytHPPK/DHPS* mutant. In *cytHPPK/DHPS* mutant background, *GSTF9* is expressed in a similar pattern to *SWK*, suggesting that *cytHPPK/DHPS* acts as an upstream regulatory gene of *GSTF9* (Fig. 3g). *cytHPPK/DHPS* is an Arabidopsis-specific gene that was generated by the duplication of the folic acid biosynthetic pathway gene *mitHPPK/DHPS* (Storozhenko et al. 2007; Gorelova et al. 2019). Therefore, we estimated the expression level of these genes in the folate biosynthesis pathway in both the *SWK*-knockout and WT backgrounds. Intriguingly, the expression of *DHFS* in the *SWK*-knockout lines was significantly lower than that in WT, indicating that *SWK* is involved in the folate biosynthesis pathway and acts as the upstream regulatory gene of *DHFS* (Fig. 3h). The expression of genes downstream of *DHFS* did not change, possibly due to the strong feedback inhibition by downstream enzymes (Mouillon et al. 2002; Hanson and Gregory 2011). However, the *cytHPPK/DHPS* mutant can affect the folate content (Storozhenko et al. 2007). It appears that *SWK* is not the only downstream gene of *cytHPPK/DHPS* involved in folate biosynthesis. Taken together, these functional analyses illustrate an instance where a de novo gene and a duplication-generated new gene collaborate to integrate into a regulatory network in plants (Fig. 3i). Subsequently, we found that none of the 3 upstream and downstream proteins directly interact with SWK through yeast two-hybrid experiments (supplementary fig. S4i, Supplementary Material online). Overall, these findings highlight the complex interactions between de novo and duplication-generated genes, demonstrating their collaboration during the integration process into plant regulatory networks despite the lack of direct protein-protein interactions.

### The Origin and Evolution of *SWK*

**Fig. 4.**
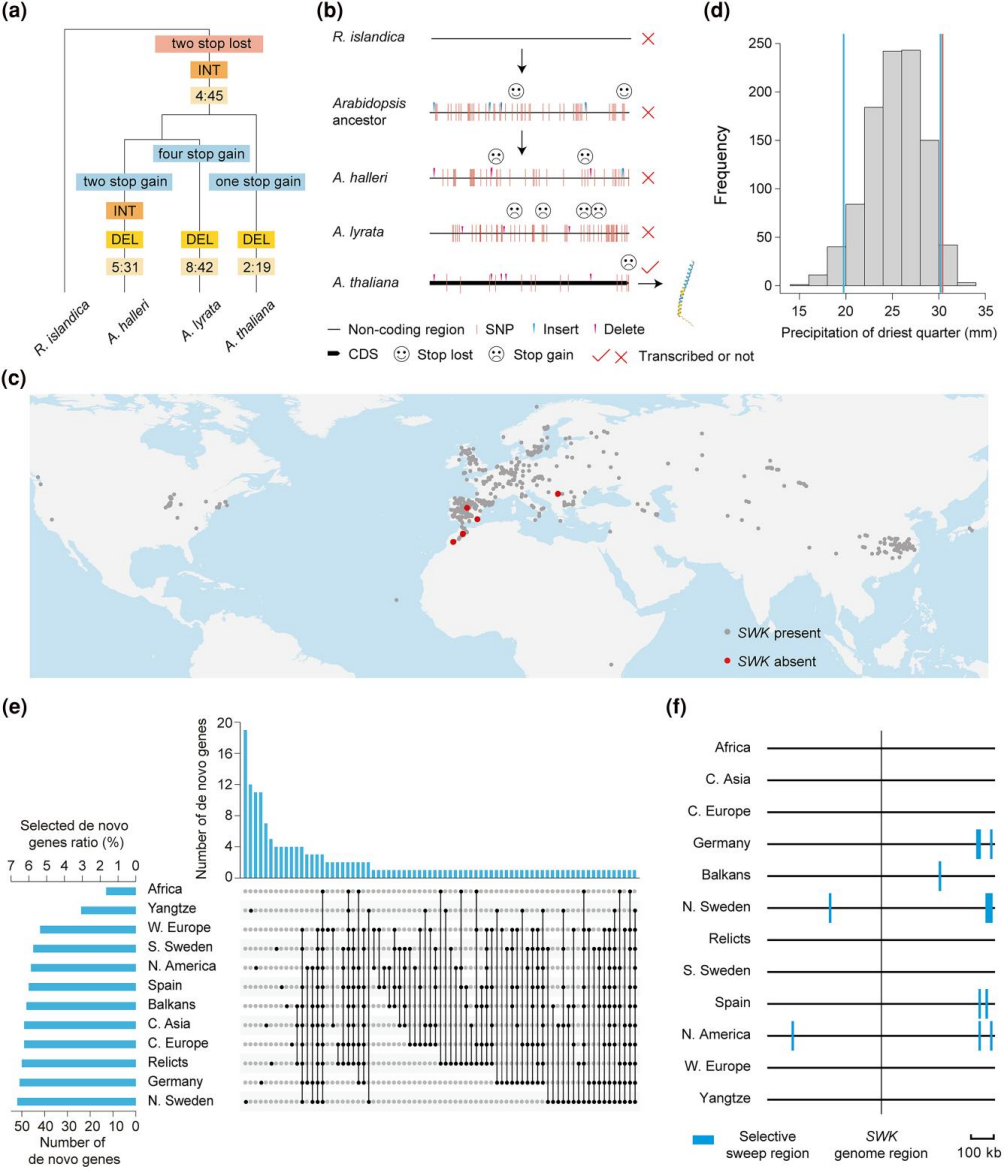
Evolutionary analysis of *SWK*. a) The sequences with the highest similarity to the CDS of *SWK* in *A. lyrata*, *A. halleri*, and *R. islandica* were compared to analyze the evolutionary history of *SWK*. Numbers refer to synonymous: nonsynonymous substitutions. INT, insertion; DEL, deletion. b) The evolutionary history of *SWK*, with types of sequence variations are annotated according to the alignment results in supplementary fig. S5b, Supplementary Material online. c) Distribution of *SWK* in natural accessions worldwide. d) A 1,000-permutation test was used to generate the expected distribution of the difference in bio17 (precipitation of the driest quarter) between *SWK*-present and *SWK*-absent accessions in Africa. The area between the 2 blue vertical lines represents the 95% confidence interval. The red vertical line represents the bio17 average of 5 *SWK*-absent accessions. e) This panel shows the number of selected de novo genes in different populations and the number of selected de novo genes shared by different populations. f) Selective sweep regions of *SWK* and its upstream and downstream regions (500 kb each) in 12 natural populations based on linkage disequilibrium.

To trace the evolutionary history of *SWK*, we performed rigorous blast searches against a wide array of genomic databases (Phytozome 13, MycoCosm, PhycoCosm, IMG/M, and Ensembl 109). The homologous sequence of the *SWK* ORF was only found in 4 species of Brassicaceae family, including Arabidopsis, *Arabidopsis lyrata*, *Arabidopsis halleri*, and *Rorippa islandica* (supplementary fig. S5a, Supplementary Material online). Based on orthologous sequences of *SWK* from Arabidopsis, *A. lyrata*, and *A. halleri*, we used FastML (Ashkenazy et al. 2012) to infer the common ancestral sequence of *Arabidopsis* genus (supplementary fig. S5b, Supplementary Material online). Then, we inferred the evolutionary process of *SWK*, based on the alignment of *SWK* from 3 species of *Arabidopsis* genus, the common ancestral sequence of *Arabidopsis* genus, and *R. islandica*, with *R. islandica* as outgroup. We identified 2 stop codons that were lost during the transition from *R. islandica* to the common ancestor of the *Arabidopsis* genus, using *R. islandica* as outgroup (supplementary fig. S5c, Supplementary Material online). The loss of 2 stop codons due to base substitutions extended the length of the ORF (Fig. 4a and b and supplementary fig. S5b, Supplementary Material online). Subsequently, 19 nonsynonymous and 2 synonymous substitutions occurred in Arabidopsis, giving rise to *SWK* (Fig. 4a and b and supplementary fig. S5b, Supplementary Material online). However, the formation of new premature stop codons in *A. lyrata*, and *A. halleri* resulted in a truncated ORF (Fig. 4a and b and supplementary fig. S5b, Supplementary Material online).

In order to further reveal the evolutionary history of *SWK*, we extracted the orthologous sequences flanking *SWK* in the representative species of Brassicaceae family with a genome assembly. The 5 genes, located either upstream or downstream of *SWK*, are largely syntenic in these species, but there is no homolog sequence of *SWK* at the syntenic region, except in Arabidopsis (supplementary fig. S5d, Supplementary Material online). Multiple Helitron and MuDR type transposable elements are found within 2 kb upstream and downstream of the homolog in Arabidopsis (supplementary fig. S5e, Supplementary Material online). Except for Helitron, transposable elements could form a target site duplication (TSD) of generally 4 to 10 bp on both sides of the insertion site (Wicker et al. 2007; Tan et al. 2016). Here, we found remnants of TSD (CATGGC) at 167 and 232 bp upstream of the *SWK* start codon in Arabidopsis, which can also be observed in closely related species, *A. lyrata* and *A. halleri* (supplementary fig. S5f, Supplementary Material online). These findings suggest that copy number changes of *SWK* homologs may have been caused by ancient transposition events. Based on the phylogenetic tree of Brassicaceae (Hendriks et al. 2023), Arabidopsis is closely related with other species (e.g. *Capsella rubella*, *Malcolmia maritima*, *Nevada holmgrenii*, and *Nerisyrenia camporum*) that lack homologous *SWK* sequences or remnants, than that of *R. islandica*. Given the complete absence of *SWK* in these species, horizontal gene transfer (HGT) between *R. islandica* and Arabidopsis is more likely the explanation according to the principle of parsimony. In addition, previous studies have documented HGT between distantly related species, such as between tomato and Arabidopsis (Cheng et al. 2009).

Based on sequence analysis of the *SWK* CDS in different populations using published genomic data of 1,115 natural accessions of 12 different populations (Cao et al. 2011; The 1001 Genomes Consortium 2016; Durvasula et al. 2017; Zou et al. 2017; Jiang et al. 2024), we found that *SWK* was present in nearly all of the natural accessions except for 8 accessions located in the Mediterranean region (Fig. 4c). Every *SWK*-absent natural accession contains only 1 or 2 variants (SNPs/Indels) compared to Col-0 (supplementary fig. S6, Supplementary Material online). These mutations either disrupt stop codons, introduce premature stop codons, or lead to the loss of start codon, while the rest of the sequence remains largely conserved. Based on the limited number of variant sites within accessions and the small number of variant accessions (8/1115), we speculate that these accessions with nonfunctional allele of *SWK* more probably represent a subsequent loss of *SWK* following its formation.

Given the African population is one of the oldest natural populations and represents the early polymorphism of Arabidopsis (Durvasula et al. 2017), we chose the African population to explore the potential role of *SWK* in adaptation. There are 60 natural accessions in the African population, 55 of which have formed *SWK*, and the other 5 accessions not (Fig. 4c and supplementary fig. S6, Supplementary Material online). Comparison of the 19 environmental factors between the habitat of *SWK*-present accessions and *SWK*-absent accessions in these 60 accessions from Africa (supplementary fig. S7a, Supplementary Material online, supplementary tables S7 and S8, Supplementary Material online) suggested that the precipitation of the driest quarter (bio17) in the *SWK*-present accessions was significantly lower than those in the *SWK*-absent accessions (two-sided Student's *t*-test, *P* < 0.001, supplementary fig. S7b, Supplementary Material online). To avoid false positives caused by sample number, we randomly selected bio17 from 5 samples, repeated 1,000 times from 55 *SWK*-present accessions, and compared bio17 with 5 SWK-absent accessions; the results suggested that the bio17 values were still significantly different from those of the *SWK*-absent accessions (*P* < 0.05, Fig. 4d). These results align with the functional characteristics of *SWK* in enhancing the drought stress resistance of Arabidopsis induced by mannitol. Additionally, we performed Redundancy Analysis (RDA) to investigate the association between genomic variation in natural accessions from African populations and environmental factors of their habitats (Capblancq and Forester 2021). However, the results indicated that *SWK* is not included in the candidate adaptive outliers (supplementary fig. S7c, Supplementary Material online), most probably due to the limited number of absent SWK accessions. In summary, we hypothesize that the emergence of *SWK* may have enhanced Arabidopsis's adaptation to drought stress, although further evidence is required to support this conclusion.

To infer whether *SWK* is under positive selection in the natural populations, we performed a selection scan for each natural population, and found a total of 171 (21.87%) de novo genes are located at the selected regions in at least one population (Fig. 4e). Among them, the African population has the fewest de novo genes under selection (13, 1.66%), the northern Swedish population has the most de novo genes under selection (52, 6.65%) (Fig. 4e). The proportion of selected genes unique to each population ranges from 0 (African and Western European populations) to 50% (Yangtze River population) (Fig. 4e). Selection analysis based on the sequence polymorphic level suggested that *SWK* is not under positive selection in any population even global population (Fig. 4f and supplementary figs. S8 and S9, Supplementary Material online). Taken together, our results support that 21.87% de novo genes are under natural selection either directly or indirectly by hitchhiking effect in natural populations and *SWK* is not under positive selection.

## Discussion

De novo genes are important fuel for evolution (Levine et al. 2006; Cardoso-Moreira and Long 2012; Carvunis et al. 2012). Characterizing their function is the most important aspect to understand the origin of de novo genes. The functions of de novo genes that have been reported are mainly concentrated in 4 biological processes: reproduction, stress resistance, toxicity, and metabolic regulation (Singh and Syrkin Wurtele 2020). Among the 782 de novo genes in Arabidopsis (Li et al. 2016), only *QQS* has been functionally characterized (Li et al. 2009, 2015; Jiang et al. 2022). A previous study has shown that approximately half of the randomly selected gene mutants could affect Arabidopsis flowering time (Chong and Stinchcombe 2019). In contrast, here in our study, only 1 out of 7 de novo gene mutants showed a phenotypic effect under osmotic stress. This emphasizes that phenotypic effects of de novo genes only show up in very few specific develop stages or environmental conditions.

Here, we studied the function of *SWK* and revealed its role in response to osmotic stress and folate biosynthesis, which is consistent with the evolutionary characteristics of de novo genes that initially perform only regulatory functions (Capra et al. 2010; Zhang et al. 2015). The 71 genes that significantly altered *SWK* expression are significantly enriched in pathways of circadian rhythm-plant, plant hormone signal transduction, lysine degradation, phosphonate, and phosphinate metabolism based on DEGs between the wild type and mutant of *SWK* (supplementary fig. S4f, Supplementary Material online, supplementary table S4, Supplementary Material online), which implies that *SWK* responds to environmental, developmental signals.

De novo genes are one of the important sources of new genes besides gene duplication (Levine et al. 2006; Carvunis et al. 2012). The most intriguing question is how a de novo gene is maintained and integrates into existing regulatory pathways. For those new genes maintained in populations, certain biological or evolutionary functions are expected (He and Zhang 2005; Long et al. 2013; Kuzmin et al. 2022). It has been demonstrated that duplicate genes are easier to be integrated into gene networks than de novo genes of the same age, but they do not gain function as quickly as de novo genes (Capra et al. 2010). *cytHPPK/DHPS* is a young duplicate gene unique to Arabidopsis (Storozhenko et al. 2007). Our findings illustrate the phenomenon where a de novo gene could collaborate with a recently duplicated gene to integrate into 2 distinct pathways, the osmotic stress regulatory pathway and the folate biosynthesis pathway. In particular, our study illustrates that the origins of new genes through duplication and de novo origin are not independent events, but rather could be a mutually reinforcing process of genetic innovation. This study provides a new perspective on adding a de novo gene to existing gene regulatory networks.

In this study, we found that *SWK* can enhance the resistance of Arabidopsis to osmotic stress caused by mannitol, which is the main way to simulate drought stress (Zhu 2002; Verslues et al. 2006; Ozturk et al. 2021). Therefore, when we observe that the precipitation in the driest quarter in the distribution area of the *SWK*-present natural accessions is significantly lower than that of the *SWK*-absent natural accessions in the African population, we speculated that a de novo gene may improve the adaptability of Arabidopsis natural populations to drought environments. However, we did not detect any signal of natural selection for *SWK*, even though 21.87% of de novo genes were under natural selection in either one or a few populations. It is possible that *SWK* has been nearly fixed in globally distributed populations, and the signal of selection could not be detected any more (McLysaght and Hurst 2016). In addition, it is possible that it fixed directly after emergence during the speciation process without going through natural selection. However, in the Cape Verde Islands population, *SWK* was lost in 99.1% (332/335) accessions (supplementary fig. S10, Supplementary Material online).

Apparently, the exact mechanism of *SWK* fixation remains unclear, and further evidence is needed to better understand the evolutionary dynamics of de novo genes. In addition, the functional analysis of *SWK* in-depth will be insightful for revealing its function, and also will provide the broader implications of the findings for plant breeding, particularly in the context of climate change and the need for drought-resistant crops. Overall, this study highlights how a de novo gene originated and integrated into the existing pathways to regulate phenotypic variation and affect adaptation to environmental challenges at the population level.

## Materials and Methods

### Plant Material and Growth Condition

All Arabidopsis plants used in this study were Col-0 ecotype. The following mutants were used, including Salk_145188 (AT1G03106), Salk_037192C (AT1G58150), Salk_118656C (AT2G07000), Salk_024197C (AT2G13550), Salk_010561C (AT2G14460), Salk_089713C (AT4G36515), Salk_131289C (AT5G67245), Salk_093782 (AT1G69190), and Salk_076763C (AT5G41480). These T-DNA insertion mutants were obtained from the Arabidopsis Biological Resource Center (ABRC, http://abrc.osu.edu/).

Seeds were stratified at 4 °C for 3 d in 0.1% agar after surface-sterilized by 75% ethanol, and sowed into the soil. Plants were grown at 20 °C under long-day conditions (16-h day/8-h night).

### Characterization and Frequency Identification of de novo Genes

The presence of de novo genes in natural accessions was determined by the absence of loss-of-function mutations in these genes (Xu et al. 2019; Zhang et al. 2019a). Gene length, exon number, CDS length and GC content are calculated based on Col-0 (TAIR10); meanwhile, the number of transcripts, nucleotide polymorphism (π), DNA methylation level, the ratio of nonsynonymous to synonymous nucleotide diversity (π_n_/π_s_), codon usage bias, and the density of accessible chromatin regions are downloaded from previous studies (Hamala and Tiffin 2020).

### Vector Construction and Plant Transformation

In order to get the transgenic overexpressing plants, the full length coding sequence without stop codon of 7 de novo genes were cloned into the modified pCAMBIA 2300 vector, with a GFP in the C terminal, respectively. To generate *SWK*-knockout mutants, 4 guide RNAs targeting the exon and the 3′UTR of *SWK* (supplementary fig. S2c, Supplementary Material online) were designed and constructed into *pYLCRISPR–Cas9Pubi-H* according to the user's manual (Ma et al. 2015). The constructs were then transformed into *Agrobacterium tumefaciens* GV3101 and subsequently introduced into Col-0 plants by using the floral dip method (Clough and Bent 1998). The primers used for plasmid construction are listed in supplementary table S9, Supplementary Material online.

### Expression Profiles and Protein Structure Prediction of de novo Genes

The RNA-seq data of 6 tissues were downloaded from NCBI: root (accession number GSE116553) (Deforges et al. 2019), seedling (GSE98045) (Fukudome et al. 2017), stem (GSE121407) (Zhang et al. 2019b), leaf (GSE85653) (Albihlal et al. 2018), flower (GSE106943) (Yan et al. 2018), silique (GSE156491) (Li et al. 2021). In addition, the RNA-seq of seed (GSA: CRA014531) was completed in this study. RNA-seq data of 7 different tissues were normalized to TPM by StringTie (Pertea et al. 2015). Protein structure prediction was performed using the online tool AlphaFold2 (https://www.alphafold.ebi.ac.uk/) (Jumper et al. 2021).

### Measurement of Phenotypes and Hydrogen Peroxide Content

We counted the number of rosette leaves, with 10 replicates per sample (*n* = 10), on the 12th and 18th days after growth, respectively (Li et al. 1998). The fresh weight (*n* = 3) (El-Lithy et al. 2004) and chlorophyll content of rosette leaves (*n* = 3) were measured on the 19th day after growth. Chlorophyll content was assessed using acetone extraction (Arnon 1949). On the 20th day after growth, we noted the time when the first flower bud emerged (*n* = 10) (Atwell et al. 2010). The length of siliques (for the first 10 siliques per plant, *n* = 5) was measured on the 49th day after growth (Atwell et al. 2010). On the 60th day after sowing, the number of secondary branches (*n* = 10) and plant height (*n* = 10) were recorded (Boyes et al. 2001). The schedule for these phenotypic measurements was aligned with the developmental stages of Arabidopsis (Boyes et al. 2001).

The experiment to count the seed germination was conducted on Petri dishes. The experimental procedures and climate room conditions were consistent with previous studies (Wang et al. 2016), and the seeds were not stratified as described here, nor did we use 75% alcohol to treat the seeds. The treatment solution included: water, 150 or 200 mM NaCl solution, 300 or 400 mM mannitol solution. Each germination experiment was performed with 6 replicates. Radicle outgrowth is defined as the criterion for judging germination (Alonso-Blanco et al. 2003).

The root length experiment was carried out in a square Petri dish with a side length of 13 cm. Four-day-old seedlings grown on MS plates were transferred to new MS plates or those supplemented with 100 or 150 mM NaCl, 200 or 300 mM mannitol, and 0.01 or 0.05 μM paraquat, ensuring that the root tips were aligned at the same level. Each germination experiment was performed with 15 replicates. After culturing the root tip of the seedlings vertically downward for 7 d (20 °C, 16-h day/8-h night), the length of the root growth was measured using a ruler (Verslues et al. 2006).

The hydrogen peroxide content was determined using a hydrogen peroxide quantitative test kit (Boxbio; AKAO009C) according to the manufacturer's instructions (Qiu et al. 2022).

### Subcellular Localization and Yeast Two-Hybrid Assay

To examine the subcellular localization of SWK, we used *35S::SWK-GFP* and *35S::GFP* seedlings to observe. Root tips and leaves of 4-day-old seedlings were observed under a super-resolution confocal microscope (Zeiss LSM 980 with Elyra7). GFP fluorescence was detected at 488 nm (excitation) and 509 nm (emission). Nuclei were labeled with DAPI staining. DAPI staining fluorescence was detected at 353 nm (excitation) and 465 nm (emission). Autofluorescence of the chloroplasts was detected at 650 to 750 nm. The yeast two-hybrid assay was carried out based with the GAL4-based two-hybrid system according to the manufacturer's instructions (Clontech, USA) (Li et al. 2023).

### Expression Analysis

We used RNAprep Pure Plant Plus Kit (Cat.#DP441, TIANGEN) to extract total RNA from dry seeds or imbibing seeds for 12 h under light, and Micro Elute total Plant RNA Kit (Cat.#R6831-01, Omega) to extract total RNA from other plant materials. We reverse-transcribed 1 μg of RNA into cDNA using the RevertAid First Strand cDNA Synthesis Kit (Cat.#K1622, Thermo Scientific). We conducted RT-qPCR using TB Green Premix Ex Taq (Cat. #RR420A, TaKaRa) on Analytik Jena AG (Jena). Each RT-qPCR experiment included 3 biological replicates and 3 technical replicates per sample. Expression levels were calculated using the 2^−ΔΔCT^ method and normalized to *Actin8* (AT1G49240) (Livak and Schmittgen 2001). The primers used for the gene expression analyses are listed in supplementary table S9, Supplementary Material online.

### eGWAS Analysis

To identify potential causal variants associated with *SWK* gene expression variation across natural accessions, we performed GWAS using EMMAX (Kang et al. 2010) and GEMMA (Zhou and Stephens 2012), both based on the linear mixed model, with the expression levels of *SWK* from leaves of 414 natural accessions (Kawakatsu et al. 2016). A total of 1,133,651 SNPs, with a missing rate of less than 10% and a MAF >5% were used as genotype. Principal components and a kinship matrix were used to control for population structure. The significant threshold was set based on Bonferroni correction [−log_10_ (0.05/1,133,651)].

### High-throughput RNA Sequencing and Differential Expression Analysis

Total RNA was extracted from the control and 400 mM mannitol-stressed imbibing seeds after 12 h of light exposure. Three biological replicates were utilized for each sample. A sequencing library was constructed for paired-end 150 bp read length sequencing on the Illumina HiSeq 2500 platform. Sequence alignment was performed using TopHat v2.0.12 (Kim et al. 2013). The reads were counted by HTSeq (Anders et al. 2015). Subsequently, StringTie v 2.2.0 assembled reads into transcripts and calculated TPM values for each gene (Pertea et al. 2015). DESeq2 v1.18.1 conducted statistical analysis of DEGs (Anders and Huber 2010). Given the de novo genes originated recently and their interaction with other genes might be weak, we used the value of log_2_FoldChange >0.2 and FDR <0.05 for differential expression gene analysis. We verified the identified candidate downstream genes by RT-qPCR.

### KEGG Pathway Analysis and Evolutionary Analysis

In this study, we used online KOBAS v3.0 (http://39.103.204.200/genelist/) to perform KEGG pathway analysis (Bu et al. 2021). The default set of genes used to test for enrichment was all annotated genes in the *A. thaliana* genome (TAIR10).

We performed blastn analysis using several databases: Phytozome 13 (304 plant genomes) (Goodstein et al. 2012), MycoCosm (2,418 fungus genomes) (Grigoriev et al. 2014), PhycoCosm (178 algae genomes) (Grigoriev et al. 2021), IMG/M (12,387 microorganism genomes) (Chen et al. 2023), and Ensembl 109 (369 animal genomes) (Cunningham et al. 2022). For all blastn searches, we used a stringent E-value threshold of 1e-5 to ensure that only highly significant hits were considered. The analysis was performed using the default parameters provided by the blastn online genome databases, with further manual curation to remove redundant or low-quality hits. We filtered the results based on sequence identity and alignment length, ensuring that only sequences with a minimum identity of 60% and alignment covering at least 70% of the query sequence were retained for further analysis.

MEGA X was employed for alignment and phylogenetic analysis (Kumar et al. 2018). All nucleotide sequences were aligned to the Col-0 reference genome, and amino acids were translated according to the Col-0 reference reading frame (note that this implies frameshifts in individual sequences are not represented). All compilation types were determined based on the comparison. Ancestral sequence reconstruction was performed using FastML with default parameters (Ashkenazy et al. 2012). MCScanX, implemented in TBtools, was utilized for syntenic analysis (Chen et al. 2020). The genome *Aethionema arabicum* was downloaded from PLAZA (https://bioinformatics.psb.ugent.be/plaza/), while all other genome data were obtained from Phytozome (https://phytozome-next.jgi.doe.gov/). TSD was searched for using the online tool CENSOR (https://www.girinst.org/censor/index.php) (Kohany et al. 2006). The sequencing data for the accessions of the Cape Verde Islands were obtained from the study by Fulgione et al. (2022).

Nineteen climate factors were derived from the WORLDCLIM database (www.worldclim.org). The comparison of environmental factor differences in the natural geographic distribution of *SWK* presence or absence in African populations was conducted using the two-sided Student's *t*-test. The RDA analysis was performed using previously reported software (Capblancq and Forester 2021). Four environmental factors, including bio2 (mean diurnal range), bio4 (temperature seasonality), bio17 (Precipitation of driest quarter), and bio19 (precipitation of coldest quarter) were used after filtering high correlated factors (Pearson's correlation >0.7 were filtered).

The selective sweep regions were identified using OmegaPlus (version 3.0.3) (Alachiotis et al. 2012), where the ω statistic was computed at 10 kb intervals with the parameters “-minwin 10,000” and “-maxwin 100,000.” The top 5% regions with the high ω value were defined as regions under positive selection.

### Statistical Analyses

All the statistical analyses were performed in R (http://www.r-project.org/). Benjamini–Hochberg corrected *P*-values were applied for multiple testing.

## Supplementary Material

msae262_Supplementary_Data

## Data Availability

The raw sequence data reported in this paper have been deposited in the Genome Sequence Archive in National Genomics Data Center (CNCB-NGDC Members and Partners 2022), China National Center for Bioinformation/Beijing Institute of Genomics, Chinese Academy of Sciences (GSA: CRA014531) that are publicly accessible at https://ngdc.cncb.ac.cn/gsa. The data underlying this article are available in the article and in its online supplementary material.
