# Supplementary material for: A de novo Gene Promotes Seed Germination Under Drought Stress in Arabidopsis
Source: Mol Biol Evol. 2024 Dec 24;42(1):msae262. doi: 10.1093/molbev/msae262 (PMC11721784; doi:10.1093/molbev/msae262)
Supplement: msae262_Supplementary_Data [file msae262_supplementary_data.pdf]

Supplementary information for

A de novo gene promotes seed germination under drought stress in Arabidopsis

Guang-Teng Jin[1,2,3§], Yong-Chao Xu[1,2§], Xing-Hui Hou[1,2], Juan Jiang[1,2,3], Xin-Xin Li[1,2,3], Jia-Hui Xiao[1,2,3], Yu-Tao Bian[1,2,3], Yan-Bo Gong[1,2,3], Ming-Yu Wang[4], Zhi-Qin Zhang[1,2,3], Yong E. Zhang[3,5], Wang-Sheng Zhu[4], Yong-Xiu Liu[2,3,6], Ya-Long Guo[1,2,3*]

[1] State Key Laboratory of Systematic and Evolutionary Botany, Institute of Botany, Chinese Academy of Sciences, Beijing 100093, China

[2] China National Botanical Garden, Beijing 100093, China

[3] College of Life Sciences, University of Chinese Academy of Sciences, Beijing 100049, China

[4] Department of Plant Pathology, China Agricultural University, Beijing, 100193, China

[5] State Key Laboratory of Integrated Management of Pest Insects and Rodents & Key Laboratory of the Zoological Systematics and Evolution, Institute of Zoology, Chinese Academy of Sciences, Beijing 100101, China

[6] Key Laboratory of Plant Molecular Physiology, Institute of Botany, Chinese Academy of Sciences, Beijing 100093, China

§ These authors contributed equally to this work

* Corresponding author: Ya-Long Guo, yalong.guo@ibcas.ac.cn

Running title: A de novo gene promotes seed germination under drought stress

Key words: Adaptive evolution, *Arabidopsis thaliana*, de novo gene, regulatory networks

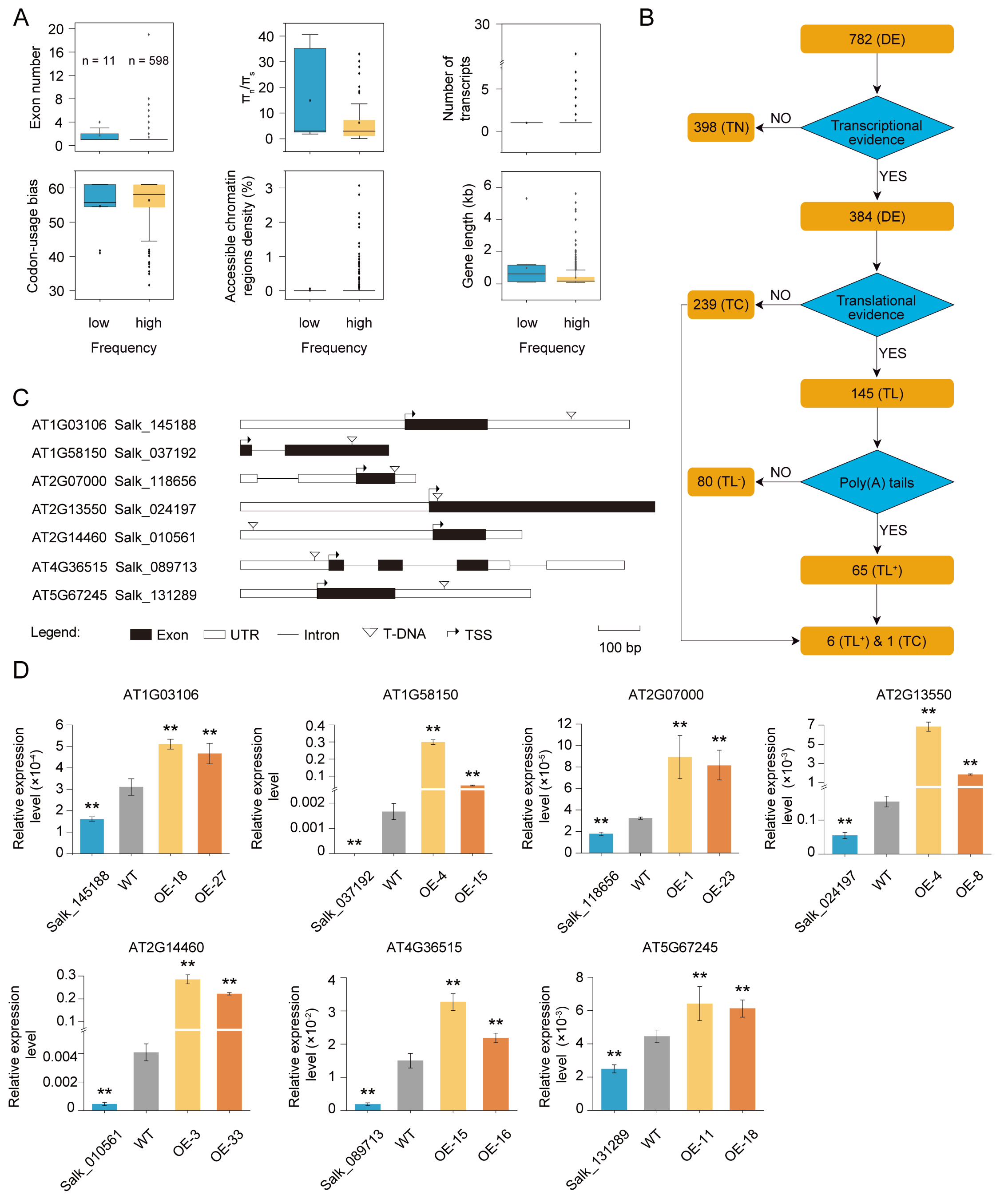

Figure S1. Characterization of de novo genes in Arabidopsis. A. Comparison of gene features between low-frequency (frequency < 10%) and high-frequency (frequency >= 90%) de novo genes in 1,115 accessions. π_n, non-synonymous nucleotide diversity; π_s, synonymous nucleotide diversity. Gene features were calculated based on the Col-0 reference. B. Screening process of representative de novo genes. DE, de novo genes; TN, de novo genes without expression evidence; TC, de novo genes with only transcriptional evidence; TL, de novo genes with

translational evidence; TL^-, TL genes without detected Poly(A) tails; TL^+, TL genes with detected Poly(A) tails. C. Mutant information of the 7 selected genes. D. RT-qPCR results of mutant, wild-type (WT) and overexpression (OE) lines of 7 selected genes. Data are means (± s.d.) of three replicates per line. RNA was extracted from seedlings. Either mutant or OE was compared to WT. Two-sided Student's *t*-test was used for significance test. **, $p < 0.01$.

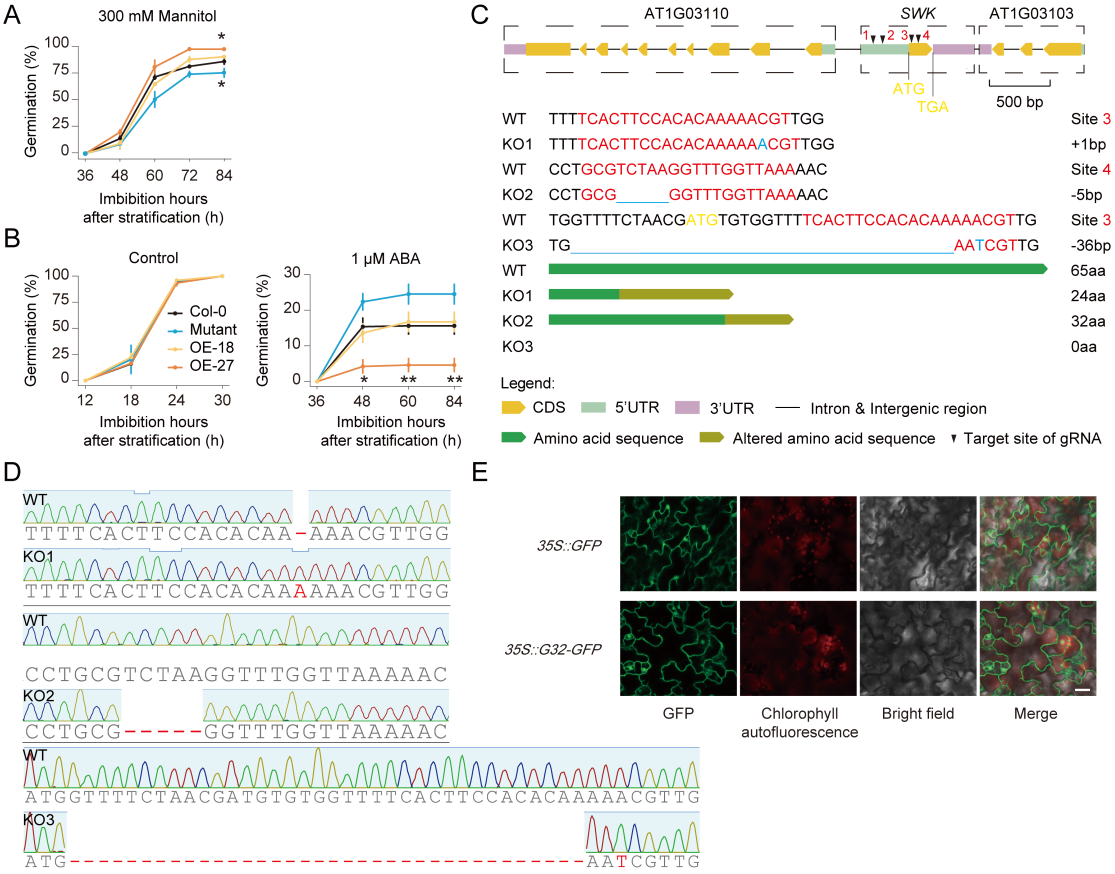

Figure S2. Function validation of *SWK*. A, B. Seed germination percentage under mannitol or ABA stress. Seed germination percentages are means (± s.d.) based on the seeds from six individual plants. Either mutant or OE was compared to WT at the same timepoint. Two-side Student's *t*-test. *, $p < 0.05$; **, $p < 0.01$. C. DNA sequence and predicted truncated proteins of knockout mutants (KOs) generated by CRISPR/Cas9. D. Sequencing results of knockout mutants (KOs) created by CRISPR/Cas9. E. Subcellular localization of SWK in the leaf. The images were obtained from the GFP channel, Chlorophyll autofluorescence channel, Bright channel and a merged image of the three channels. Scale bar, 20 μm.

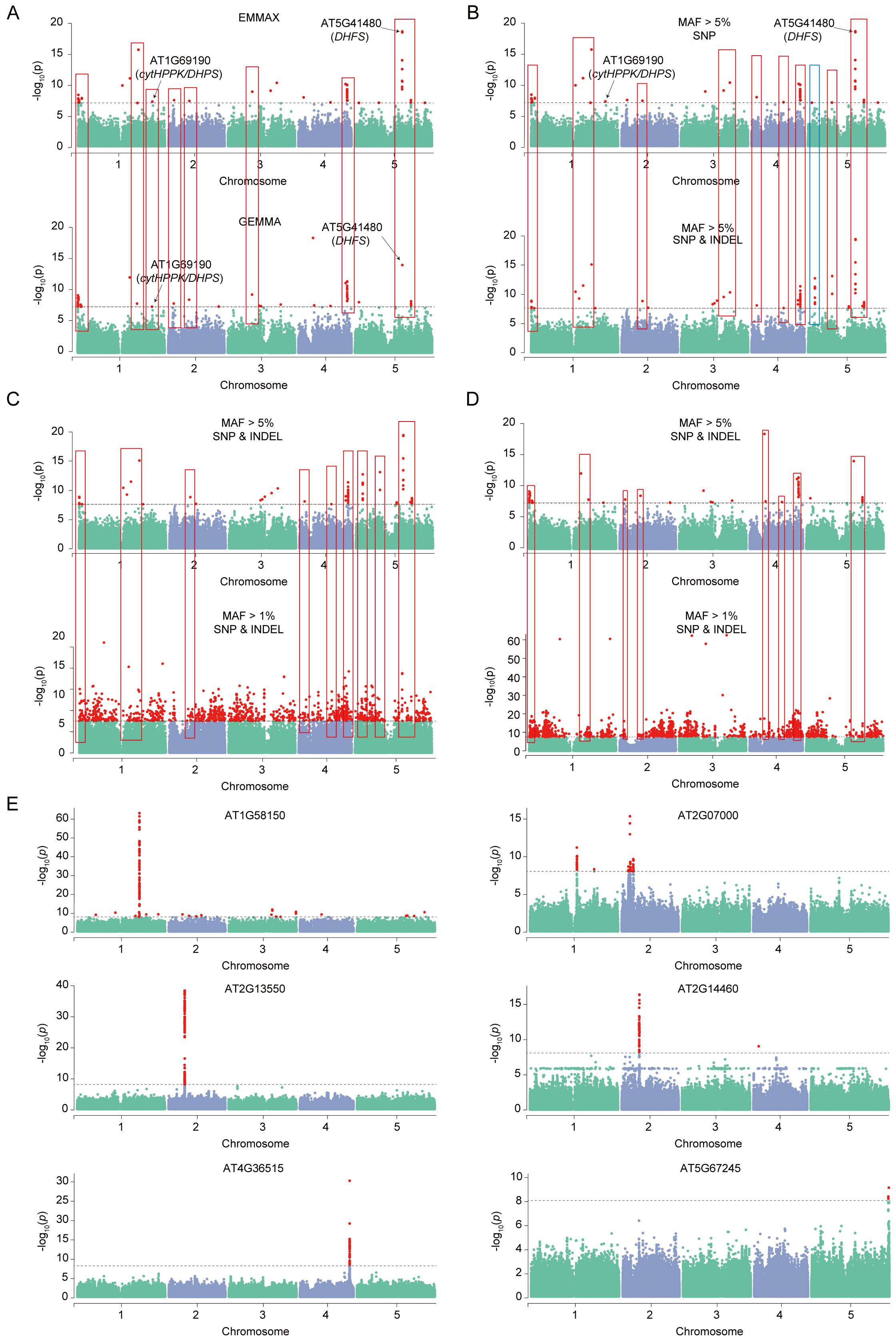

Figure S3. eGWAS analysis to identify upstream genes of de novo genes. A. Comparison of eGWAS results obtained from EMMAX and GEMMA

software (MAF > 5%). Shared peaks are highlighted with red boxes. B. Differences in eGWAS results between using only SNPs and using both SNPs and INDELs, using EMMAX software with MAF > 5%. Shared peaks are highlighted with red boxes, while different peaks are highlighted with blue boxes. C. Differences in eGWAS results when using SNPs and INDELs with MAF > 5% and MAF > 1%, using EMMAX software. Shared peaks are highlighted with red boxes. D. Differences in eGWAS results when using SNPs and INDELs with MAF > 5% and MAF > 1%, using GEMMA software. Shared peaks are highlighted with red boxes. E. eGWAS analysis based on the expression levels of six other de novo genes and whole-genome variation across 414 *A. thaliana* accessions. The dashed line is the threshold line [$-\log_{10}(0.05/1,133,651)$].

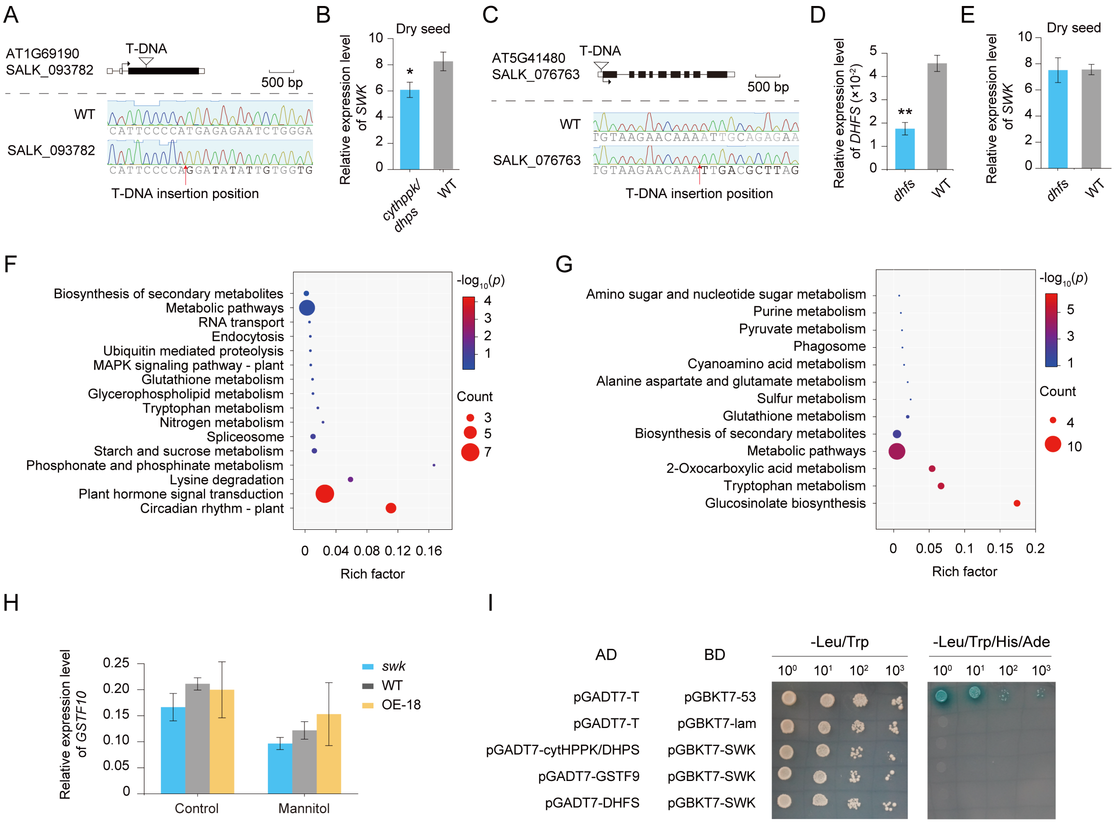

Figure S4. Identification of the upstream and downstream genes of *SWK*. A. Mutant structures of *cytHPPK/DHPS* (AT1G69190). The red arrow in the peak diagram represents the T-DNA insertion position. B. The expression difference of *SWK* in *cytHPPK/DHPS* mutant (SALK_093782) and WT in dry seed. Values are means (± s.d.) of three replicates per line. C. Mutant structures of *DHFS* (AT5G41480). The red arrow in the peak diagram represents the T-DNA insertion position. D. The expression difference of *DHFS* in *DHFS* mutant (SALK_076763) and WT. Values are means (± s.d.) of three replicates per line. E. The expression difference of *SWK* in *DHFS* mutant (SALK_076763) and WT in dry seeds. Values are means (± s.d.) of three replicates per line. F. KEGG enrichment analysis of *SWK* candidate upstream genes screened by mutant RNA-seq library. The expression of *SWK* changed more than 2-fold in mutants of these genes. G. KEGG enrichment analysis of the shared gene set of Figure 3E. H. Expression levels of the candidate downstream gene *GSTF10* in *SWK* mutant, WT and OE under mannitol stress. Values are means (± s.d.) of three replicates per line. I. Yeast two-hybrid of upstream and downstream

proteins and SWK. 10^0 – 10^3 is the dilution ratio of bacterial solution. Two-sided Student's *t*-test was used for significance test. *, $p < 0.05$; **, $p < 0.01$.

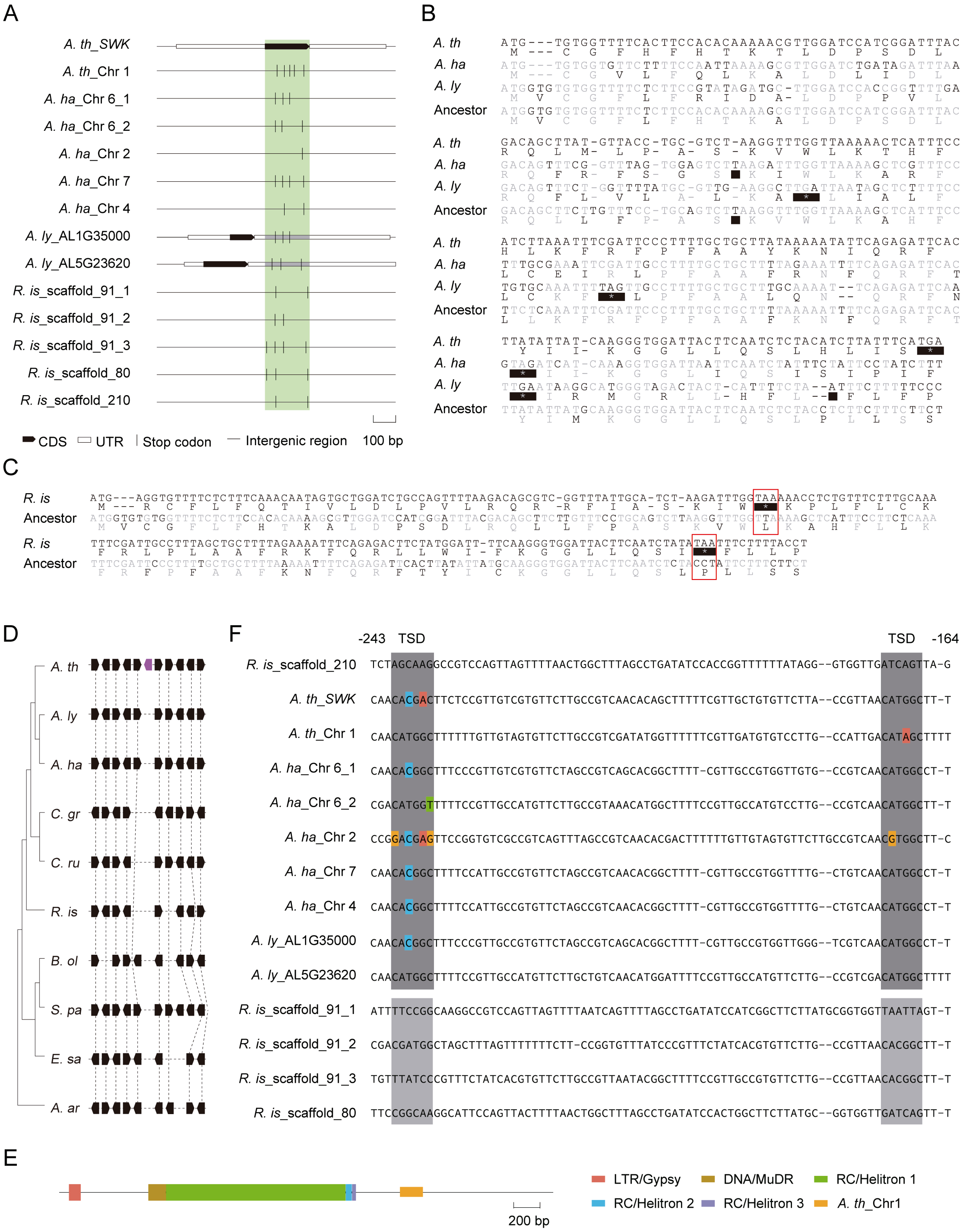

Figure S5. Evolutionary analysis of *SWK*. A. Homologous sequences of *SWK* in other species. The shaded area represents the homologous sequence of the *SWK* CDS. B. The alignment of the *SWK* gene from three species of the *Arabidopsis* genus and the inferred ancestral sequence based on this alignment. All nucleotide sequences were aligned to the *SWK* of Arabidopsis, and amino acids were translated according to the

reference frame (note that this implies frameshifts in individual sequences are not represented). C. Sequence alignment of inferred ancestral sequence of the *Arabidopsis* genus and the *R. islandica*. The red boxes highlight the two stop codons that are absent relative to *R. islandica*. Amino acids were translated according to *R. islandica* frame (note that this implies frameshifts in individual sequences are not represented). D. Synteny analysis was conducted among representative species of Brassicaceae. E. Transposable elements near the homologous sequence of *SWK* in Arabidopsis were identified. F. Relics of transposable elements insertions upstream of the *SWK* start codon. The dark shaded area represents the observed TSD, and the light shaded area represents the corresponding sequence where no TSD was observed. Colored shading indicates different types of nucleotide variants. *A. th* represents *Arabidopsis thaliana*, *A. ly* represents *Arabidopsis lyrate*, *A. ha* represents *Arabidopsis helleri*, *C. gr* represents *Capsella grandiflora*, *C. ru* represents *Capsella rubella*, *R. is* represents *Rorippa islandica*, *B. ol* represents *Brassica oleracea*, *S. pa* represents *Schrenkiella parvula*, *E. sa* represents *Eutrema salsugineum*, *A. ar* represents *Aethionema arabicum*.

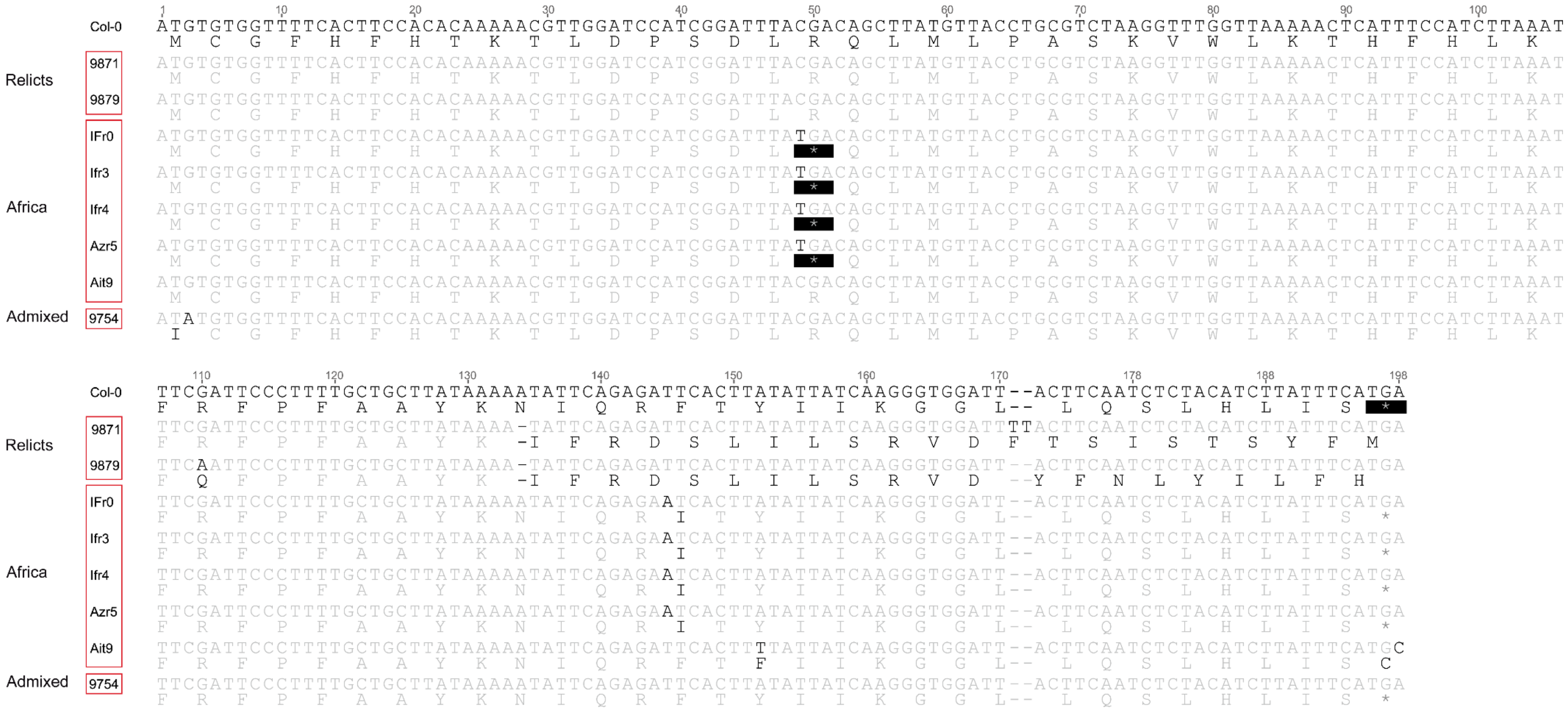

Figure S6. Sequence variation in eight natural accession that did not form *SWK*. The natural accessions within the red box represent those from different populations.

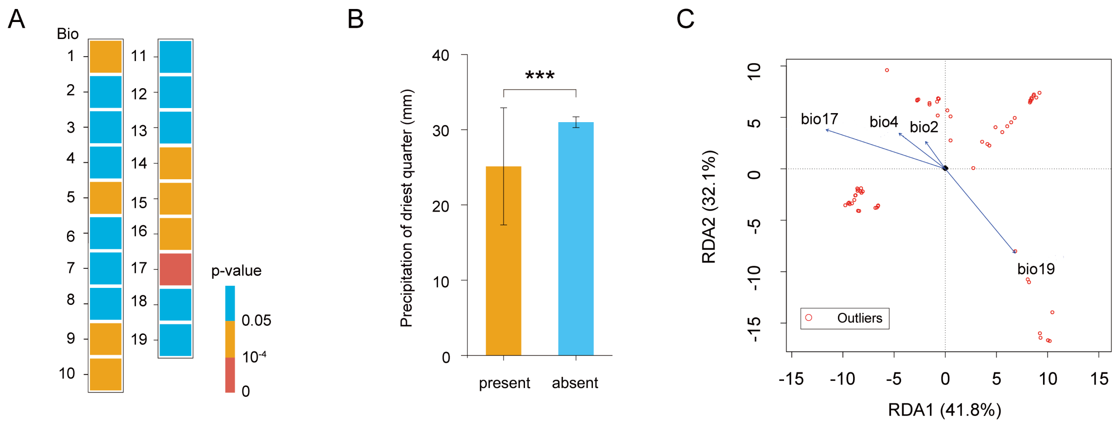

Figure S7. The role of *SWK* presence in the environmental adaptation of Arabidopsis. A. The *p*-values of the two-sided Student's *t*-test for 19 climate factors in the distribution areas of the *SWK*-present accessions (n = 55) and the *SWK*-absent accessions (n = 5). B. Comparison of bio17 (precipitation of the driest quarter) distributions between *SWK*-present accessions (n = 55) and *SWK*-absent accessions (n = 5). Two-sided Student's *t*-test, ***, $p < 0.001$. C. RDA analysis of African population with environmental factors. The projection of loci and environmental variables along the first two RDA axes. The locus scores are rescaled to an unshown axis for better visibility.

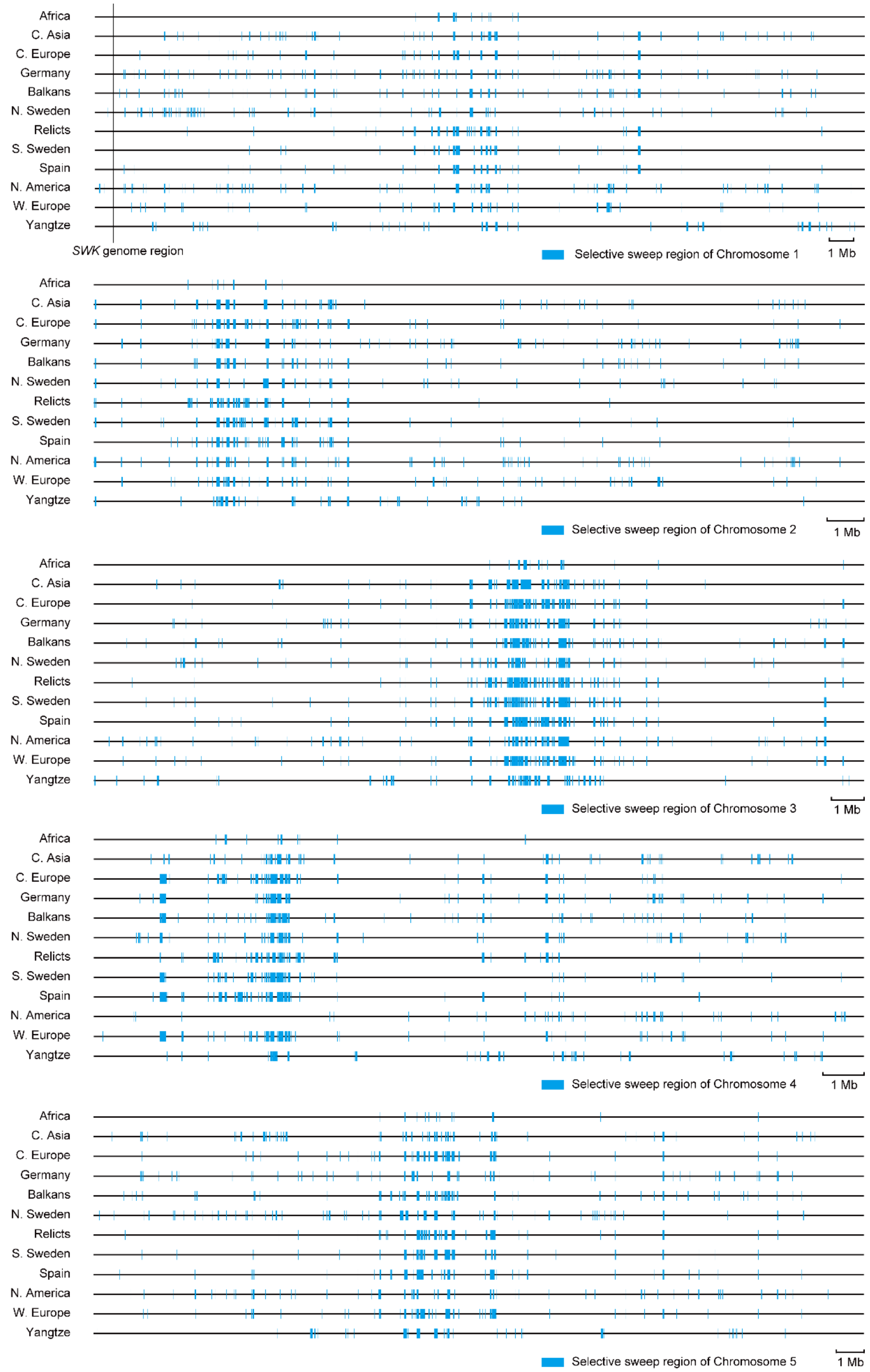

Figure S8. Selective sweep regions of chromosome 1-5 in 12 natural populations.

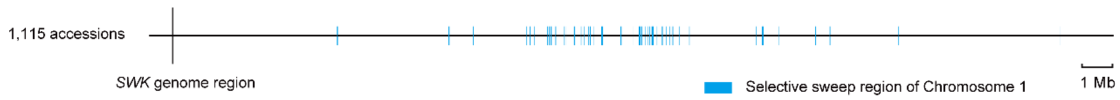

Figure S9. Selective sweep regions on chromosome 1 in all 1,115 accessions.

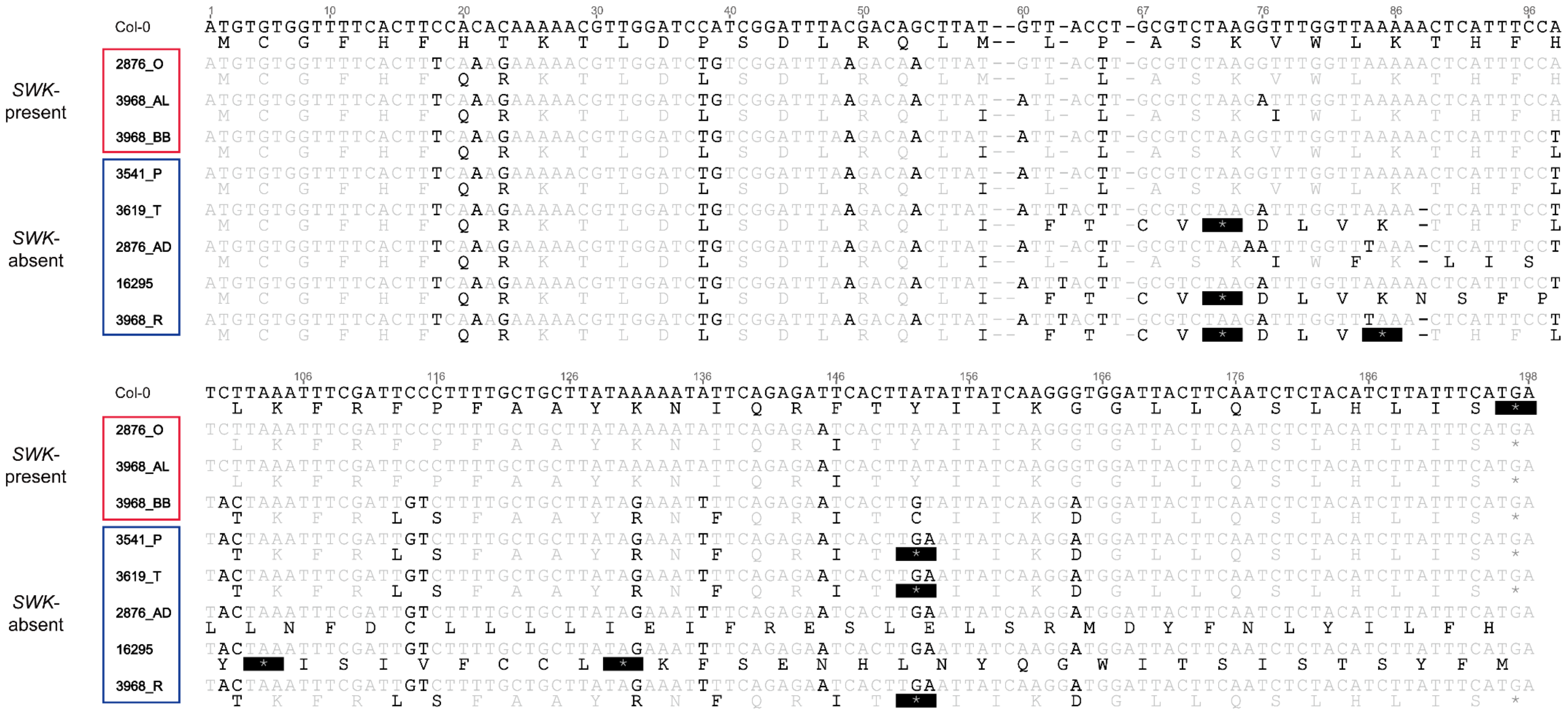

Figure S10. *SWK* sequences in the Cape Verde Islands population. The sequences of three *SWK*-present natural accessions and the major sequence variants in other *SWK*-absent natural accessions.

Supplementary Table 1. eGWAS significance signals of seven de novo genes.

Chrom-osome	Location	Bonferroni correction *p*-value	eGWAS significance signal of genes	Chrom-osome	Location	Bonferroni correction *p*-value	eGWAS significance signal of genes
chr1	93221	1.26E-09	AT1G03106	chr2	5612531	1.35E-09	AT2G13550
chr1	93884	1.84E-09	AT1G03106	chr2	5614472	8.28E-28	AT2G13550
chr1	95301	2.65E-10	AT1G03106	chr2	5614903	8.83E-10	AT2G13550
chr1	95749	2.66E-10	AT1G03106	chr2	5615194	2.61E-11	AT2G13550
chr1	96303	6.93E-09	AT1G03106	chr2	5615689	8.72E-12	AT2G13550
chr1	96726	1.8E-09	AT1G03106	chr2	5616485	4.81E-29	AT2G13550
chr1	353868	5.59E-09	AT1G03106	chr2	5617615	7.1E-31	AT2G13550
chr1	353951	6.55E-09	AT1G03106	chr2	5617791	2.92E-10	AT2G13550
chr1	353957	6.18E-09	AT1G03106	chr2	5618270	5.15E-10	AT2G13550
chr1	353967	2.93E-09	AT1G03106	chr2	5618640	7.59E-30	AT2G13550
chr1	1305357	1.33E-09	AT1G03106	chr2	5618828	8.34E-11	AT2G13550
chr1	1305366	1.4E-09	AT1G03106	chr2	5620883	3.75E-27	AT2G13550
chr1	1305505	1.08E-09	AT1G03106	chr2	5621441	4.03E-09	AT2G13550
chr1	1305563	1.27E-09	AT1G03106	chr2	5621645	1.63E-26	AT2G13550
chr1	15512040	4.72E-12	AT1G03106	chr2	5621951	6.47E-11	AT2G13550
chr1	18171031	2.18E-13	AT1G03106	chr2	5622423	2.25E-10	AT2G13550
chr1	20778294	8.45E-09	AT1G03106	chr2	5622547	4.65E-10	AT2G13550
chr1	21135306	9.85E-19	AT1G03106	chr2	5622711	5.28E-10	AT2G13550
chr1	26009867	4.86E-09	AT1G03106	chr2	5623272	2.63E-10	AT2G13550
chr2	1991745	2.59E-09	AT1G03106	chr2	5623502	4.36E-10	AT2G13550
chr2	7363617	3.65E-09	AT1G03106	chr2	5623704	3.08E-10	AT2G13550
chr3	8484379	6.73E-11	AT1G03106	chr2	5624036	1.62E-28	AT2G13550
chr3	14919433	4.34E-11	AT1G03106	chr2	5624121	8.78E-11	AT2G13550
chr3	17110006	1.51E-12	AT1G03106	chr2	5625361	1.2E-29	AT2G13550
chr4	1825844	7.72E-10	AT1G03106	chr2	5625381	6.2E-10	AT2G13550
chr4	11230951	6.76E-09	AT1G03106	chr2	5625470	3.19E-10	AT2G13550
chr4	16520335	2.54E-12	AT1G03106	chr2	5625510	2.43E-10	AT2G13550
chr4	17052238	4.63E-11	AT1G03106	chr2	5625911	2.38E-10	AT2G13550
chr4	17052742	7.32E-11	AT1G03106	chr2	5625923	4.58E-10	AT2G13550
chr4	17052889	1.77E-10	AT1G03106	chr2	5626165	2.02E-29	AT2G13550
chr4	17052909	5.23E-11	AT1G03106	chr2	5626296	9.21E-30	AT2G13550
chr4	17052916	5.42E-11	AT1G03106	chr2	5626766	5.66E-10	AT2G13550
chr4	17052951	5.43E-11	AT1G03106	chr2	5627117	6.15E-10	AT2G13550
chr4	17052958	4.86E-11	AT1G03106	chr2	5627718	1.65E-26	AT2G13550
chr4	17053066	2.45E-11	AT1G03106	chr2	5627960	1.01E-28	AT2G13550
chr4	17053096	4.41E-11	AT1G03106	chr2	5628143	6.6E-09	AT2G13550

chr4	17053272	3.42E-10	AT1G03106	chr2	5628353	1.14E-28	AT2G13550
chr4	17053320	1.72E-10	AT1G03106	chr2	5628706	8.87E-31	AT2G13550
chr4	17053916	3.5E-12	AT1G03106	chr2	5628824	9.37E-33	AT2G13550
chr4	17054020	5.49E-10	AT1G03106	chr2	5628927	4.98E-10	AT2G13550
chr4	17054031	5E-10	AT1G03106	chr2	5629265	9.33E-10	AT2G13550
chr4	17054215	4.3E-11	AT1G03106	chr2	5629277	1.03E-28	AT2G13550
chr4	17054217	1.23E-10	AT1G03106	chr2	5629519	3.22E-10	AT2G13550
chr4	17054344	5.87E-10	AT1G03106	chr2	5629544	3.54E-29	AT2G13550
chr4	17054346	6.03E-10	AT1G03106	chr2	5629914	4.62E-27	AT2G13550
chr4	17054564	2.51E-09	AT1G03106	chr2	5629965	8.32E-10	AT2G13550
chr4	17054567	3.15E-09	AT1G03106	chr2	5630519	2.73E-24	AT2G13550
chr4	17054584	6.63E-10	AT1G03106	chr2	5630535	6.67E-10	AT2G13550
chr4	17054624	8.59E-10	AT1G03106	chr2	5630957	2.11E-10	AT2G13550
chr4	17054874	4E-12	AT1G03106	chr2	5631105	6.74E-26	AT2G13550
chr4	17227784	2.85E-09	AT1G03106	chr2	5631188	1.43E-10	AT2G13550
chr5	16599736	3.82E-22	AT1G03106	chr2	5631271	5.96E-26	AT2G13550
chr5	16598321	4.99E-22	AT1G03106	chr2	5636582	1.01E-10	AT2G13550
chr5	16605481	5.56E-22	AT1G03106	chr2	5636693	5.1E-10	AT2G13550
chr5	16568093	9.11E-17	AT1G03106	chr2	5637649	1.03E-32	AT2G13550
chr5	16579881	4.15E-15	AT1G03106	chr2	5637656	5.84E-11	AT2G13550
chr5	16549761	6.67E-13	AT1G03106	chr2	5637717	6.41E-32	AT2G13550
chr5	16611366	8.43E-13	AT1G03106	chr2	5638578	1.54E-34	AT2G13550
chr5	16549764	8.29E-12	AT1G03106	chr2	5638947	6.76E-34	AT2G13550
chr5	16557831	2.32E-11	AT1G03106	chr2	5639240	3.2E-34	AT2G13550
chr5	19617976	2.52E-09	AT1G03106	chr2	5639441	3.22E-14	AT2G13550
chr5	19618529	6.12E-09	AT1G03106	chr2	5639691	2.34E-34	AT2G13550
chr5	19618073	6.56E-09	AT1G03106	chr2	5640349	3.61E-09	AT2G13550
chr5	8438798	7.73E-09	AT1G03106	chr2	5640723	2.16E-34	AT2G13550
chr5	24497791	8.04E-09	AT1G03106	chr2	5641000	7.52E-09	AT2G13550
chr5	1402813	8.25E-09	AT1G03106	chr2	5641915	2.73E-35	AT2G13550
chr1	6403196	6.51E-10	AT1G58150	chr2	5641945	7.42E-31	AT2G13550
chr1	13171410	3.77E-11	AT1G58150	chr2	5642341	7.25E-13	AT2G13550
chr1	20144855	2.65E-09	AT1G58150	chr2	5642365	3.59E-37	AT2G13550
chr1	21398721	2.03E-11	AT1G58150	chr2	5642397	6.37E-36	AT2G13550
chr1	21400918	8E-09	AT1G58150	chr2	5642403	2.78E-36	AT2G13550
chr1	21418182	5.53E-09	AT1G58150	chr2	5642636	1.22E-38	AT2G13550
chr1	21516733	8.47E-19	AT1G58150	chr2	5642639	1.12E-38	AT2G13550
chr1	21519399	1.8E-10	AT1G58150	chr2	5642665	2.11E-38	AT2G13550
chr1	21519568	1.29E-29	AT1G58150	chr2	5642678	2.02E-38	AT2G13550
chr1	21519591	5.45E-29	AT1G58150	chr2	5642721	9.85E-13	AT2G13550
chr1	21519727	6.43E-25	AT1G58150	chr2	5642726	1.49E-39	AT2G13550
chr1	21519738	1.23E-22	AT1G58150	chr2	5642735	2.32E-39	AT2G13550
chr1	21519757	2.09E-26	AT1G58150	chr2	5642738	4.15E-39	AT2G13550

chr1	21519799	4.22E-26	AT1G58150	chr2	5642877	3.91E-39	AT2G13550
chr1	21519810	5.46E-27	AT1G58150	chr2	5642901	5.23E-34	AT2G13550
chr1	21519881	7.53E-35	AT1G58150	chr2	5642930	5.4E-31	AT2G13550
chr1	21520069	1.45E-38	AT1G58150	chr2	5642975	2.17E-35	AT2G13550
chr1	21520096	1.26E-37	AT1G58150	chr2	5642981	2.04E-33	AT2G13550
chr1	21520184	7.06E-40	AT1G58150	chr2	5643184	7.69E-34	AT2G13550
chr1	21520186	1.01E-38	AT1G58150	chr2	5643247	3.57E-36	AT2G13550
chr1	21520273	4.96E-35	AT1G58150	chr2	5643444	5.14E-33	AT2G13550
chr1	21520288	8.02E-35	AT1G58150	chr2	5643544	3.17E-33	AT2G13550
chr1	21520297	1.85E-36	AT1G58150	chr2	5643585	3.01E-14	AT2G13550
chr1	21520301	6.99E-33	AT1G58150	chr2	5643620	6.45E-34	AT2G13550
chr1	21520312	3.32E-36	AT1G58150	chr2	5643759	4.87E-38	AT2G13550
chr1	21520399	2.75E-27	AT1G58150	chr2	5643802	1.68E-34	AT2G13550
chr1	21520530	2.11E-45	AT1G58150	chr2	5643836	3.26E-34	AT2G13550
chr1	21520569	2.57E-45	AT1G58150	chr2	5643859	1.2E-33	AT2G13550
chr1	21521053	2.19E-46	AT1G58150	chr2	5649041	2.13E-30	AT2G13550
chr1	21521087	2.86E-46	AT1G58150	chr2	5649058	8.97E-31	AT2G13550
chr1	21521088	1.98E-46	AT1G58150	chr2	5649073	2.76E-27	AT2G13550
chr1	21521165	1.13E-61	AT1G58150	chr2	5649084	8.42E-36	AT2G13550
chr1	21521177	1.8E-49	AT1G58150	chr2	5649133	3.26E-35	AT2G13550
chr1	21521186	1.67E-49	AT1G58150	chr2	5649160	4.62E-35	AT2G13550
chr1	21521276	1.19E-32	AT1G58150	chr2	5649232	2.63E-28	AT2G13550
chr1	21521438	7.91E-49	AT1G58150	chr2	5649236	3.79E-14	AT2G13550
chr1	21521474	9.26E-51	AT1G58150	chr2	5649246	8.25E-26	AT2G13550
chr1	21521501	1.18E-50	AT1G58150	chr2	5649259	1.1E-11	AT2G13550
chr1	21521558	1.53E-48	AT1G58150	chr2	5649274	1.17E-24	AT2G13550
chr1	21521579	2.85E-50	AT1G58150	chr2	5650547	5.97E-09	AT2G13550
chr1	21521582	5.77E-49	AT1G58150	chr2	5650583	1.01E-09	AT2G13550
chr1	21521744	4.97E-21	AT1G58150	chr2	5650699	5.06E-09	AT2G13550
chr1	21521777	1.2E-21	AT1G58150	chr2	5650709	3.08E-09	AT2G13550
chr1	21521816	2.61E-22	AT1G58150	chr2	5650924	4.75E-09	AT2G13550
chr1	21521831	1.7E-21	AT1G58150	chr2	5651002	7.31E-09	AT2G13550
chr1	21521970	1.77E-50	AT1G58150	chr2	5651176	9.7E-10	AT2G13550
chr1	21522029	1.57E-64	AT1G58150	chr2	5651213	2.97E-09	AT2G13550
chr1	21522140	2.48E-62	AT1G58150	chr2	5651243	2.26E-09	AT2G13550
chr1	21522142	5.24E-45	AT1G58150	chr2	5651314	5.08E-09	AT2G13550
chr1	21522259	3.24E-44	AT1G58150	chr2	5651341	1.68E-09	AT2G13550
chr1	21522263	6.61E-43	AT1G58150	chr2	5651361	3.57E-09	AT2G13550
chr1	21522285	3.38E-61	AT1G58150	chr2	5651547	2.58E-09	AT2G13550
chr1	21522329	1.77E-58	AT1G58150	chr2	5651569	9.85E-10	AT2G13550
chr1	21522335	2.41E-66	AT1G58150	chr2	5651721	6.7E-09	AT2G13550
chr1	21522385	1.56E-58	AT1G58150	chr2	5651835	4.22E-09	AT2G13550
chr1	21533577	4.64E-40	AT1G58150	chr2	5652071	1.77E-09	AT2G13550

chr1	21533612	5.31E-15	AT1G58150	chr2	5652081	5.02E-12	AT2G13550
chr1	21533678	3.2E-57	AT1G58150	chr2	5652083	5.21E-09	AT2G13550
chr1	21533832	1.56E-27	AT1G58150	chr2	5652201	2.45E-09	AT2G13550
chr1	21533862	5.01E-24	AT1G58150	chr2	5652218	8.61E-09	AT2G13550
chr1	21533885	3.45E-20	AT1G58150	chr2	5652226	2.79E-09	AT2G13550
chr1	21533897	1.05E-19	AT1G58150	chr2	5652344	3.36E-09	AT2G13550
chr1	21533928	4.23E-24	AT1G58150	chr2	5652356	5.76E-09	AT2G13550
chr1	21533929	2.72E-25	AT1G58150	chr2	5653174	5.85E-09	AT2G13550
chr1	21533939	1.18E-30	AT1G58150	chr2	5653342	2.53E-09	AT2G13550
chr1	21534028	1.22E-25	AT1G58150	chr2	5653346	4.44E-10	AT2G13550
chr1	21534085	1.27E-25	AT1G58150	chr2	5653543	4.68E-09	AT2G13550
chr1	21534104	5.79E-26	AT1G58150	chr2	5654064	3.68E-09	AT2G13550
chr1	21534127	5.49E-23	AT1G58150	chr2	5654124	2.78E-17	AT2G13550
chr1	21534194	1.38E-22	AT1G58150	chr2	5654217	5.55E-10	AT2G13550
chr1	21534242	4.27E-26	AT1G58150	chr2	5654401	6.41E-09	AT2G13550
chr1	21534463	1E-19	AT1G58150	chr2	5654412	5.14E-10	AT2G13550
chr1	21534470	1.3E-19	AT1G58150	chr2	5654430	5.59E-09	AT2G13550
chr1	21534621	6.82E-15	AT1G58150	chr2	6090582	3.16E-15	AT2G14460
chr1	21535692	1.83E-19	AT1G58150	chr2	6090702	9.12E-10	AT2G14460
chr1	21537144	2.05E-09	AT1G58150	chr2	6091370	1.69E-13	AT2G14460
chr1	21538591	9.19E-16	AT1G58150	chr2	6091388	8.16E-13	AT2G14460
chr1	23897635	4.38E-10	AT1G58150	chr2	6091439	2.88E-11	AT2G14460
chr1	28067673	3.16E-10	AT1G58150	chr2	6091459	9.66E-13	AT2G14460
chr2	4822036	3.77E-10	AT1G58150	chr2	6091470	5.36E-10	AT2G14460
chr2	7093736	2.25E-09	AT1G58150	chr2	6091568	1.39E-11	AT2G14460
chr2	9550629	4.33E-09	AT1G58150	chr2	6091604	2.58E-11	AT2G14460
chr2	11386765	1.05E-09	AT1G58150	chr2	6091606	2.73E-12	AT2G14460
chr3	14765622	5.34E-10	AT1G58150	chr2	6091676	6.94E-10	AT2G14460
chr3	15067064	7.79E-13	AT1G58150	chr2	6091682	4.74E-09	AT2G14460
chr3	15071963	3.03E-12	AT1G58150	chr2	6091941	4.88E-12	AT2G14460
chr3	16344717	6.07E-09	AT1G58150	chr2	6091981	3.25E-09	AT2G14460
chr3	17917774	8.6E-09	AT1G58150	chr2	6092010	6.3E-13	AT2G14460
chr3	23253812	1.38E-11	AT1G58150	chr2	6092048	6.87E-13	AT2G14460
chr3	23253890	2.48E-11	AT1G58150	chr2	6092063	1.3E-10	AT2G14460
chr3	23253932	4.66E-11	AT1G58150	chr2	6092091	1.33E-12	AT2G14460
chr3	23256604	1.24E-10	AT1G58150	chr2	6092115	1.03E-12	AT2G14460
chr4	7480285	4.09E-10	AT1G58150	chr2	6092121	1.05E-12	AT2G14460
chr5	17018132	2.19E-09	AT1G58150	chr2	6092142	3.57E-12	AT2G14460
chr5	17456079	1.83E-09	AT1G58150	chr2	6092143	3.57E-12	AT2G14460
chr5	19812805	3.14E-09	AT1G58150	chr2	6098210	1.75E-12	AT2G14460
chr5	23405087	2.01E-11	AT1G58150	chr2	6098318	5.86E-13	AT2G14460
chr1	16136057	5.86E-12	AT2G07000	chr2	6098349	4.44E-13	AT2G14460
chr1	16151332	6.46E-10	AT2G07000	chr2	6098360	1.03E-09	AT2G14460

chr1	16155322	1.05E-10	AT2G07000	chr2	6098364	5.38E-14	AT2G14460
chr1	16156821	6.11E-10	AT2G07000	chr2	6098404	4.49E-12	AT2G14460
chr1	16165048	6.81E-10	AT2G07000	chr2	6098688	3.53E-12	AT2G14460
chr1	16165914	1.31E-09	AT2G07000	chr2	6098701	2.87E-12	AT2G14460
chr1	16165983	1.29E-09	AT2G07000	chr2	6098717	1.18E-10	AT2G14460
chr1	16174051	4.87E-09	AT2G07000	chr2	6098778	3.75E-12	AT2G14460
chr1	16174173	6.16E-10	AT2G07000	chr2	6098822	2.7E-10	AT2G14460
chr1	16174867	5.08E-09	AT2G07000	chr2	6098845	5.02E-12	AT2G14460
chr1	16175492	3.12E-09	AT2G07000	chr2	6098887	1.04E-09	AT2G14460
chr1	16176221	7.97E-10	AT2G07000	chr2	6142864	1.34E-11	AT2G14460
chr1	16176950	9.79E-11	AT2G07000	chr2	6142898	2.63E-16	AT2G14460
chr1	16177149	1.18E-09	AT2G07000	chr2	6143009	3.2E-11	AT2G14460
chr1	16177314	4.84E-09	AT2G07000	chr2	6143028	6.21E-12	AT2G14460
chr1	16178211	6.53E-10	AT2G07000	chr2	6143029	2.66E-12	AT2G14460
chr1	16178879	1.79E-09	AT2G07000	chr2	6143055	4.4E-17	AT2G14460
chr1	16178892	4.79E-09	AT2G07000	chr2	6143071	4.81E-17	AT2G14460
chr1	16179560	4.04E-09	AT2G07000	chr2	6143505	3.04E-12	AT2G14460
chr1	16179980	2.09E-09	AT2G07000	chr2	6143513	2.54E-12	AT2G14460
chr1	16180035	5.52E-09	AT2G07000	chr2	6144433	6.02E-09	AT2G14460
chr1	16180036	5.52E-09	AT2G07000	chr2	6146955	9.35E-13	AT2G14460
chr1	16181592	2.24E-10	AT2G07000	chr2	6167556	1.19E-11	AT2G14460
chr1	16181708	2.55E-10	AT2G07000	chr2	6167854	7.93E-14	AT2G14460
chr1	16183256	1.09E-09	AT2G07000	chr2	6170932	7.07E-12	AT2G14460
chr1	16183583	5.18E-10	AT2G07000	chr2	6170972	7.68E-16	AT2G14460
chr1	16184647	6.47E-10	AT2G07000	chr2	6171007	7.76E-12	AT2G14460
chr1	16185037	1.04E-09	AT2G07000	chr4	1825844	9.84E-10	AT2G14460
chr1	16230580	1.15E-10	AT2G07000	chr4	17182830	1.69E-09	AT4G36515
chr1	16231756	7.96E-11	AT2G07000	chr4	17210051	7.09E-10	AT4G36515
chr1	22159394	4.69E-09	AT2G07000	chr4	17214378	5.55E-10	AT4G36515
chr2	2328513	6.58E-09	AT2G07000	chr4	17218046	1.26E-09	AT4G36515
chr2	2328520	8.61E-09	AT2G07000	chr4	17223056	7.72E-20	AT4G36515
chr2	2328588	6.32E-09	AT2G07000	chr4	17226115	1.61E-14	AT4G36515
chr2	2345392	2.08E-09	AT2G07000	chr4	17226241	3.74E-15	AT4G36515
chr2	2438030	6.06E-09	AT2G07000	chr4	17226394	3.22E-11	AT4G36515
chr2	2737016	7.06E-09	AT2G07000	chr4	17226403	5.55E-11	AT4G36515
chr2	2902135	3.7E-16	AT2G07000	chr4	17229388	2.11E-15	AT4G36515
chr2	2907244	3.33E-15	AT2G07000	chr4	17229450	8.41E-16	AT4G36515
chr2	2907260	9.6E-14	AT2G07000	chr4	17229716	5.96E-31	AT4G36515
chr2	2996309	2.62E-09	AT2G07000	chr4	17232022	3.35E-09	AT4G36515
chr2	3005405	9.32E-10	AT2G07000	chr4	17232807	2.31E-09	AT4G36515
chr2	3008715	2.84E-09	AT2G07000	chr4	17233141	6.61E-12	AT4G36515
chr2	3026562	5.85E-09	AT2G07000	chr4	17233164	1.15E-13	AT4G36515
chr2	3026969	5.16E-09	AT2G07000	chr4	17233301	4.3E-13	AT4G36515

chr2	3028232	7.42E-10	AT2G07000	chr4	17233328	1.57E-11	AT4G36515
chr2	3030344	2.35E-09	AT2G07000	chr4	17233354	1.6E-12	AT4G36515
chr2	3032416	5.07E-10	AT2G07000	chr4	17233358	4.01E-09	AT4G36515
chr2	3044895	4.93E-09	AT2G07000	chr4	17233360	6.07E-14	AT4G36515
chr2	3121488	3E-09	AT2G07000	chr4	17233366	1E-13	AT4G36515
chr2	3577341	8.11E-09	AT2G07000	chr4	17233394	3.95E-14	AT4G36515
chr2	4034117	2.58E-09	AT2G07000	chr4	17233505	1.48E-13	AT4G36515
chr2	4034225	1.05E-09	AT2G07000	chr4	17233987	4.88E-10	AT4G36515
chr2	4045069	2.09E-10	AT2G07000	chr4	17233988	2.82E-09	AT4G36515
chr2	4049607	1.85E-09	AT2G07000	chr4	17234020	1.02E-13	AT4G36515
chr2	4053072	2.91E-10	AT2G07000	chr4	17234033	3.67E-10	AT4G36515
chr2	4077208	1.17E-09	AT2G07000	chr4	17234266	4.24E-15	AT4G36515
chr2	4109013	2.9E-10	AT2G07000	chr4	17234659	2.84E-13	AT4G36515
chr2	4123402	4E-09	AT2G07000	chr4	17234674	7.86E-14	AT4G36515
chr2	4123430	8.77E-09	AT2G07000	chr4	17234807	7.32E-15	AT4G36515
chr2	4126905	8.24E-09	AT2G07000	chr4	17234827	7.03E-15	AT4G36515
chr2	4129315	8.36E-09	AT2G07000	chr4	17234845	6.39E-14	AT4G36515
chr2	4135671	7.34E-09	AT2G07000	chr5	26609279	4.18E-09	AT5G67245
chr2	4140458	3.19E-10	AT2G07000	chr5	26610570	6.25E-09	AT5G67245
chr2	5595999	1.8E-12	AT2G13550	chr5	26610584	6.25E-09	AT5G67245
chr2	5598123	3.84E-15	AT2G13550	chr5	26790286	7.48E-10	AT5G67245

Supplementary Table 2. Adjacent genes with eGWAS significance signals for *SWK*.

Chromosome	Start	End	Orientation	Gene ID	Annotation
Chr1	91376	95651	+	AT1G01220	protein_coding_gene
Chr1	95987	97407	+	AT1G01225	protein_coding_gene
Chr1	352637	354969	-	AT1G02020	protein_coding_gene
Chr1	1303529	1307884	-	AT1G04680	protein_coding_gene
Chr1	15508897	15512095	-	AT1G41740	transposable_element_gene
Chr1	18166147	18170105	-	AT1G49100	protein_coding_gene
Chr1	18171595	18172450	+	AT1G49110	protein_coding_gene
Chr1	20777727	20778130	+	AT1G55604	pseudogene
Chr1	20779874	20784017	-	AT1G55610	protein_coding_gene
Chr1	21128912	21130839	+	AT1G56423	protein_coding_gene
Chr1	21136944	21138087	+	AT1G56430	protein_coding_gene
Chr1	26007465	26009059	-	AT1G69180	protein_coding_gene
Chr1	26013012	26014834	-	AT1G69190	protein_coding_gene
Chr2	1989194	1989867	+	AT2G05435	transposable_element_gene
Chr2	1993082	1994166	+	AT2G05440	protein_coding_gene
Chr2	7363121	7363312	-	AT2G16955	protein_coding_gene
Chr2	7364683	7368963	+	AT2G16960	protein_coding_gene
Chr3	8480025	8482157	+	AT3G23620	protein_coding_gene
Chr3	8488695	8490210	+	AT3G23630	protein_coding_gene
Chr3	14914008	14918667	+	AT3G42806	transposable_element_gene
Chr3	14919657	14920215	+	AT3G42810	transposable_element_gene
Chr3	17106790	17110692	+	AT3G46484	transposable_element_gene
Chr4	1823795	1824970	-	AT4G03876	transposable_element_gene
Chr4	1828256	1830001	-	AT4G03880	transposable_element_gene
Chr4	11228187	11229577	+	AT4G21020	protein_coding_gene
Chr4	11231414	11231998	+	AT4G21030	protein_coding_gene
Chr4	16515327	16519534	+	AT4G34580	protein_coding_gene
Chr4	16521861	16523230	+	AT4G34588	protein_coding_gene
Chr4	17051982	17055059	-	AT4G36050	protein_coding_gene
Chr4	17052347	17053499	+	AT4G36052	other_RNA
Chr4	17055321	17056704	+	AT4G36060	protein_coding_gene
Chr4	17225912	17226610	-	AT4G36500	protein_coding_gene
Chr4	17228777	17229451	+	AT4G36510	protein_coding_gene
Chr5	1402045	1403961	-	AT5G04830	protein_coding_gene
Chr5	8437085	8439104	+	AT5G24650	protein_coding_gene
Chr5	16544340	16549280	+	AT5G41360	protein_coding_gene
Chr5	16551119	16556003	+	AT5G41370	protein_coding_gene
Chr5	16561278	16563732	-	AT5G41380	protein_coding_gene

Chr5	16565303	16567483	+	AT5G41390	protein_coding_gene
Chr5	16569377	16570177	-	AT5G41400	protein_coding_gene
Chr5	16572025	16572189	-	AT5G41401	protein_coding_gene
Chr5	16580044	16584008	+	AT5G41410	protein_coding_gene
Chr5	16585119	16585316	-	AT5G41420	protein_coding_gene
Chr5	16586119	16586604	-	AT5G41430	protein_coding_gene
Chr5	16587235	16587609	-	AT5G41440	protein_coding_gene
Chr5	16588600	16589094	-	AT5G41450	protein_coding_gene
Chr5	16589598	16592241	-	AT5G41460	protein_coding_gene
Chr5	16593953	16595568	+	AT5G41470	protein_coding_gene
Chr5	16595927	16598636	+	AT5G41480	protein_coding_gene
Chr5	16598796	16598905	+	AT5G41471	snoRNA
Chr5	16600445	16601608	+	AT5G41490	protein_coding_gene
Chr5	16601970	16602446	-	AT5G41491	pseudogene
Chr5	16602589	16603438	+	AT5G41494	pseudogene
Chr5	16604002	16605213	+	AT5G41500	protein_coding_gene
Chr5	16605491	16606228	-	AT5G41505	transposable_element_gene
Chr5	16607124	16608326	+	AT5G41510	protein_coding_gene
Chr5	16609153	16610692	-	AT5G41520	protein_coding_gene
Chr5	16611660	16612088	+	AT5G41530	protein_coding_gene
Chr5	19616244	19619246	+	AT5G48400	protein_coding_gene
Chr5	24494691	24496351	+	AT5G60890	protein_coding_gene
Chr5	24498467	24501494	-	AT5G60900	protein_coding_gene

Supplementary Table 3. Genes whose mutations changed *SWK* expression level two times based on the analysis of published RNA-seq data.

Project name	WT FPKM mean	Mutant FPKM mean	WT SD	Mutant SD	log_2Fold Change	Mutated gene
PRJNA513324	17.64	0.61	16.91	0.05	-4.11	AT1G63020
PRJNA256037	3.50	0.33	3.26	0.38	-3.15	AT1G70560
PRJNA292478	4.86	0.72	0.58	0.27	-2.73	AT4G37750<br>AT5G10510
PRJNA225010	11.07	1.61	5.56	0.38	-2.72	AT1G04240
PRJNA320883	5.48	0.87	1.84	0.38	-2.57	AT4G18960<br>AT4G36920
SRP012592	9.60	1.74	2.41	0.30	-2.41	AT5G03730
PRJNA471077	7.65	1.71	7.28	0.13	-2.36	AT4G01290
PRJNA436824	3.94	0.83	1.45	0.27	-2.18	AT3G14110
PRJNA433852	58.64	13.09	2.37	1.05	-2.17	AT1G75080
PRJNA320883	5.48	1.25	1.84	0.53	-2.12	AT4G18960<br>AT4G36920
PRJNA412814	1.60	0.49	0.38	0.26	-1.93	AT5G55760
PRJNA316322	14.22	3.79	1.25	0.18	-1.91	AT2G20180<br>AT1G09530<br>AT2G43010<br>AT3G59060
PRJNA261433	2.78	0.71	0.39	0.08	-1.86	AT2G23380
PRJNA394192	200.60	64.35	66.37	4.79	-1.77	AT3G24650
PRJNA262177	2.78	0.93	0.39	0.21	-1.72	AT1G26110
PRJNA259643	8.15	2.63	0.71	0.29	-1.53	AT2G20180<br>AT1G09530<br>AT2G43010<br>AT3G59060
PRJNA412787	2.65	1.05	1.78	0.29	-1.36	AT1G03140
PRJNA300685	23.20	9.29	5.66	3.39	-1.26	AT5G66750
PRJNA383483	7.06	3.24	2.20	0.17	-1.23	AT5G37020
PRJNA494862	1.46	0.65	0.15	0.26	-1.18	AT5G60300
PRJNA318638	6.60	3.21	0.73	0.43	-1.03	AT3G44450
PRJNA301162	50.48	96.64	4.30	28.60	1.02	AT2G30470
PRJNA349095	3.14	6.71	0.20	0.74	1.12	AT3G18520
PRJNA448672	6.92	16.48	0.82	1.26	1.27	AT2G46340<br>AT4G11110<br>AT3G15354<br>AT1G53090

PRJNA339870	2.87	7.19	0.52	2.13	1.28	AT1G76810
PRJNA430092	3.26	7.89	0.47	0.45	1.29	AT1G21970
PRJNA427587	1.86	4.71	0.18	1.71	1.35	AT1G21970
PRJNA475456	1.14	3.11	0.46	0.27	1.46	AT4G37580
PRJNA247731	4.84	15.16	1.37	2.41	1.68	AT1G14920<br>AT2G01570<br>AT4G04970<br>AT1G78860
PRJNA427587	12.96	42.89	3.68	1.89	1.75	AT1G14920
PRJNA232535	1.92	7.05	0.41	0.83	1.83	AT3G57860
PRJNA401366	0.95	3.55	0.19	1.91	1.88	AT2G39340
PRJNA401366	0.95	3.61	0.19	2.08	1.92	AT3G25840
PRJNA363063	1.16	4.87	0.01	0.72	2.04	AT2G22400
PRJNA430092	1.63	6.59	0.46	0.44	2.05	AT5G54310
PRJNA481068	9.41	43.29	1.90	3.86	2.19	AT2G18790
PRJNA449911	4.13	19.39	1.26	3.44	2.21	AT5G10790
PRJNA431488	0.57	3.60	0.10	1.00	2.31	AT1G77760
PRJNA388036	4.36	21.58	0.37	9.54	2.33	AT3G46640
PRJNA512228	1.40	7.38	0.80	1.07	2.43	AT5G63110
PRJNA302602	4.27	24.24	1.83	6.29	2.48	AT1G55480
PRJNA307295	3.25	19.66	0.63	0.74	2.63	AT2G32260
PRJNA301162	10.25	65.39	1.38	9.61	2.73	AT2G30470
PRJNA326696	1.49	11.60	1.13	2.57	2.74	AT5G17220
PRJNA378644	1.52	10.63	0.07	1.87	2.83	AT1G11720<br>AT4G18240
PRJNA430092	1.63	11.93	0.46	2.41	2.86	AT5G54310<br>AT1G71830
PRJNA436256	0.44	3.05	0.17	0.66	2.87	AT1G80490
PRJNA232535	1.92	15.38	0.41	2.28	2.99	AT3G57860<br>AT1G55480
PRJNA314692	0.41	4.09	0.29	0.36	3.19	AT2G18790
PRJNA454174	2.10	25.62	0.00	1.39	3.58	AT5G53430
PRJNA305309	15.56	185.00	1.03	29.11	3.63	AT3G11100
PRJNA232535	1.92	24.60	0.41	7.85	3.68	AT1G03080
SRP013336	3.11	51.98	0.67	2.41	3.69	AT2G31400<br>AT2G36990
PRJNA329634	0.59	8.98	0.14	2.60	3.83	AT4G26930<br>AT2G32460<br>AT5G55020
PRJNA147225	0.82	25.78	0.21	3.30	3.84	AT1G77180
PRJNA352036	5.13	80.09	1.06	3.07	3.91	AT2G32950
PRJNA354686	4.79	80.81	0.99	3.24	4.04	AT2G32950
PRJNA505783	1.28	22.90	1.27	1.52	4.14	AT4G08150

PRJNA384596	0.49	9.61	0.22	0.45	4.17	AT2G25170
PRJNA522747	0.41	15.35	0.29	1.12	5.20	AT4G32940
PRJNA320769	2.17	118.85	1.93	5.73	5.76	AT2G23380<br>AT4G02020
PRJNA320769	1.45	107.52	0.40	3.25	6.13	AT2G23380<br>AT4G02020
PRJNA326392	0.81	84.94	0.00	1.93	6.66	AT1G06770<br>AT2G30580
PRJNA326392	0.81	94.76	0.00	2.52	6.81	AT1G06770<br>AT2G30580<br>AT3G23060

WT: Col-0.

SD: Standard Deviation.

Genes highlighted in yellow: four genes involved in regulating seed germination.

Genes highlighted in light blue: 22 genes involved in regulating seed germination under various conditions.

Supplementary Table 4. KEGG enrichment analysis of genes in supplementary Table 3.

KEGG pathway	Input number	Background number	*p*-value	Corrected *p*-value	Input genes
Circadian rhythm - plant	4	36	1.74E-06	5.33E-05	AT2G46340<br>AT1G09530<br>AT2G18790<br>AT2G32950
Plant hormone signal transduction	7	273	2.56E-06	6.23E-05	AT5G03730<br>AT1G75080<br>AT1G09530<br>AT1G04240<br>AT2G01570<br>AT1G14920<br>AT2G43010
Lysine degradation	2	34	0.002777	0.014134	AT4G02020<br>AT2G23380
Phosphonate and phosphinate metabolism	1	6	0.015061	0.047997	AT2G32260
Starch and sucrose metabolism	2	165	0.050979	0.085635	AT1G11720<br>AT4G18240
Spliceosome	2	192	0.066363	0.101163	AT1G77180<br>AT1G03140
Nitrogen metabolism	1	43	0.091029	0.124552	AT1G77760
Tryptophan metabolism	1	60	0.123967	0.155988	AT1G70560
Glycerophospholipid metabolism	1	97	0.191642	0.225345	AT2G32260
Glutathione metabolism	1	102	0.200383	0.232417	AT5G17220
MAPK signaling pathway - plant	1	134	0.254158	0.283228	AT5G03730
Ubiquitin mediated proteolysis	1	143	0.268628	0.295491	AT2G32950
Endocytosis	1	144	0.270219	0.296285	AT5G54310
RNA transport	1	171	0.311901	0.337645	AT1G76810
Metabolic pathways	6	2246	0.361463	0.385184	AT4G18240<br>AT5G17220<br>AT1G11720<br>AT2G32260<br>AT1G70560<br>AT1G77760
Biosynthesis of secondary metabolites	2	1107	0.697422	0.718474	AT1G11720<br>AT4G18240

Databases: KEGG PATHWAY.

Statistical test method: hypergeometric test / Fisher's exact test.

FDR correction method: Benjamini and Hochberg.

Supplementary Table 5. Differentially expressed genes (DEGs) identified based on RNA-seq data.

Gene	Log_2Fold Change	Corrected *p*-value	Compare materials	Gene	Log_2Fold Change	Corrected *p*-value	Compare materials
AT2G41640	-2.40266	9.19E-61	MT VS. WT	AT1G74410	-0.60533	0.012024	OE VS. MT
AT1G75945	5.073203	2.58E-42	MT VS. WT	AT2G36970	-0.54696	0.012088	OE VS. MT
AT2G41650	1.160162	8.5E-32	MT VS. WT	AT1G04520	0.544396	0.012098	OE VS. MT
AT4G31500	-0.82136	3.04E-23	MT VS. WT	AT3G62600	0.502402	0.012107	OE VS. MT
AT3G41762	7.586304	5.11E-11	MT VS. WT	AT5G61000	0.543142	0.012505	OE VS. MT
AT1G74100	-0.52319	2.84E-09	MT VS. WT	AT3G53040	-0.77488	0.012781	OE VS. MT
AT2G30860	-0.51491	2.68E-08	MT VS. WT	AT1G48750	-0.59345	0.012781	OE VS. MT
AT1G65970	0.79809	1.78E-07	MT VS. WT	AT1G17500	0.398869	0.012781	OE VS. MT
AT1G73220	-0.7369	3.2E-07	MT VS. WT	AT1G65620	1.048033	0.012781	OE VS. MT
AT2G20610	-0.46562	4.31E-07	MT VS. WT	AT5G07360	-0.35762	0.012802	OE VS. MT
AT2G43510	0.688869	7.84E-06	MT VS. WT	AT4G16160	-0.34497	0.012872	OE VS. MT
AT5G06860	1.212313	1.08E-05	MT VS. WT	AT2G39890	0.374096	0.012922	OE VS. MT
AT4G04610	-0.50284	5.19E-05	MT VS. WT	AT1G68240	-0.46248	0.013397	OE VS. MT
AT2G36640	0.385007	7.25E-05	MT VS. WT	AT4G26740	-1.21007	0.013419	OE VS. MT
AT2G38860	-0.51057	8.12E-05	MT VS. WT	AT2G45560	-0.41849	0.013465	OE VS. MT
AT5G06760	0.42359	0.000198	MT VS. WT	AT1G32710	-1.11704	0.013603	OE VS. MT
AT1G61800	-0.62516	0.000503	MT VS. WT	AT3G07500	-0.66539	0.013603	OE VS. MT
AT2G14750	-0.49435	0.000503	MT VS. WT	AT4G27460	-0.52494	0.013603	OE VS. MT
AT2G30750	3.705097	0.000503	MT VS. WT	AT3G51510	-0.49037	0.013603	OE VS. MT
AT4G30530	-0.43145	0.00085	MT VS. WT	AT4G36540	0.553756	0.013603	OE VS. MT
AT4G30290	-0.47632	0.001299	MT VS. WT	AT3G57880	-0.37492	0.013746	OE VS. MT
AT5G07200	-0.62839	0.001495	MT VS. WT	AT3G29320	0.311786	0.013752	OE VS. MT
AT2G01280	1.702705	0.001864	MT VS. WT	AT3G21090	0.314269	0.013776	OE VS. MT
AT1G52690	0.40423	0.001913	MT VS. WT	AT1G64900	-0.66687	0.013912	OE VS. MT
AT5G52300	0.511537	0.00206	MT VS. WT	AT2G30140	-0.46475	0.014058	OE VS. MT
AT1G08590	-0.46915	0.00291	MT VS. WT	AT3G62700	-0.25986	0.014171	OE VS. MT
AT5G50360	0.412534	0.003034	MT VS. WT	AT5G45930	0.572575	0.014171	OE VS. MT
AT5G02260	-0.39647	0.003175	MT VS. WT	AT4G22220	-0.32478	0.014185	OE VS. MT
AT2G40610	-0.41509	0.005897	MT VS. WT	AT5G26667	0.420248	0.014185	OE VS. MT
AT5G11420	-0.29888	0.006105	MT VS. WT	AT1G18250	0.671749	0.014185	OE VS. MT
AT1G78850	0.68812	0.006739	MT VS. WT	AT5G23210	1.582574	0.014222	OE VS. MT
AT1G17860	0.823802	0.007708	MT VS. WT	AT3G21710	1.788327	0.014222	OE VS. MT
AT2G05520	2.012236	0.009596	MT VS. WT	AT1G14940	-1.14607	0.014376	OE VS. MT
AT3G16150	-0.54847	0.013052	MT VS. WT	AT3G14370	1.265052	0.014383	OE VS. MT
AT1G76790	-0.33383	0.016419	MT VS. WT	AT1G06570	-0.69933	0.01446	OE VS. MT
AT3G20110	-0.84342	0.017638	MT VS. WT	AT2G30970	0.265479	0.01446	OE VS. MT
AT2G30870	-0.58707	0.017638	MT VS. WT	AT5G19740	0.29523	0.014637	OE VS. MT

AT3G44570	-5.70497	0.02121	MT VS. WT	AT5G60760	-0.36764	0.014751	OE VS. MT
AT1G24100	-0.40035	0.02121	MT VS. WT	AT5G59170	-1.15427	0.014787	OE VS. MT
AT5G02020	0.515929	0.023993	MT VS. WT	AT1G35560	-0.69944	0.014935	OE VS. MT
AT3G23820	-0.5593	0.028182	MT VS. WT	AT4G15790	0.622519	0.014935	OE VS. MT
AT4G10380	-0.46888	0.037248	MT VS. WT	AT1G80280	0.359132	0.015126	OE VS. MT
AT1G13080	-0.49616	0.042907	MT VS. WT	AT1G16850	-0.61975	0.015255	OE VS. MT
AT1G20010	-0.43235	0.046916	MT VS. WT	AT2G01830	-0.35591	0.015255	OE VS. MT
AT1G75945	-7.75725	4.58E-48	OE VS. MT	AT2G22795	0.333275	0.015272	OE VS. MT
AT4G31500	0.855018	1.17E-21	OE VS. MT	AT2G20770	-0.65044	0.01566	OE VS. MT
AT4G19030	1.54814	9.32E-11	OE VS. MT	AT2G40080	0.302641	0.01566	OE VS. MT
AT2G30860	0.724402	4.88E-10	OE VS. MT	AT5G04160	0.778996	0.01566	OE VS. MT
AT5G24150	0.958325	4.88E-10	OE VS. MT	AT2G03090	0.320696	0.015691	OE VS. MT
AT4G25580	-0.81362	9.76E-10	OE VS. MT	AT5G67100	0.389911	0.015691	OE VS. MT
AT3G58450	-0.7628	4E-09	OE VS. MT	AT4G37410	0.758572	0.015803	OE VS. MT
AT1G05570	0.675462	7E-09	OE VS. MT	AT3G63040	-1.09159	0.015919	OE VS. MT
AT1G73220	0.663022	8.87E-09	OE VS. MT	AT3G22750	-0.63115	0.015949	OE VS. MT
AT2G38860	0.55363	3.3E-08	OE VS. MT	AT1G15740	-0.54495	0.015949	OE VS. MT
AT4G39950	1.300381	3.3E-08	OE VS. MT	AT5G62900	-0.45592	0.015949	OE VS. MT
AT3G09390	-0.44055	4.71E-07	OE VS. MT	AT2G17520	-0.38961	0.015949	OE VS. MT
AT3G23820	0.916304	6.83E-07	OE VS. MT	AT2G46450	0.289646	0.015949	OE VS. MT
AT4G19120	1.044692	8.22E-07	OE VS. MT	AT1G47210	0.585831	0.015949	OE VS. MT
AT5G54000	-1.27355	1.09E-06	OE VS. MT	AT3G51810	-0.73809	0.016014	OE VS. MT
AT5G55240	-0.82254	1.09E-06	OE VS. MT	AT1G36060	-0.54587	0.016403	OE VS. MT
AT1G01470	-0.75958	1.57E-06	OE VS. MT	AT4G14960	0.317245	0.016403	OE VS. MT
AT2G22170	1.138249	1.59E-06	OE VS. MT	AT3G19820	0.225425	0.017091	OE VS. MT
AT1G62570	0.826863	3.49E-06	OE VS. MT	AT1G69295	-0.28996	0.017137	OE VS. MT
AT1G71020	0.785885	4.15E-06	OE VS. MT	AT1G02850	-0.36202	0.017257	OE VS. MT
AT4G30530	0.553611	5.43E-06	OE VS. MT	AT2G29980	0.333324	0.017378	OE VS. MT
AT5G02260	0.450346	5.68E-06	OE VS. MT	AT3G25500	0.416721	0.017423	OE VS. MT
AT4G38740	-0.71833	8.1E-06	OE VS. MT	AT1G75280	-0.64495	0.017797	OE VS. MT
AT5G01040	0.759789	8.1E-06	OE VS. MT	AT1G01650	-0.49422	0.017797	OE VS. MT
AT1G77950	-0.75512	8.2E-06	OE VS. MT	AT5G13800	-0.36461	0.017853	OE VS. MT
AT2G23640	-0.73967	8.2E-06	OE VS. MT	AT1G69890	-0.66633	0.017869	OE VS. MT
AT5G62490	-0.52996	8.2E-06	OE VS. MT	AT2G19170	0.600995	0.017869	OE VS. MT
AT2G24280	0.417254	8.2E-06	OE VS. MT	AT5G65360	0.461516	0.017947	OE VS. MT
AT1G08590	0.599051	8.2E-06	OE VS. MT	AT4G31320	0.615748	0.017947	OE VS. MT
AT5G51760	-0.50584	1.57E-05	OE VS. MT	AT2G04160	0.964207	0.017947	OE VS. MT
AT4G09610	-2.71669	1.65E-05	OE VS. MT	AT2G32090	-0.54903	0.018027	OE VS. MT
AT2G34740	-1.63479	1.65E-05	OE VS. MT	AT1G19530	-0.46484	0.018027	OE VS. MT
AT5G05270	1.735164	1.65E-05	OE VS. MT	AT5G51560	0.47709	0.018027	OE VS. MT
AT4G03200	-0.5963	1.72E-05	OE VS. MT	AT3G60140	-0.76907	0.018248	OE VS. MT
AT5G08640	2.139681	2.36E-05	OE VS. MT	AT4G13010	-0.37512	0.018302	OE VS. MT
AT1G47540	-1.02425	2.39E-05	OE VS. MT	AT4G13930	0.280515	0.018361	OE VS. MT

AT1G13080	0.659638	2.77E-05	OE VS. MT	AT2G33520	-0.84806	0.018419	OE VS. MT
AT3G52470	0.665807	2.88E-05	OE VS. MT	AT3G23800	0.395886	0.018497	OE VS. MT
AT2G19930	-0.52093	3.39E-05	OE VS. MT	AT2G27510	-0.26884	0.018499	OE VS. MT
AT5G20400	0.524412	3.39E-05	OE VS. MT	AT3G51820	0.451992	0.018499	OE VS. MT
AT2G20610	0.568408	3.39E-05	OE VS. MT	AT1G08930	0.609545	0.018525	OE VS. MT
AT1G56600	-1.05732	3.67E-05	OE VS. MT	AT1G43245	-0.43646	0.018671	OE VS. MT
AT2G03520	-0.59936	4.99E-05	OE VS. MT	AT2G17560	0.384724	0.018671	OE VS. MT
AT1G74100	0.478993	4.99E-05	OE VS. MT	AT5G23730	1.367475	0.01899	OE VS. MT
AT3G26770	-0.9635	5.22E-05	OE VS. MT	AT1G07430	-0.39206	0.019025	OE VS. MT
AT2G38800	-0.92704	5.22E-05	OE VS. MT	AT1G03990	-0.45545	0.01912	OE VS. MT
AT1G65970	-1.08812	7.87E-05	OE VS. MT	AT2G16440	0.421421	0.01912	OE VS. MT
AT1G02700	-0.51159	7.87E-05	OE VS. MT	AT3G23880	1.284388	0.01912	OE VS. MT
AT3G03620	-0.54701	8.02E-05	OE VS. MT	AT3G02180	0.747867	0.019129	OE VS. MT
AT2G35300	-0.52829	8.58E-05	OE VS. MT	AT2G38650	0.332886	0.019261	OE VS. MT
AT1G10960	0.524842	8.7E-05	OE VS. MT	AT2G28490	-1.08562	0.019458	OE VS. MT
AT3G14595	-0.66748	8.97E-05	OE VS. MT	AT4G36010	-0.41909	0.019655	OE VS. MT
AT2G32390	0.664593	8.97E-05	OE VS. MT	AT1G22710	0.338926	0.019682	OE VS. MT
AT2G27690	0.655829	9.26E-05	OE VS. MT	AT1G70940	0.500666	0.019772	OE VS. MT
AT5G51750	0.581342	9.86E-05	OE VS. MT	AT4G10380	0.39681	0.019818	OE VS. MT
AT3G48140	0.834216	9.99E-05	OE VS. MT	AT1G56280	-0.30776	0.019941	OE VS. MT
AT5G55930	1.204243	9.99E-05	OE VS. MT	AT2G46420	0.338869	0.01996	OE VS. MT
AT1G69530	0.363853	0.000105	OE VS. MT	AT1G53580	0.306789	0.020029	OE VS. MT
AT4G09600	-1.23882	0.000114	OE VS. MT	AT5G44790	-0.33144	0.020205	OE VS. MT
AT3G11050	-0.52952	0.000136	OE VS. MT	AT1G71110	0.222399	0.020243	OE VS. MT
AT4G12680	-0.50735	0.000139	OE VS. MT	AT5G01950	-0.3826	0.020255	OE VS. MT
AT5G55750	-1.22542	0.00014	OE VS. MT	AT5G63030	-0.71661	0.020376	OE VS. MT
AT5G65550	-0.80293	0.00014	OE VS. MT	AT1G13960	-0.44945	0.020622	OE VS. MT
AT5G66920	0.538186	0.00014	OE VS. MT	AT3G44290	-0.36556	0.020656	OE VS. MT
AT2G17710	0.851758	0.00014	OE VS. MT	AT5G16590	0.672862	0.020656	OE VS. MT
AT1G13340	-1.28321	0.000146	OE VS. MT	AT5G63810	0.309634	0.020659	OE VS. MT
AT3G03310	-0.49591	0.000149	OE VS. MT	AT1G11910	-0.26652	0.02093	OE VS. MT
AT1G09780	0.621834	0.000157	OE VS. MT	AT1G26665	-0.34864	0.021027	OE VS. MT
AT3G47950	-0.64532	0.000188	OE VS. MT	AT3G05910	0.805751	0.021027	OE VS. MT
AT5G53895	-1.14854	0.000198	OE VS. MT	AT4G27780	-0.42163	0.021141	OE VS. MT
AT1G29680	-1.14692	0.000198	OE VS. MT	AT2G23380	0.445329	0.021141	OE VS. MT
AT1G68570	-0.82748	0.000198	OE VS. MT	AT1G04430	0.589808	0.021141	OE VS. MT
AT1G07645	-0.64976	0.000198	OE VS. MT	AT4G37470	0.619652	0.021141	OE VS. MT
AT3G16150	0.529056	0.000198	OE VS. MT	AT1G27930	0.798336	0.021141	OE VS. MT
AT1G04020	0.915431	0.000199	OE VS. MT	AT1G14360	0.497109	0.021406	OE VS. MT
AT4G18650	-0.89438	0.00021	OE VS. MT	AT3G24480	0.493187	0.021419	OE VS. MT
AT5G67280	0.414847	0.000219	OE VS. MT	AT4G38780	-0.4679	0.021462	OE VS. MT
AT2G30870	0.741214	0.00025	OE VS. MT	AT5G50700	-3.25974	0.021633	OE VS. MT
AT1G09200	0.744058	0.00025	OE VS. MT	AT4G19700	-0.36561	0.021633	OE VS. MT

AT3G59760	0.529045	0.000255	OE VS. MT	AT1G08920	0.92298	0.0219	OE VS. MT
AT3G21600	-0.58975	0.000282	OE VS. MT	AT2G33740	-0.3724	0.021917	OE VS. MT
AT5G60360	0.546485	0.000285	OE VS. MT	AT5G46280	0.361098	0.022072	OE VS. MT
AT3G54500	-0.75929	0.000289	OE VS. MT	AT5G09650	0.316559	0.022153	OE VS. MT
AT3G50980	-0.54099	0.000289	OE VS. MT	AT1G68890	0.436808	0.022157	OE VS. MT
AT1G64950	-0.48677	0.000289	OE VS. MT	AT2G28200	1.229693	0.02233	OE VS. MT
AT2G29090	-0.39234	0.000289	OE VS. MT	AT1G12780	0.27925	0.022692	OE VS. MT
AT1G24100	0.521192	0.000289	OE VS. MT	AT1G15330	-0.40135	0.022814	OE VS. MT
AT4G31330	0.645342	0.000289	OE VS. MT	AT5G19290	0.422994	0.022973	OE VS. MT
AT5G58900	1.080741	0.000289	OE VS. MT	AT3G01770	-0.30897	0.023481	OE VS. MT
AT1G78070	-0.82232	0.000294	OE VS. MT	AT2G20920	-0.48748	0.02363	OE VS. MT
AT5G39720	-2.52851	0.000321	OE VS. MT	AT5G42860	1.724019	0.023675	OE VS. MT
AT2G33830	-0.73055	0.000321	OE VS. MT	AT2G41280	-1.21347	0.023774	OE VS. MT
AT2G18050	-1.05148	0.000326	OE VS. MT	AT1G67750	1.139373	0.023874	OE VS. MT
AT4G02280	-0.4992	0.000337	OE VS. MT	AT1G70810	-0.65441	0.023913	OE VS. MT
AT3G28899	-0.80238	0.000358	OE VS. MT	AT3G13470	0.282817	0.023913	OE VS. MT
AT1G26770	0.40605	0.000387	OE VS. MT	AT4G30800	0.307188	0.023913	OE VS. MT
AT5G43745	0.4343	0.000387	OE VS. MT	AT1G17220	0.250951	0.024036	OE VS. MT
AT3G62270	0.502034	0.000387	OE VS. MT	AT5G23420	0.491578	0.024036	OE VS. MT
AT3G54920	0.666789	0.00039	OE VS. MT	AT1G18190	0.360422	0.024277	OE VS. MT
AT3G48270	-0.63759	0.000423	OE VS. MT	AT3G54750	0.547628	0.024603	OE VS. MT
AT2G25890	-0.52965	0.000438	OE VS. MT	AT2G42000	-1.4653	0.024634	OE VS. MT
AT2G28420	-0.32384	0.000438	OE VS. MT	AT3G20110	0.707954	0.024634	OE VS. MT
AT1G76790	0.456435	0.000439	OE VS. MT	AT1G61840	1.325949	0.024634	OE VS. MT
AT5G57790	-0.57246	0.00046	OE VS. MT	AT3G27160	0.359198	0.024757	OE VS. MT
AT1G73680	0.69041	0.000465	OE VS. MT	AT2G38400	0.355429	0.024916	OE VS. MT
AT3G57780	0.68023	0.000476	OE VS. MT	AT2G39420	0.250048	0.025198	OE VS. MT
AT5G54740	-1.52562	0.000514	OE VS. MT	AT1G60160	0.285511	0.025198	OE VS. MT
AT1G08170	-1.12922	0.000518	OE VS. MT	AT1G10760	0.267839	0.025628	OE VS. MT
AT3G12490	-0.48644	0.00054	OE VS. MT	AT3G28180	0.386412	0.025844	OE VS. MT
AT3G47420	0.657332	0.000541	OE VS. MT	AT1G28580	0.303613	0.025912	OE VS. MT
AT2G21720	-0.40784	0.00055	OE VS. MT	AT3G46940	0.391098	0.026006	OE VS. MT
AT1G70280	0.481655	0.00055	OE VS. MT	AT4G27990	-0.5122	0.026238	OE VS. MT
AT3G15280	-0.5185	0.000704	OE VS. MT	AT2G13360	-0.48139	0.026238	OE VS. MT
AT4G38970	0.51413	0.000704	OE VS. MT	AT5G39850	-0.27017	0.026238	OE VS. MT
AT3G29140	-1.94171	0.00071	OE VS. MT	AT5G65750	-0.25276	0.026238	OE VS. MT
AT2G39210	-0.77468	0.00071	OE VS. MT	AT3G48000	-0.23947	0.026238	OE VS. MT
AT2G31350	-0.62208	0.000738	OE VS. MT	AT4G25170	-0.27938	0.026391	OE VS. MT
AT1G78060	0.377261	0.000759	OE VS. MT	AT4G09590	-1.23732	0.026416	OE VS. MT
AT4G21320	-0.79912	0.000766	OE VS. MT	AT1G57820	0.489663	0.026416	OE VS. MT
AT5G63190	-0.46191	0.000814	OE VS. MT	AT1G30500	-0.52058	0.026581	OE VS. MT
AT3G11930	-0.47337	0.000815	OE VS. MT	AT5G44310	-0.26982	0.026594	OE VS. MT
AT4G21490	1.585878	0.000818	OE VS. MT	AT1G66150	0.376405	0.026625	OE VS. MT

AT1G20440	0.505043	0.000833	OE VS. MT	AT5G03160	0.423152	0.026675	OE VS. MT
AT4G23990	-0.68263	0.000841	OE VS. MT	AT3G05260	-1.76097	0.026706	OE VS. MT
AT3G54940	-0.43767	0.000845	OE VS. MT	AT5G15650	0.282647	0.027273	OE VS. MT
AT5G25180	-1.25273	0.00087	OE VS. MT	AT5G17190	-0.26532	0.027355	OE VS. MT
AT1G28330	-0.5095	0.000871	OE VS. MT	AT4G18360	-0.4387	0.027385	OE VS. MT
AT2G44060	0.639202	0.000914	OE VS. MT	AT3G02870	0.436565	0.027385	OE VS. MT
AT4G17840	-0.84183	0.000957	OE VS. MT	AT2G31985	-0.92486	0.027439	OE VS. MT
AT5G49990	-0.49232	0.000966	OE VS. MT	AT5G52950	0.64814	0.027769	OE VS. MT
AT1G01720	-0.49536	0.000983	OE VS. MT	AT1G25422	-0.81675	0.027785	OE VS. MT
AT5G46250	-0.46116	0.000989	OE VS. MT	AT5G66780	-0.31397	0.027785	OE VS. MT
AT4G38810	-0.36289	0.000989	OE VS. MT	AT1G30630	0.283734	0.027785	OE VS. MT
AT4G30290	0.499513	0.000997	OE VS. MT	AT4G13710	0.823574	0.027872	OE VS. MT
AT3G15260	-0.46217	0.001025	OE VS. MT	AT5G56260	0.229214	0.027951	OE VS. MT
AT5G66110	-0.97965	0.00107	OE VS. MT	AT1G75780	0.84512	0.02814	OE VS. MT
AT3G19930	0.439809	0.00107	OE VS. MT	AT3G18370	-0.31112	0.028161	OE VS. MT
AT5G17310	-0.60769	0.001081	OE VS. MT	AT3G61820	0.348418	0.028161	OE VS. MT
AT4G34600	0.964391	0.001148	OE VS. MT	AT2G33770	-0.32243	0.028168	OE VS. MT
AT1G03470	-0.72987	0.001216	OE VS. MT	AT3G04360	-0.83891	0.028206	OE VS. MT
AT1G16770	-0.9936	0.001238	OE VS. MT	AT5G40760	-0.42325	0.028576	OE VS. MT
AT1G24735	-0.59062	0.001238	OE VS. MT	AT4G20230	1.241829	0.028668	OE VS. MT
AT4G39940	0.836699	0.001238	OE VS. MT	AT5G53220	-0.61524	0.02869	OE VS. MT
AT1G18100	-0.98009	0.001247	OE VS. MT	AT5G03560	-0.32492	0.02869	OE VS. MT
AT1G15210	0.477154	0.001251	OE VS. MT	AT1G75680	0.374079	0.028774	OE VS. MT
AT5G14780	-0.54779	0.001278	OE VS. MT	AT3G27660	-1.74447	0.028809	OE VS. MT
AT1G41830	0.4629	0.001288	OE VS. MT	AT5G17270	-0.22557	0.02885	OE VS. MT
AT2G32240	0.330541	0.001323	OE VS. MT	AT1G27990	-0.91593	0.02919	OE VS. MT
AT1G07890	0.419358	0.001323	OE VS. MT	AT1G71950	-0.37334	0.02919	OE VS. MT
AT5G57090	0.520056	0.001323	OE VS. MT	AT3G61990	-0.2606	0.02919	OE VS. MT
AT4G30200	0.461555	0.001339	OE VS. MT	AT2G16365	0.433371	0.02933	OE VS. MT
AT3G19920	-0.36442	0.00134	OE VS. MT	AT4G34810	1.448039	0.02933	OE VS. MT
AT5G59700	-0.57826	0.001447	OE VS. MT	AT3G61580	0.433959	0.029424	OE VS. MT
AT1G80460	0.391464	0.001461	OE VS. MT	AT5G04500	-1.32274	0.029572	OE VS. MT
AT2G33330	0.673269	0.001504	OE VS. MT	AT2G23320	-0.40248	0.030073	OE VS. MT
AT2G01280	-1.67711	0.001526	OE VS. MT	AT3G21370	-1.24961	0.030142	OE VS. MT
AT1G30860	-1.04553	0.00156	OE VS. MT	AT1G12000	0.299073	0.030142	OE VS. MT
AT1G28290	0.398312	0.00156	OE VS. MT	AT1G14860	0.465746	0.030142	OE VS. MT
AT5G66310	0.564573	0.001562	OE VS. MT	AT5G01600	0.549031	0.030142	OE VS. MT
AT2G01190	-0.44395	0.001613	OE VS. MT	AT5G65640	0.293678	0.03033	OE VS. MT
AT2G38310	0.39732	0.001794	OE VS. MT	AT2G17630	0.429645	0.030509	OE VS. MT
AT5G52420	-0.46165	0.001802	OE VS. MT	AT2G41260	-0.99485	0.030615	OE VS. MT
AT1G75490	-0.79545	0.001805	OE VS. MT	AT5G42820	-0.27488	0.030615	OE VS. MT
AT3G59970	0.393256	0.001806	OE VS. MT	AT5G01300	-0.93663	0.030915	OE VS. MT
AT5G49460	0.52401	0.001806	OE VS. MT	AT5G07340	0.342808	0.031072	OE VS. MT

AT2G14750	0.488021	0.001909	OE VS. MT	AT1G07400	-1.66601	0.031379	OE VS. MT
AT2G31270	0.661259	0.001909	OE VS. MT	AT5G03380	-0.52841	0.031379	OE VS. MT
AT1G28670	0.782706	0.001928	OE VS. MT	AT1G27650	-0.27342	0.031379	OE VS. MT
AT4G16660	0.455865	0.002037	OE VS. MT	AT1G49430	0.516582	0.031379	OE VS. MT
AT5G41880	0.637158	0.002122	OE VS. MT	AT2G20630	0.588496	0.031379	OE VS. MT
AT5G67030	-0.53242	0.002213	OE VS. MT	AT2G40400	0.59297	0.031379	OE VS. MT
AT2G19900	-1.04363	0.002294	OE VS. MT	AT1G71870	0.68442	0.031534	OE VS. MT
AT5G65165	-0.44827	0.002323	OE VS. MT	AT1G09970	-0.34673	0.031682	OE VS. MT
AT1G70985	-1.50369	0.002352	OE VS. MT	AT3G10250	-0.39552	0.031792	OE VS. MT
AT4G19020	0.593747	0.002352	OE VS. MT	AT3G25910	-0.28722	0.031811	OE VS. MT
AT1G53300	0.664187	0.002352	OE VS. MT	AT3G01100	-0.30466	0.031838	OE VS. MT
AT4G28390	-0.57808	0.002391	OE VS. MT	AT5G11740	0.285393	0.03217	OE VS. MT
AT1G31070	0.920123	0.002393	OE VS. MT	AT1G69800	-0.29483	0.032267	OE VS. MT
AT2G37460	0.883665	0.002435	OE VS. MT	AT4G24690	-0.22005	0.032267	OE VS. MT
AT3G51880	-0.43251	0.002446	OE VS. MT	AT3G04630	0.592177	0.032267	OE VS. MT
AT4G27140	-0.96671	0.002488	OE VS. MT	AT2G27380	-0.81228	0.032398	OE VS. MT
AT5G54770	-0.39834	0.002488	OE VS. MT	AT5G67070	0.36708	0.0324	OE VS. MT
AT5G07990	0.782658	0.002489	OE VS. MT	AT5G47900	0.483007	0.03246	OE VS. MT
AT2G47130	-0.55989	0.002565	OE VS. MT	AT2G46950	0.430518	0.032497	OE VS. MT
AT2G29380	-0.42017	0.002565	OE VS. MT	AT3G58850	0.742421	0.032839	OE VS. MT
AT3G22740	-0.98046	0.002688	OE VS. MT	AT1G75370	-0.32378	0.03306	OE VS. MT
AT3G61040	-2.13377	0.002714	OE VS. MT	AT1G26570	0.836925	0.03306	OE VS. MT
AT1G64390	0.45632	0.002714	OE VS. MT	AT1G04560	-0.62629	0.033357	OE VS. MT
AT3G22550	0.780653	0.002877	OE VS. MT	AT2G47420	-0.24839	0.033362	OE VS. MT
AT5G66430	-1.08406	0.002988	OE VS. MT	AT1G56330	0.343997	0.033423	OE VS. MT
AT1G45145	-0.59125	0.003048	OE VS. MT	AT3G26650	0.434224	0.033423	OE VS. MT
AT5G06610	1.15444	0.003087	OE VS. MT	AT1G72790	0.461984	0.033423	OE VS. MT
AT2G43580	-2.05327	0.003132	OE VS. MT	AT3G61440	0.301823	0.033492	OE VS. MT
AT3G12960	-0.99907	0.003132	OE VS. MT	AT4G11910	-0.39141	0.033497	OE VS. MT
AT5G54640	-0.41129	0.00323	OE VS. MT	AT1G48990	-0.35672	0.033535	OE VS. MT
AT1G14930	-0.39382	0.00327	OE VS. MT	AT4G04640	0.286705	0.034085	OE VS. MT
AT2G36880	0.420355	0.00327	OE VS. MT	AT3G48560	-0.25551	0.034197	OE VS. MT
AT5G25830	1.860633	0.00327	OE VS. MT	AT5G53470	-0.28853	0.034317	OE VS. MT
AT4G30490	0.428223	0.003277	OE VS. MT	AT3G50350	0.922122	0.034565	OE VS. MT
AT5G34850	0.408177	0.003307	OE VS. MT	AT5G15350	0.489399	0.034684	OE VS. MT
AT1G19450	0.524799	0.003419	OE VS. MT	AT3G09690	0.304064	0.034769	OE VS. MT
AT1G47128	1.430762	0.003425	OE VS. MT	AT4G02540	0.350281	0.034769	OE VS. MT
AT5G65210	-0.41695	0.003475	OE VS. MT	AT1G31750	-0.54211	0.034788	OE VS. MT
AT4G18020	0.591525	0.003497	OE VS. MT	AT3G58750	0.290802	0.034947	OE VS. MT
AT1G20010	0.482651	0.003515	OE VS. MT	AT1G65010	0.338741	0.035062	OE VS. MT
AT1G73120	0.357868	0.003615	OE VS. MT	AT3G12610	0.567389	0.035062	OE VS. MT
AT4G12420	0.43766	0.003645	OE VS. MT	AT2G47510	0.255015	0.035148	OE VS. MT
AT5G60890	0.513812	0.003652	OE VS. MT	AT5G05290	0.283143	0.035148	OE VS. MT

AT2G45790	0.472305	0.003807	OE VS. MT	AT3G01480	0.348576	0.035148	OE VS. MT
AT5G15580	0.535167	0.00383	OE VS. MT	AT1G65840	0.512321	0.035148	OE VS. MT
AT2G07690	0.538682	0.00383	OE VS. MT	AT3G45320	1.45495	0.03517	OE VS. MT
AT1G07180	-1.04076	0.003932	OE VS. MT	AT5G55410	-0.22325	0.035487	OE VS. MT
AT1G65590	0.942434	0.004046	OE VS. MT	AT1G05510	-1.59123	0.035662	OE VS. MT
AT4G40060	0.80453	0.004165	OE VS. MT	AT2G33580	-0.55428	0.035662	OE VS. MT
AT5G13100	0.726012	0.004207	OE VS. MT	AT5G61780	0.224288	0.035731	OE VS. MT
AT4G22100	-2.53432	0.004233	OE VS. MT	AT5G04420	0.408634	0.035731	OE VS. MT
AT3G11170	0.356673	0.004245	OE VS. MT	AT1G21750	0.295053	0.035795	OE VS. MT
AT2G02120	-0.95635	0.004301	OE VS. MT	AT5G06370	-0.32757	0.035891	OE VS. MT
AT1G51070	0.544568	0.00444	OE VS. MT	AT1G30880	-0.2167	0.035891	OE VS. MT
AT4G02060	0.525971	0.004463	OE VS. MT	AT4G32410	0.249776	0.035891	OE VS. MT
AT1G69410	-0.44211	0.0045	OE VS. MT	AT3G10500	0.327261	0.035891	OE VS. MT
AT4G27130	-0.34445	0.0045	OE VS. MT	AT4G29210	0.337323	0.035891	OE VS. MT
AT3G21250	0.500363	0.0045	OE VS. MT	AT3G20210	0.909285	0.035891	OE VS. MT
AT5G07000	0.575739	0.004555	OE VS. MT	AT2G22420	0.434987	0.035977	OE VS. MT
AT1G43650	-2.31223	0.004568	OE VS. MT	AT3G54400	0.670563	0.035977	OE VS. MT
AT5G40420	-0.94359	0.004576	OE VS. MT	AT5G42100	0.323218	0.036029	OE VS. MT
AT1G03890	-1.63476	0.004626	OE VS. MT	AT1G50010	0.303277	0.036031	OE VS. MT
AT5G04480	0.574351	0.00464	OE VS. MT	AT2G37200	-0.31412	0.036172	OE VS. MT
AT3G43800	0.988475	0.00464	OE VS. MT	AT1G05850	0.297294	0.036392	OE VS. MT
AT3G16770	-0.36215	0.004643	OE VS. MT	AT5G01350	-0.34012	0.036916	OE VS. MT
AT1G49032	-0.55241	0.004649	OE VS. MT	AT2G26910	0.348969	0.037011	OE VS. MT
AT3G01345	-0.94195	0.00477	OE VS. MT	AT3G16120	-0.40819	0.037374	OE VS. MT
AT1G75830	-0.81168	0.004778	OE VS. MT	AT4G31820	0.527693	0.037374	OE VS. MT
AT2G19800	0.546491	0.004778	OE VS. MT	AT4G26700	-0.28187	0.037519	OE VS. MT
AT1G17010	-0.70627	0.004849	OE VS. MT	AT2G19450	-0.25828	0.037723	OE VS. MT
AT1G06110	-0.38742	0.004849	OE VS. MT	AT2G43010	-0.62895	0.037741	OE VS. MT
AT3G14840	0.363763	0.004863	OE VS. MT	AT3G11540	-0.25051	0.037741	OE VS. MT
AT5G51550	0.382674	0.004863	OE VS. MT	AT3G55440	0.264194	0.037826	OE VS. MT
AT5G59870	0.72609	0.004863	OE VS. MT	AT4G18280	-0.91335	0.037915	OE VS. MT
AT5G02750	-0.86167	0.004942	OE VS. MT	AT5G62700	0.236642	0.037915	OE VS. MT
AT1G11670	0.49141	0.004942	OE VS. MT	AT1G68220	-0.36281	0.038164	OE VS. MT
AT4G34790	1.219136	0.004978	OE VS. MT	AT4G22810	0.865249	0.038164	OE VS. MT
AT2G02390	-0.41753	0.005038	OE VS. MT	AT4G39150	0.394216	0.038818	OE VS. MT
AT1G51200	-0.37058	0.005038	OE VS. MT	AT2G33070	-1.92521	0.03895	OE VS. MT
AT5G53260	-1.42241	0.005066	OE VS. MT	AT2G22010	-0.23965	0.03895	OE VS. MT
AT1G70580	-0.64256	0.005066	OE VS. MT	AT5G12250	0.285819	0.03895	OE VS. MT
AT5G56140	-0.43711	0.005066	OE VS. MT	AT4G37930	0.349543	0.03895	OE VS. MT
AT5G49160	0.448046	0.005066	OE VS. MT	AT3G22060	1.116649	0.03895	OE VS. MT
AT4G26050	-0.45055	0.005108	OE VS. MT	AT1G48130	-0.74044	0.039115	OE VS. MT
AT5G14180	-0.23131	0.005108	OE VS. MT	AT1G19570	-0.34425	0.039115	OE VS. MT
AT1G04730	0.724311	0.005108	OE VS. MT	AT3G45310	0.233167	0.039115	OE VS. MT

AT1G62710	-0.34733	0.005115	OE VS. MT	AT3G48700	0.486757	0.039115	OE VS. MT
AT5G47810	-0.41729	0.005348	OE VS. MT	AT5G08020	0.61235	0.039115	OE VS. MT
AT5G26850	0.379157	0.005348	OE VS. MT	AT4G16130	0.221897	0.039415	OE VS. MT
AT2G30500	0.386257	0.005348	OE VS. MT	AT5G54070	-1.89435	0.039723	OE VS. MT
AT1G13170	0.456755	0.005349	OE VS. MT	AT3G60690	-1.05594	0.039741	OE VS. MT
AT1G80180	-0.6204	0.005363	OE VS. MT	AT2G14910	-0.32056	0.039741	OE VS. MT
AT1G69770	0.735662	0.005487	OE VS. MT	AT3G47010	0.411237	0.039741	OE VS. MT
AT3G20250	-0.44887	0.005507	OE VS. MT	AT3G47570	0.453291	0.039741	OE VS. MT
AT1G54410	0.382843	0.005523	OE VS. MT	AT4G01970	-1.72357	0.039747	OE VS. MT
AT1G22340	0.840717	0.005663	OE VS. MT	AT5G55210	-0.40384	0.039747	OE VS. MT
AT4G33980	-0.53257	0.005937	OE VS. MT	AT5G22880	0.447458	0.039888	OE VS. MT
AT2G22080	-0.53925	0.005996	OE VS. MT	AT1G76590	-0.49621	0.039994	OE VS. MT
AT5G54380	0.470504	0.00601	OE VS. MT	AT5G07830	-0.30632	0.039994	OE VS. MT
AT2G41710	-0.43234	0.006038	OE VS. MT	AT2G41070	-0.37011	0.040122	OE VS. MT
AT2G34020	0.524178	0.006139	OE VS. MT	AT2G21820	-0.87231	0.040377	OE VS. MT
AT1G54870	-1.20213	0.006305	OE VS. MT	AT2G02850	-0.37694	0.040377	OE VS. MT
AT4G36860	0.345131	0.006305	OE VS. MT	AT1G79990	0.230115	0.040377	OE VS. MT
AT3G23490	-0.43097	0.006408	OE VS. MT	AT5G37770	0.667851	0.040807	OE VS. MT
AT1G72430	0.944848	0.006625	OE VS. MT	AT5G19150	-0.31948	0.041339	OE VS. MT
AT1G35190	-0.37167	0.006626	OE VS. MT	AT1G70840	-1.48657	0.041401	OE VS. MT
AT5G16380	-0.56775	0.006798	OE VS. MT	AT1G77450	-0.57689	0.041401	OE VS. MT
AT3G48460	0.913134	0.006872	OE VS. MT	AT4G02880	-0.27615	0.041401	OE VS. MT
AT1G07370	0.550936	0.006973	OE VS. MT	AT2G30520	1.662104	0.041444	OE VS. MT
AT1G68060	0.408633	0.006982	OE VS. MT	AT1G21400	-1.41492	0.041484	OE VS. MT
AT5G50170	-0.50515	0.007062	OE VS. MT	AT1G14530	-0.4297	0.041484	OE VS. MT
AT1G26400	-1.84863	0.007118	OE VS. MT	AT3G51860	-0.40096	0.041484	OE VS. MT
AT2G45290	-0.33524	0.007144	OE VS. MT	AT4G16150	-0.29644	0.041484	OE VS. MT
AT2G37930	0.639782	0.007144	OE VS. MT	AT2G40950	-0.25685	0.041484	OE VS. MT
AT3G47340	-1.00567	0.007174	OE VS. MT	AT5G28540	0.317961	0.041484	OE VS. MT
AT1G49580	0.463539	0.007203	OE VS. MT	AT4G13340	0.591077	0.041484	OE VS. MT
AT1G23070	-0.38305	0.007258	OE VS. MT	AT3G22500	-1.40663	0.041556	OE VS. MT
AT2G35190	0.849195	0.007315	OE VS. MT	AT5G65470	-0.29002	0.041556	OE VS. MT
AT4G35100	0.359748	0.007448	OE VS. MT	AT5G43250	0.873649	0.041556	OE VS. MT
AT2G45380	-0.40382	0.007478	OE VS. MT	AT4G26760	0.279059	0.041659	OE VS. MT
AT3G43600	0.394583	0.007493	OE VS. MT	AT4G23850	0.316987	0.041772	OE VS. MT
AT2G42560	-1.25998	0.007549	OE VS. MT	AT4G21326	-0.65184	0.041945	OE VS. MT
AT5G07330	-0.38507	0.007587	OE VS. MT	AT4G36195	-0.31738	0.041945	OE VS. MT
AT4G04020	-0.41704	0.007636	OE VS. MT	AT1G05340	-1.1287	0.042001	OE VS. MT
AT2G25140	-0.35733	0.00775	OE VS. MT	AT1G21780	-0.45825	0.042001	OE VS. MT
AT1G54100	-0.31197	0.00775	OE VS. MT	AT1G56220	-0.35878	0.042001	OE VS. MT
AT3G42850	0.589258	0.00775	OE VS. MT	AT4G21020	-0.28998	0.042001	OE VS. MT
AT3G53230	-0.41188	0.007903	OE VS. MT	AT5G67260	0.259041	0.042001	OE VS. MT
AT5G54970	0.795484	0.007903	OE VS. MT	AT2G29680	0.95486	0.042001	OE VS. MT

AT4G00020	0.748114	0.00813	OE VS. MT	AT5G22290	-0.25401	0.042186	OE VS. MT
AT4G27530	-0.51159	0.0083	OE VS. MT	AT4G30810	0.215842	0.042461	OE VS. MT
AT5G13060	0.867362	0.008698	OE VS. MT	AT4G00820	0.371836	0.042461	OE VS. MT
AT3G20660	-0.54342	0.008752	OE VS. MT	AT5G55135	-2.02543	0.042899	OE VS. MT
AT5G18130	-0.70722	0.008828	OE VS. MT	AT4G15380	-0.33222	0.042899	OE VS. MT
AT3G52880	0.360207	0.008828	OE VS. MT	AT3G19450	0.547489	0.042899	OE VS. MT
AT3G50740	-0.56006	0.009133	OE VS. MT	AT4G16520	-0.40594	0.043113	OE VS. MT
AT3G27060	0.448154	0.009133	OE VS. MT	AT5G59970	0.536325	0.043237	OE VS. MT
AT1G73190	-1.23625	0.009218	OE VS. MT	AT1G68560	0.264679	0.043276	OE VS. MT
AT5G02490	0.453278	0.009218	OE VS. MT	AT3G15580	-0.41708	0.043591	OE VS. MT
AT3G56370	0.579441	0.009218	OE VS. MT	AT2G23240	-1.43022	0.043604	OE VS. MT
AT4G15230	0.613949	0.009218	OE VS. MT	AT1G01480	-1.27623	0.043604	OE VS. MT
AT1G17810	-1.43967	0.009236	OE VS. MT	AT5G05730	0.399313	0.043604	OE VS. MT
AT5G01720	-0.50096	0.009381	OE VS. MT	AT2G18876	0.429974	0.043604	OE VS. MT
AT1G13210	0.379757	0.009455	OE VS. MT	AT2G14520	-0.36528	0.043786	OE VS. MT
AT4G34135	0.548149	0.009455	OE VS. MT	AT2G36310	0.52757	0.044059	OE VS. MT
AT5G01670	-0.87213	0.009469	OE VS. MT	AT1G11475	-0.30589	0.044133	OE VS. MT
AT1G01320	0.263223	0.009469	OE VS. MT	AT1G07530	-0.29711	0.044374	OE VS. MT
AT1G77590	0.571788	0.009469	OE VS. MT	AT5G48160	-0.29654	0.04491	OE VS. MT
AT1G03630	0.58739	0.009469	OE VS. MT	AT2G36460	-0.27038	0.04491	OE VS. MT
AT3G06630	-0.47308	0.009602	OE VS. MT	AT5G04740	0.300742	0.04491	OE VS. MT
AT2G24940	-0.40682	0.009602	OE VS. MT	AT1G54860	-1.15348	0.044933	OE VS. MT
AT1G65340	0.992633	0.009602	OE VS. MT	AT3G50970	-0.78704	0.044933	OE VS. MT
AT2G29300	-1.33626	0.009889	OE VS. MT	AT5G02540	0.722917	0.045143	OE VS. MT
AT4G31830	-0.52825	0.009889	OE VS. MT	AT4G39010	0.673868	0.045225	OE VS. MT
AT1G65090	-0.35055	0.01002	OE VS. MT	AT5G60460	-0.42472	0.045717	OE VS. MT
AT5G43060	0.833229	0.010159	OE VS. MT	AT2G23090	-0.2656	0.045717	OE VS. MT
AT2G24420	-0.34738	0.010286	OE VS. MT	AT1G74670	0.240275	0.046146	OE VS. MT
AT5G35660	-0.98656	0.010298	OE VS. MT	AT3G57020	-0.9058	0.046178	OE VS. MT
AT3G50440	0.90996	0.010475	OE VS. MT	AT2G16850	0.82969	0.046226	OE VS. MT
AT3G09440	0.385461	0.010656	OE VS. MT	AT5G44460	0.467934	0.046268	OE VS. MT
AT2G33590	-0.48875	0.010755	OE VS. MT	AT1G74690	0.292551	0.046359	OE VS. MT
AT1G55200	0.580392	0.010861	OE VS. MT	AT1G19540	-0.44935	0.046495	OE VS. MT
AT2G18540	-0.48818	0.010921	OE VS. MT	AT5G10740	-0.44499	0.046524	OE VS. MT
AT5G54080	-0.51212	0.011256	OE VS. MT	AT1G02890	-0.24539	0.046524	OE VS. MT
AT2G05580	-1.48984	0.011294	OE VS. MT	AT5G16970	-0.28467	0.046547	OE VS. MT
AT2G19590	0.639058	0.011294	OE VS. MT	AT5G66052	-0.60211	0.04689	OE VS. MT
AT3G21380	-1.24018	0.011325	OE VS. MT	AT1G77370	-0.40021	0.046991	OE VS. MT
AT3G62090	-0.68915	0.011325	OE VS. MT	AT2G01440	-0.26303	0.047195	OE VS. MT
AT5G67080	0.485929	0.011325	OE VS. MT	AT1G48430	-0.29192	0.047298	OE VS. MT
AT1G65470	0.685231	0.011325	OE VS. MT	AT5G08240	-0.52782	0.047472	OE VS. MT
AT4G21850	1.449395	0.011325	OE VS. MT	AT5G16460	-0.44173	0.047514	OE VS. MT
AT3G11410	-0.27482	0.011419	OE VS. MT	AT5G05180	1.271637	0.047672	OE VS. MT

AT1G25310	-0.82172	0.011592	OE VS. MT	AT1G10740	0.397381	0.047868	OE VS. MT
AT5G43770	-1.00959	0.011595	OE VS. MT	AT1G02770	-0.48117	0.048164	OE VS. MT
AT1G44900	0.390998	0.011595	OE VS. MT	AT5G66400	-1.13376	0.048182	OE VS. MT
AT3G22490	-1.11481	0.011653	OE VS. MT	AT1G17450	-0.25965	0.048182	OE VS. MT
AT3G02110	0.436033	0.011653	OE VS. MT	AT4G24150	-0.24781	0.048182	OE VS. MT
AT1G65980	-0.27366	0.011696	OE VS. MT	AT5G17230	0.381993	0.048182	OE VS. MT
AT4G25140	-1.18932	0.011773	OE VS. MT	AT1G70800	-0.67987	0.049011	OE VS. MT
AT1G12880	1.575029	0.011773	OE VS. MT	AT4G31490	0.241619	0.049412	OE VS. MT

MT: mutant.

WT: Col-0.

OE: Overexpression.

Yellow shading gene: shared genes of MT vs. WT and MT vs. OE.

Supplementary Table 6. KEGG enrichment analysis of shared DEGs in supplementary Table 5.

KEGG pathway	Input number	Background number	*p*-value	Corrected *p*-value	Input genes
Glucosinolate biosynthesis	4	23	3.21E-09	4.14E-07	AT1G24100<br>AT2G20610<br>AT1G74100<br>AT4G31500
Tryptophan metabolism	4	60	1.14E-07	7.37E-06	AT1G24100<br>AT2G20610<br>AT1G74100<br>AT4G31500
2-Oxocarboxylic acid metabolism	4	74	2.55E-07	1.1E-05	AT1G24100<br>AT2G20610<br>AT1G74100<br>AT4G31500
Metabolic pathways	10	2246	1.18E-06	3.79E-05	AT2G38860<br>AT2G20610<br>AT3G16150<br>AT1G74100<br>AT4G31500<br>AT2G30860<br>AT2G30870<br>AT1G24100<br>AT2G14750<br>AT3G23820
Biosynthesis of secondary metabolites	5	1107	0.000904	0.007779	AT1G24100<br>AT2G20610<br>AT3G16150<br>AT1G74100<br>AT4G31500
Glutathione metabolism	2	102	0.00242	0.015612	AT2G30860<br>AT2G30870
Sulfur metabolism	1	42	0.029784	0.054888	AT2G14750
Alanine aspartate and glutamate metabolism	1	51	0.03591	0.058638	AT3G16150
Cyanoamino acid metabolism	1	69	0.04805	0.071247	AT3G16150
Phagosome	1	82	0.056727	0.081309	AT1G20010
Pyruvate metabolism	1	86	0.059382	0.084179	AT2G38860
Purine metabolism	1	100	0.068617	0.092204	AT2G14750
Amino sugar and nucleotide sugar metabolism	1	131	0.088758	0.115654	AT3G23820

Databases: KEGG PATHWAY.

Statistical test method: hypergeometric test / Fisher's exact test.

FDR correction method: Benjamini and Hochberg.

Supplementary Table 7. Descriptions of 19 environmental factors.

Name	Description
bio1	Annual mean temperature
bio2	Mean diurnal range (mean of monthly (max temp - min temp))
bio3	Isothermality (bio02/bio07)
bio4	Temperature seasonality (Standard deviation * 100)
bio5	Max temperature of warmest month
bio6	Min temperature of coldest month
bio7	Temperature annual range (bio05-bio06)
bio8	Mean temperature of wettest quarter
bio9	Mean temperature of driest quarter
bio10	Mean temperature of warmest quarter
bio11	Mean temperature of coldest quarter
bio12	Annual precipitation
bio13	Precipitation of wettest month
bio14	Precipitation of driest month
bio15	Precipitation seasonality
bio16	Precipitation of wettest quarter
bio17	Precipitation of driest quarter
bio18	Precipitation of warmest quarter
bio19	Precipitation of coldest quarter

Supplementary Table 8. Values of 19 environmental factors of *SWK*-present accessions (n = 55) and *SWK*-absent accessions (n = 5) in 60 accessions from African population (Morocco) for *T*-test analysis.

**Accession**	**Whether *SWK* exists**	**Bio1**	**Bio2**	**Bio3**	**Bio4**	**Bio 5**	**Bio 6**	**Bio 7**	**Bio 8**	**Bio 9**	**Bio 10**	**Bio 11**	**Bio 12**	**Bio 13**	**Bio 14**	**Bio 15**	**Bio 16**	**Bio 17**	**Bio 18**	**Bio 19**
Azr5	No	12.2038	11.721	38.673	650.711	29.456	-0.852	30.308	7.76667	20.4793	20.6747	4.684	369	48	5	45.39	133	32	32	109
IFr0	No	11.9545	11.623	39.14	630.569	28.772	-0.924	29.696	7.676	19.944	20.1653	4.642	383	50	5	47.07	140	31	31	116
Ifr3	No	11.9545	11.623	39.14	630.569	28.772	-0.924	29.696	7.676	19.944	20.1653	4.642	383	50	5	47.07	140	31	31	116
Ifr4	No	11.9545	11.623	39.14	630.569	28.772	-0.924	29.696	7.676	19.944	20.1653	4.642	383	50	5	47.07	140	31	31	116
Ait9	No	9.51783	11.522	37.9902	656.839	26.408	-3.92	30.328	5.22733	17.8747	17.8747	1.78667	560	74	4	54.39	207	30	30	185
Agl0	Yes	11.8948	11.59	38.1462	659.025	28.992	-1.392	30.384	7.396	20.29	20.432	4.28	410	53	5	47.43	150	33	33	127
Agl1	Yes	11.8948	11.59	38.1462	659.025	28.992	-1.392	30.384	7.396	20.29	20.432	4.28	410	53	5	47.43	150	33	33	127
Agl2	Yes	11.8948	11.59	38.1462	659.025	28.992	-1.392	30.384	7.396	20.29	20.432	4.28	410	53	5	47.43	150	33	33	127
Agl3	Yes	11.8948	11.59	38.1462	659.025	28.992	-1.392	30.384	7.396	20.29	20.432	4.28	410	53	5	47.43	150	33	33	127

Agl5	Yes	11.8948	11.59	38.1462	659.025	28.992	-1.392	30.384	7.396	20.29	20.432	4.28	410	53	5	47.43	150	33	33	127
Agl9	Yes	11.8948	11.59	38.1462	659.025	28.992	-1.392	30.384	7.396	20.29	20.432	4.28	410	53	5	47.43	150	33	33	127
Ait14	Yes	9.51783	11.522	37.9902	656.839	26.408	-3.92	30.328	5.22733	17.8747	17.8747	1.78667	560	74	4	54.39	207	30	30	185
Arb0	Yes	15.1982	12.028	40.0016	623.19	31.504	1.436	30.068	11.1353	23.0213	23.152	7.78333	451	66	2	59.99	181	19	21	152
Arb2	Yes	15.1982	12.028	40.0016	623.19	31.504	1.436	30.068	11.1353	23.0213	23.152	7.78333	451	66	2	59.99	181	19	21	152
Azr0	Yes	12.2038	11.721	38.673	650.711	29.456	-0.852	30.308	7.76667	20.4793	20.6747	4.684	369	48	5	45.39	133	32	32	109
Azr11	Yes	12.2038	11.721	38.673	650.711	29.456	-0.852	30.308	7.76667	20.4793	20.6747	4.684	369	48	5	45.39	133	32	32	109
Azr13	Yes	12.2038	11.721	38.673	650.711	29.456	-0.852	30.308	7.76667	20.4793	20.6747	4.684	369	48	5	45.39	133	32	32	109
Azr16	Yes	12.2038	11.721	38.673	650.711	29.456	-0.852	30.308	7.76667	20.4793	20.6747	4.684	369	48	5	45.39	133	32	32	109
Azr7	Yes	12.2038	11.721	38.673	650.711	29.456	-0.852	30.308	7.76667	20.4793	20.6747	4.684	369	48	5	45.39	133	32	32	109
Bab0	Yes	14.2608	11.367	39.0297	615.974	30.968	1.844	29.124	7.78333	22.164	22.1707	7.12933	943	166	1	79.42	480	15	22	422
Bab3	Yes	14.2608	11.367	39.0297	615.974	30.968	1.844	29.124	7.78333	22.164	22.1707	7.12933	943	166	1	79.42	480	15	22	422

Bba0	Yes	14.2608	11.367	39.0297	615.974	30.968	1.844	29.124	7.78333	22.164	22.1707	7.12933	943	166	1	79.42	480	15	22	422
Bba2	Yes	12.3257	10.984	37.5496	635.277	29.084	-0.168	29.252	7.96467	20.4133	20.4647	4.90933	390	49	6	45.17	133	33	35	124
Bbe0	Yes	13.1253	11.199	38.8897	610.319	29.724	0.928	28.796	6.822	21.0307	21.0307	6.03933	836	142	1	77.5	405	14	14	337
Elh10	Yes	14.9653	11.695	39.3813	624.364	31.1	1.404	29.696	10.8887	22.818	22.9113	7.544	455	66	2	60.26	183	19	21	154
Elh15	Yes	14.9653	11.695	39.3813	624.364	31.1	1.404	29.696	10.8887	22.818	22.9113	7.544	455	66	2	60.26	183	19	21	154
Elh2	Yes	14.9653	11.695	39.3813	624.364	31.1	1.404	29.696	10.8887	22.818	22.9113	7.544	455	66	2	60.26	183	19	21	154
Elh20	Yes	14.9653	11.695	39.3813	624.364	31.1	1.404	29.696	10.8887	22.818	22.9113	7.544	455	66	2	60.26	183	19	21	154
Elh23	Yes	14.9653	11.695	39.3813	624.364	31.1	1.404	29.696	10.8887	22.818	22.9113	7.544	455	66	2	60.26	183	19	21	154
Elh27	Yes	14.9653	11.695	39.3813	624.364	31.1	1.404	29.696	10.8887	22.818	22.9113	7.544	455	66	2	60.26	183	19	21	154
Elh33	Yes	14.9653	11.695	39.3813	624.364	31.1	1.404	29.696	10.8887	22.818	22.9113	7.544	455	66	2	60.26	183	19	21	154
Elh39	Yes	14.9653	11.695	39.3813	624.364	31.1	1.404	29.696	10.8887	22.818	22.9113	7.544	455	66	2	60.26	183	19	21	154
Elh46	Yes	14.9653	11.695	39.3813	624.364	31.1	1.404	29.696	10.8887	22.818	22.9113	7.544	455	66	2	60.26	183	19	21	154
Elk1	Yes	12.994	11.65	38.3577	661.439	30.032	-0.34	30.372	8.39133	21.4147	21.66	5.492	531	71	4	58.04	204	27	28	188

Elk20	Yes	12.994	11.65	38.3577	661.439	30.032	-0.34	30.372	8.39133	21.4147	21.66	5.492	531	71	4	58.04	204	27	28	188
Elk28	Yes	12.994	11.65	38.3577	661.439	30.032	-0.34	30.372	8.39133	21.4147	21.66	5.492	531	71	4	58.04	204	27	28	188
Elk3	Yes	12.994	11.65	38.3577	661.439	30.032	-0.34	30.372	8.39133	21.4147	21.66	5.492	531	71	4	58.04	204	27	28	188
Ifr6	Yes	11.9545	11.623	39.14	630.569	28.772	-0.924	29.696	7.676	19.944	20.1653	4.642	383	50	5	47.07	140	31	31	116
Ket10	Yes	12.9692	11.129	39.0765	598.008	29.5	1.02	28.48	6.708	20.7133	20.7133	6.02667	809	126	2	72.24	365	20	20	339
Ket12	Yes	12.9692	11.129	39.0765	598.008	29.5	1.02	28.48	6.708	20.7133	20.7133	6.02667	809	126	2	72.24	365	20	20	339
Khe0	Yes	13.2147	12.285	39.9527	650.238	30.428	-0.32	30.748	8.72733	21.4833	21.6933	5.75933	451	58	5	51.18	168	31	31	149
Khe32	Yes	13.2147	12.285	39.9527	650.238	30.428	-0.32	30.748	8.72733	21.4833	21.6933	5.75933	451	58	5	51.18	168	31	31	149
Meh0	Yes	13.9942	11.778	38.9135	643.187	31.244	0.976	30.268	9.628	22.158	22.212	6.464	372	49	6	47	130	29	31	120
Meh4	Yes	13.9942	11.778	38.9135	643.187	31.244	0.976	30.268	9.628	22.158	22.212	6.464	372	49	6	47	130	29	31	120
Meh7	Yes	13.9942	11.778	38.9135	643.187	31.244	0.976	30.268	9.628	22.158	22.212	6.464	372	49	6	47	130	29	31	120
Oua0	Yes	12.6863	12.769	38.9546	707.544	31.204	-1.576	32.78	8.74867	21.714	21.8267	4.59067	411	56	4	53.03	148	24	32	133
Set0	Yes	11.9843	11.459	38.1752	644	28.404	-1.612	30.016	7.83933	20.1367	20.1367	4.32	496	67	3	57.42	189	23	23	168

Set6	Yes	11.9843	11.459	38.1752	644	28.404	-1.612	30.016	7.83933	20.1367	20.1367	4.32	496	67	3	57.42	189	23	23	168
Tah0	Yes	14.1455	10.804	38.009	611.498	30.296	1.872	28.424	8.07267	21.9	22.048	7.03867	439	59	5	53.59	162	26	28	157
Tah4	Yes	14.1455	10.804	38.009	611.498	30.296	1.872	28.424	8.07267	21.9	22.048	7.03867	439	59	5	53.59	162	26	28	157
Taz0	Yes	12.4195	11.028	37.8149	631.835	29.132	-0.032	29.164	6.13867	20.4747	20.5053	5.03533	421	53	6	47.35	147	33	36	139
Tanz-1	Yes	23.2643	11.887	72.1494	140.252	31.724	15.248	16.476	24.5747	21.258	24.7313	21.258	427	100	0	93.58	219	1	162	1
Taz11	Yes	12.4195	11.028	37.8149	631.835	29.132	-0.032	29.164	6.13867	20.4747	20.5053	5.03533	421	53	6	47.35	147	33	36	139
Taz16	Yes	12.4195	11.028	37.8149	631.835	29.132	-0.032	29.164	6.13867	20.4747	20.5053	5.03533	421	53	6	47.35	147	33	36	139
Taz18	Yes	12.4195	11.028	37.8149	631.835	29.132	-0.032	29.164	6.13867	20.4747	20.5053	5.03533	421	53	6	47.35	147	33	36	139
Til2	Yes	13.401	13.119	39.6382	705.439	31.988	-1.108	33.096	9.44867	22.3893	22.4927	5.29733	410	56	4	53.77	149	24	30	134
Tiz0	Yes	12.7225	11.23	37.7037	649.622	29.796	0.012	29.784	11.2567	20.9867	20.9867	5.112	336	44	7	40.69	117	34	34	96
Tiz7	Yes	12.7225	11.23	37.7037	649.622	29.796	0.012	29.784	11.2567	20.9867	20.9867	5.112	336	44	7	40.69	117	34	34	96
Zin4	Yes	17.1595	10.142	39.7863	531.134	31.092	5.6	25.492	10.8847	23.88	24.0133	10.8847	824	158	0	87.67	442	9	16	442

Zin9	Yes	17.1595	10.142	39.7863	531.134	31.092	5.6	25.492	10.8847	23.88	24.0133	10.8847	824	158	0	87.67	442	9	16	442
t-test *p*-value	Yes	0.02744	0.2838	0.79152	0.69615	0.00209	0.08313	0.2067	0.16728	0.00178	0.00339	0.07843	0.2963	0.2596	0.0037	0.002	0.022	1.53877E-06	0.66	0.289

Blue background: SWK is absent.

Yellow background: Two-sided Student's *t*-test, p-value < 0.05.

Supplementary Table 9. List of primers used in this study.

Primer	Sequence (5'-3')	Experiment
G1-F	CCCCCCGGGATGTGTGGTTTTCACTTC	35S-G1-GFP construction
G1-R	CGGGTCGACTGAAATAAGATGTAGAGA	35S-G1-GFP construction
G2-F	CCCCCCGGGATGCGTAGAGTTGTCATT	35S-G2-GFP construction
G2-R	CGGGTCGACCTCGGAATCGGAATCGGA	35S-G2-GFP construction
G3-F	CCCCCCGGGATGCTGCTTTGGAATGGT	35S-G3-GFP construction
G3-R	CGGGTCGACTTTAACTGAAATATTAACCAC	35S-G3-GFP construction
G4-F	CGGGTCGACATGACAAGCCAAATAAGACAAG	35S-G4-GFP construction
G4-R	CGGCCCGGGCCTTAGCACTGATTCTGG	35S-G4-GFP construction
G5-F	CGGGTCGACATGAATAGCACGGCCAATGACG	35S-G5-GFP construction
G5-R	CGGCCCGGGGGGTTTTGACCAAAAGATATG	35S-G5-GFP construction
G6-F	CCCCCCGGGATGATGATACGGGGTGGT	35S-G6-GFP construction
G6-R	CGGGTCGACCATACTCATGGCGTATGACG	35S-G6-GFP construction
G7-F	CGGGTCGACATGATTGGTTTCTTCCTCCAGC	35S-G7-GFP construction
G7-R	CGGCCCGGGACTTTTGATCAAAGACGAAAAG	35S-G7-GFP construction
CRISPR-SWK-target1-F	TCCGGTAAAAACAAACCGGAgttttagagctagaaat	*SWK* knockout mutants plasmid construction
CRISPR-SWK-target1-R	TCCGGTTTGTTTTTACCGGACaatctcttagtcgact	*SWK* knockout mutants plasmid construction
CRISPR-SWK-target2-F	TGACGGCAAGAACACGACAAgttttagagctagaaat	*SWK* knockout mutants plasmid construction
CRISPR-SWK-target2-R	TTGTCGTGTTCTTGCCGTCACaatcactacttcgtct	*SWK* knockout mutants plasmid construction
CRISPR-SWK-target3-F	TCACTTCCACACAAAAACGTgttttagagctagaaat	*SWK* knockout mutants plasmid construction
CRISPR-SWK-target3-R	ACGTTTTTGTGTGGAAGTGATgaccaatggtgctttg	*SWK* knockout mutants plasmid construction
CRISPR-SWK-target4-F	TTTAACCAAACCTTAGACGCgttttagagctagaaat	*SWK* knockout mutants plasmid construction
CRISPR-SWK-target4-R	GCGTCTAAGGTTTGGTTAAATgaccaatgttgctcc	*SWK* knockout mutants plasmid construction
G1-F	ATCTCTGCATGCGGTGAAGG	RT-qPCR
G1-R	CAACGAAGCCCAAACAAGAGG	RT-qPCR
G2-F	TCCAAGTTATTTCCCAGACACGCGG	RT-qPCR
G2-R	CGCTGTCCTTCAGTCCTCCTAA	RT-qPCR
G3-F	CACGTGGCTTGTTGTTGGAGT	RT-qPCR
G3-R	TATGGATAAAGGACATGACATCATC	RT-qPCR
G4-F	CCAACACCATCAACCCTACAAC	RT-qPCR

G4-R	CTACCTTAGCACTGATTCTGGC	RT-qPCR
G5-F	GCCAATGACGATGAAAGGATGA	RT-qPCR
G5-R	GACCAAAAGATATGAGCTGCCG	RT-qPCR
G6-F	GACTTCCCAAGACAAGAGAGAATG	RT-qPCR
G6-R	ACGTTCGTGAGATACAATTATGGG	RT-qPCR
G7-F	TTTGTCTCTCGTAGTTGGATTCTTT	RT-qPCR
G7-R	TTGCTTATAAATATATATCACGCTC	RT-qPCR
GSTF9-F	ACAAAATCTTCGAGTCCCGT	RT-qPCR
GSTF9-R	GTGGTCGCTTCCACATCAAG	RT-qPCR
GSTF10-F	GCTGTTGTGACATTGGTGGAG	RT-qPCR
GSTF10-R	TGGGATTTTACCGAAAAGGCTGA	RT-qPCR
cytHPPK/DHPS-F	ATTTGTGGTCGTCCTGAGGC	RT-qPCR
cytHPPK/DHPS-R	ATAGCCTTGCCGCGTCTAC	RT-qPCR
DHFS-F	TTCGCGATCAAGGATGTGGG	RT-qPCR
DHFS-R	CAAGAAGCACTGTTGCTCCG	RT-qPCR
DHFR1-F	TGCATGCTGGCTCATGTGTG	RT-qPCR
DHFR1-R	CCGGGTTTATCTTCATTACAGGAAA	RT-qPCR
DHFR2-F	CACCAAAACCTTTTCCTGTTTTGAA	RT-qPCR
DHFR2-R	AGAAATTGATTAGGGGCTAAACAGC	RT-qPCR
FPGS B-F	GATGCCACCAATGCGGTTC	RT-qPCR
FPGS B-R	ATTCCAGCCTTCTCACCAGC	RT-qPCR
FPGS C-F	AATCCAAGGGACTGATGCGG	RT-qPCR
FPGS C-R	AAGGCATTACGGCACTGGAA	RT-qPCR
FPGS D-F	CAGAGACTCGGGCTTCAAGG	RT-qPCR
FPGS D-R	CGGGTAAACCGTTTGTCTGA	RT-qPCR
ACTIN8-F	GGTAACATTGTGCTCAGTGGTGG	RT-qPCR
ACTIN8-R	GGTGCAACGACCTTAATCTTCAT	RT-qPCR
